# Retrospective evaluation of an intervention based on training sessions to increase the use of control charts in hospitals

**DOI:** 10.1136/bmjqs-2021-013514

**Published:** 2022-06-24

**Authors:** Laura Kudrna, Paul Bird, Karla Hemming, Laura Quinn, Kelly Schmidtke, Richard Lilford

**Affiliations:** 1 Institute of Applied Health Research, University of Birmingham, Birmingham, UK; 2 University Hospitals Birmingham NHS Foundation Trust, Birmingham, UK; 3 West Midlands Academic Health Science Network, Birmingham, UK; 4 Warwick Medical School, University of Warwick, Coventry, UK

**Keywords:** statistics, statistical process control, quality improvement, medical education, team training

## Abstract

**Background:**

Statistical process control charts (SPCs) distinguish signal from noise in quality and safety metrics and thus enable resources to be targeted towards the most suitable actions for improving processes and outcomes. Nevertheless, according to a recent study, SPCs are not widely used by hospital boards in England. To address this, an educational training initiative with training sessions lasting less than one and a half days was established to increase uptake of SPCs in board papers. This research evaluated the impact of the training sessions on the inclusion of SPCs in hospital board papers in England.

**Methods:**

We used a non-randomised controlled before and after design. Use of SPCs was examined in 40 publicly available board papers across 20 hospitals; 10 intervention hospitals and 10 control hospitals matched using hospital characteristics and time-period. Zero-inflated negative binomial regression models and t-tests compared changes in usage by means of a difference in difference approach.

**Results:**

Across the 40 board papers in our sample, we found 6287 charts. Control hospitals had 9/1585 (0.6%) SPCs before the intervention period and 23/1900 (1.2%) after the intervention period, whereas intervention hospitals increased from 89/1235 (7%) before to 328/1567 (21%) after the intervention period; a relative risk ratio of 9 (95% CI 3 to 32). The absolute difference in use of SPCs was 17% (95% CI 6% to 27%) in favour of the intervention group.

**Conclusions:**

The results suggest that a scalable educational training initiative to improve use of SPCs within organisations can be effective. Future research could aim to overcome the limitations of observational research with an experimental design or seek to better understand mechanisms, decision-making and patient outcomes.

**Key messages**

**What is already known on this topic**

- Statistical process control charts (SPCs) provide a basis for quality management and enable resources to be targeted effectively. Earlier research suggests that many hospital governing bodies, known as hospital boards in England, do not use SPCs.

**What this study adds**

- An educational initiative with training sessions is ongoing to stimulate the demand for and supply of SPCs. This study reports positive findings of a controlled before and after study on the effectiveness of the intervention using naturally occurring observational data from board meeting papers.

**How this study might affect research, practice and/or policy**

- Our results were not likely due to a ‘rising tide’ of greater use of SPCs, which suggests that focused interventions supporting uptake may still be required. Future research should consider mechanisms and use an experimental design.

## Introduction

### Rationale for the use of statistical process control charts (SPCs)

**Figure 1:**
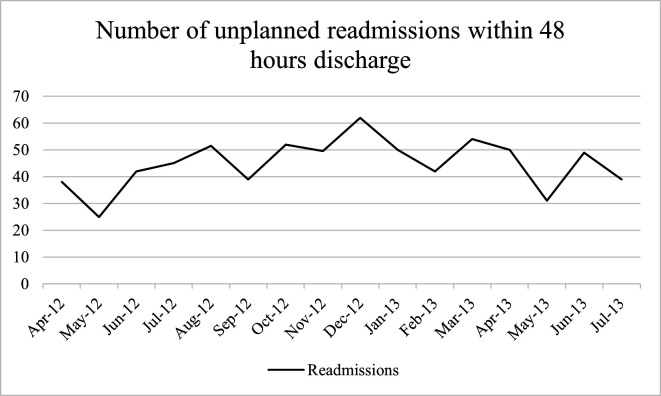
Drawn from real hospital data presented in Schmidtke *et al*.^17^ Time series chart showing the number of unplanned readmissions within 48 hours of discharge from April 2012 until July 2013 at a single hospital.

**Figure 2:**
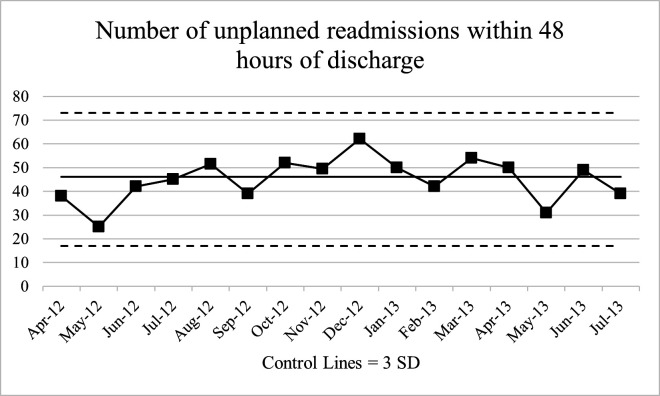
Drawn from real hospital data presented in Schmidtke *et al*.^17^ SPC showing the number of unplanned readmissions within 48 hours of discharge from April 2012 until July 2013 at a single hospital. SPC, statistical process control chart.

The principles underlying statistical process control charts (SPCs) have been fundamental tenets of safety science since they were promoted by Deming and Shewhart in the 1930s.^1 2^ Originally developed to drive quality improvement in manufacturing, SPCs are now widely recommended for use in healthcare.^3^ A key feature of SPCs is ‘process’ or ‘control’ limits (henceforth used interchangeably) that visualise statistical variation from a mean. SPCs thus distinguish signal from noise or, in Deming and Shewhart’s original terminology, special cause from common cause variation. As a result, attention can be focused where it is needed. Presenting data in SPCs improve the ability of public advisors and hospital decision-makers to make good decisions given variation in the data, for instance, by not over-reacting to variation that is typical for a particular process of care.^4^ Examples of charts without and with process limits are shown in figures 1 and 2, respectively. Including process limits can limit the influence of cognitive biases that may otherwise guide decision-making. For example, in ‘anchoring bias’, human attention anchors on the most extreme and recent data points in a time-series chart, regardless of whether these data lie within common cause variation.^4 5^ A recent randomised trial showed that the use of SPCs was associated with fewer adverse surgical outcomes.^6^ Thus, omitting information about statistical variation could compromise decision-making about process variation, instigate unnecessary intervention, and, consequently, lead to the inefficient allocation of resources.

### Lack of adoption of control chart methods

Hospital boards in the English National Health Service (NHS) are made up of executive and non-executive members who have a duty to assure the quality and safety of services. Board papers, therefore, include charts displaying quality and safety metrics. A previous study investigated the prevalence of SPCs in the documents used by hospital board members (board papers) in England, UK. The findings showed that SPCs are not widely included in hospital board papers in England: in 30 randomly selected English acute care hospitals’ quality and safety board papers, nearly half (14/30, 47%) of board papers did not contain any SPCs and only 12% (72 of 589) of the charts across papers were SPCs.^7^ Although the inclusion of SPCs in board papers does not necessarily indicate that these charts are being used effectively, it does suggest engagement with aspects of the approach.

### An intervention to improve use of SPCs in board papers

The above findings underpinned the NHS Improvement/England (NHS I/E) (2019) initiative called ‘Making Data Count’ that encourages NHS institutions to adopt SPCs.^8^ NHS I/E is the organisation responsible for driving up the standard of care in the NHS. The initiative is comprised of educational resources and training sessions which take less than one and a half days to deliver, as described below in the “intervention” section.

### Study aims

**Figure 3:**
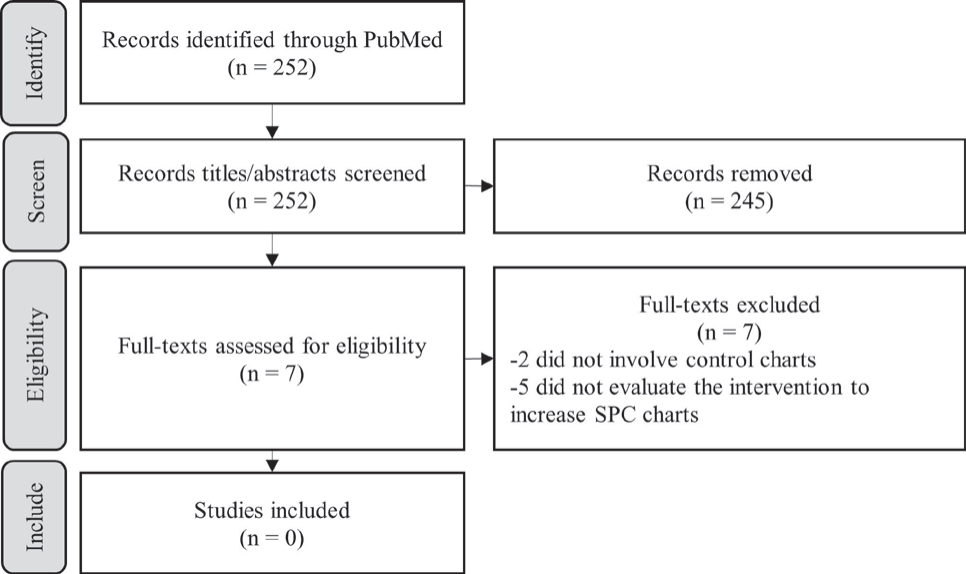
Results of systematic review seeking studies on training interventions to increase the use of SPCs for routine monitoring within institutions. SPC, statistical process control chart.

The research aimed to assess the effect of the Making Data Count training sessions on the appearance of SPCs in publicly available board papers from NHS hospitals and to assess perceptions of the sessions among attendees. We conducted a systematic search for initiatives that aimed to improve use of SPCs for routine surveillance in healthcare. Our search strategy is laid out in figure 3 and discussed in the study protocol (online supplemental file 1). We looked for studies using SPCs in routine surveillance (rather than within an intervention to improve a given process)^9^ and found no papers replicating our approach.

10.1136/bmjqs-2021-013514.supp1 Supplementary data

## Methods

A study protocol detailing the methods was published on the Open Science Foundation^10^ (online supplemental file 1). The SQUIRE reporting guideline checklist^11^ was completed (online supplemental file 2).

10.1136/bmjqs-2021-013514.supp2 Supplementary data

### Context

NHS Improvement delivered Making Data Count training sessions to NHS hospital board members and hospital analysts from November 2017. Hospital recruitment was performed by snowball sampling, where information on the training sessions was disseminated using social media, email and word of mouth.

### Intervention

The TIDier checklist^12^ was completed (online supplemental file 3). The Making Data Count training sessions were delivered to two groups of hospital staff. One group was board members who received sessions lasting around 90 min. The second group were quality improvement staff, including analysts, clinicians and operational staff, and their training took place over one working day. The training sessions aimed to improve knowledge about SPCs and increase their uptake (see online supplemental files 4 and 5 for training PowerPoints). Content included background on SPCs, when and how to use them, how they can be generated and how they can inform decision-making about process variation. Topics included identifying trends, special versus common cause variation and using icons to summarise trends. The limitations of other charts were discussed, and, importantly, each training session was personalised using hospitals’ own data. No specific software platform was recommended for creating SPCs, but the training team provided tools in Excel and SQL software that could be adapted by the trainees. If trainees requested further tools, the training team provided details about other organisations that could provide information on other software tools such as Business Objects, Tableau and Qlik.

10.1136/bmjqs-2021-013514.supp3 Supplementary data

10.1136/bmjqs-2021-013514.supp4 Supplementary data

10.1136/bmjqs-2021-013514.supp5 Supplementary data

### Study of the intervention

#### Sample size

Our sample size was based on detecting a 30 percentage-point improvement in the proportion of SPCs from 10% preintervention to 40% postintervention. Given that the effectiveness of the training intervention on patient safety is contingent on changes in the uptake of SPCs in board papers, we believed that at least a ‘moderate’ effect size^13^ would be necessary to stimulate widespread adoption. Assuming 5% significance and 80% power, and assuming a correlation between preintervention and postintervention measures of 0.90 based on a t-test,^14^ a minimum of 16 hospitals in total with preintervention and postintervention measures was required (eight in each arm). We included 20 hospitals to err on the side of caution.

#### Hospital selection

We selected 10 acute care hospitals that received the training after February 2018. To achieve temporal heterogeneity, we sampled one training intervention hospital per month. If more than one hospital received the training intervention in each month, we randomly selected one of the hospitals. We then selected matched control hospitals that had not received the training using the NHS Digital Peer Finder tool.^15^ Hospitals were matched on the number of patient attendances, degree of specialisation and deprivation level. Degree of specialisation was defined as the divergence of individual trust Healthcare Resource Group activity profile from the national profile.^15^ Deprivation level was obtained from the average 2010 Index of Multiple Deprivation score in Lower Super Output Areas (containing about 1500 people) where the hospitals’ patients live.^16^ Tiebreaker characteristics were number of full-time equivalent staff, urban location and whether the hospital had been classified as a ‘foundation hospital’ by NHS authorities.

#### Board paper selection

**Figure 4:**
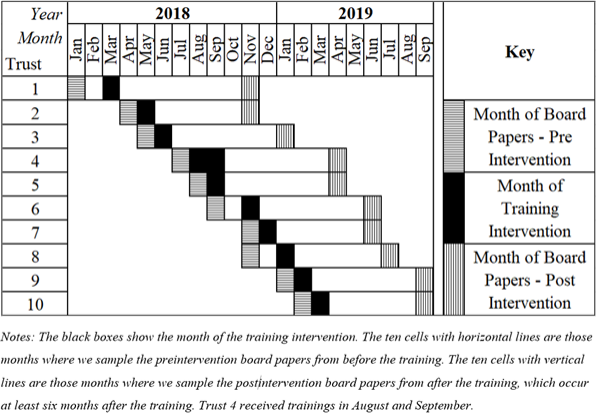
Selected board papers for preintervention and postintervention periods, and month of training intervention, for 10 acute hospitals that received ‘Making Data Count’ training sessions.

For the intervention hospitals, the preintervention board paper was the first paper published at least 1 month before the training intervention. The postintervention board paper was the first board paper published at least 6 months after the intervention. The papers from the control hospitals were selected at the closest month to their matched intervention hospitals. Figure 4 shows the study design with 20 observations for the intervention hospitals (10 preintervention and 10 postintervention) and 20 observations for the matched control hospitals (again 10 preintervention and 10 postintervention), giving a total sample of 40 board papers across 20 hospitals.

#### Quantitative measures: intervention versus control hospitals

In line with previous research on use of SPCs in board papers,^17^ our main outcome measure was the proportion of all charts in the board papers made up of SPCs. There were three other outcomes: first, the proportion of quality and safety charts made up of SPCs; second, the proportion of time series charts made up of SPCs and third, the proportion of time series *and* between groups charts made up of SPCs (between group charts include funnel charts that show data between hospitals).

#### Quantitative measures: examination of SPCs in intervention and control hospitals

We examined SPCs included in board papers of the intervention and control hospitals for inclusion of certain specific factors included in the training for intervention hospitals (see PowerPoint slides in online supplemental file 4). One factor was icons (slide 47) that summarise statistical variation visually using colours and letters that indicate special or common cause variation or indicate performance relative to a target. Another was if the control limits were labelled (slides 32, 34). See online supplemental file 6 for the coding frame.

10.1136/bmjqs-2021-013514.supp6 Supplementary data

#### Quantitative coding

Four independent reviewers (R1, R2, R3, R4) conducted the quantitative coding. In step one, R1 and R2 independently identified charts and classified them according to whether they were quality and safety charts. In step two, R2 removed information regarding the hospital and the board meeting date. In step three, R3 and R4 identified the types of charts and specific elements of SPCs if identified. Any deblinding was reported.

#### Qualitative measures

The qualitative measures were four questions asked after the training sessions in feedback forms: ‘What went well today?’, ‘What could have been done differently?’, ‘What are your key takeaways?’ and ‘Any other comments about today?’. These forms were designed and administered by NHS-I/E and made available to the research team.

### Analysis

Hospital characteristics were summarised using means and SD. Inter-rater reliability was calculated using kappa statistics. Information regarding the type of charts and features of SPCs (online supplemental file 6) was summarised using counts and proportions. Next, we examined the effect of the training intervention on the main outcomes. For all hospitals, we first summarised the number of SPCs (outcome), the total number of charts and the proportion of SPCs out of all charts. The difference in the proportion of SPCs between preintervention and postintervention was computed for each hospital. This information was stratified by intervention and control hospitals, compared using a t-test and represented as a difference in difference with 95% CI.

To determine the relative effect (risk ratio) of the intervention, we fit a cluster-level analysis using zero-inflated negative Binomial regression model (as outcome data contain a high number of zero counts and there was overdispersion), with the outcome the number of SPCs in the postintervention period, fixed categorical effects for the intervention, the proportion of SPCs in preintervention period and an exposure of all charts in the postintervention period.

In sensitivity analyses (see online supplemental file 7), we explored other models. The analyses presented as our primary analysis (zero-inflated negative Binomial) differed to that planned (Poisson) due to many hospitals having no SPCs (high number of zero counts).

10.1136/bmjqs-2021-013514.supp7 Supplementary data

For the qualitative responses, a thematic analysis was conducted to identify barriers to and facilitators of using SPCs.^18^ We used an inductive, semantic and (critical) realist approach. One researcher coded each response into the main theme present in the data. These were reviewed by a second researcher who discussed the codes with the first researcher.

## Results

### Hospital characteristics

Information about the 20 hospitals from the NHS Digital Peer Finder Tool^15^ at baseline is summarised in table 1. On average, there were slightly more patient attendances per year in the intervention hospitals (1.7 mil, SD=0.5 mil) than in the matched control hospitals (1.3 mil, SD=0.75 mil). The degree of specialisation score was lower on average in the intervention group (83 739, SD=80 639) than in the matched control group (138 747, SD=135 068). The average 2010 Index of Multiple Deprivation was similar, at 24 (SD=7) in the intervention and 23 (SD=5) in the matched control sample.

**Table 1:** Hospital characteristics. means with SD in parentheses

	Intervention	Matched control	Overall
	N=10	N=10	N=20
Attendances	1 167 058 (506 825)	1 341 442 (750 439)	1 254 250 (646 233)
Degree of specialisation	83 739 (80 639)	138 747 (135 068)	105 623 (113 366)
Deprivation	24 (7)	23 (5)	23 (6)

Further details available from NHS Digital Per Finder Tool.^15^

### Inter-rater reliability and blinding

Percentage agreement was 99.6% (Cohen’s k=0.97) for SPCs, 98.5% (Cohen’s k=0.94) for time series charts, 89.0% (Cohen’s k=0.61) for time series and between group charts, and 89.9% (Cohen’s k=0.80) for quality and safety charts. In no cases was a rater ‘de-blinded’ such that they could discern whether a board paper arose before or after the salient intervention period. There were 12 images referred to the chief project investor because it was unclear whether they were charts (eg, the resolution may have been too poor to tell) and agreement on the appropriate decision was reached in all cases.

### Chart characteristics for all charts in intervention and control hospitals

There were 6318 charts identified. However, 31 were either educational SPCs with example data, illustrative data not about the hospital, or they were icons without any data. These charts were removed from the analyses. After excluding these charts, 6287 charts were retained for analyses (see table 2). Nearly one-half of charts (3003/6287, 48%) were quality and safety charts. Time series charts were more common (4741/6287, 75%) than between group charts (640/6287, 10%) and 906/6287 (14%) charts were comprised of both time series and between group presentations (combined). Of all 6287 charts, 449 (7%) were SPCs. Of the 449 SPCs, 63/449 (14%) had a summary icon displayed on them, and the control limits were labelled for 342/449 (76%) of the SPCs. For most charts with labelled limits (191/342, 56%), the label was UCL (‘upper confidence limit’) or LCL (‘lower confidence limit’) rather than specifying where the limit was set (see online supplemental file 6 for further description of the SPCs).

**Table 2:** Chart characteristics (all charts)

Type of chart	All charts (n=6287) n (%)
Quality and safety chart	3003 (47.7)
Time series, between group or both	6287 (100)
Time series only	4741 (75.4)
Between group only	640 (10.2)
Time series and between group	906 (14.4)

Further details available in online supplemental file 6.

### Effects of training intervention (intervention versus control hospitals)

#### All charts

**Figure 5:**
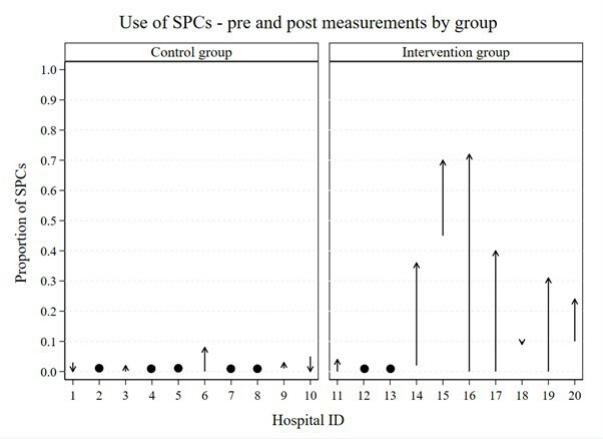
Use of SPCs—premeasurements and postmeasurements by group. SPC, statistical process control chart.

The raw numbers and proportions of SPCs used by group (control and intervention), hospital and time-period (preintervention and postintervention) for all charts are shown in table 3 and figure 5. On average in the control group, there was very little change in use of SPCs from before (9/1585, 0.6%) to after (23/1900, 1.2%) the intervention period (average difference 0%, 95% CI −2% to 2%). In the training intervention group, use of SPCs increased from 89/1235 (7%) to 328/1567 (21%), and the average difference was 22% (95% CI 2% to 42%). On average, the absolute difference in use of SPCs was 17% (95% CI 6% to 27%) higher in the intervention group compared with the control group. Use of SPCs in the postintervention period was nine times higher (95% CI 3 to 32) in the intervention group compared with the control group, adjusting for the preintervention (baseline) proportion of SPCs.

**Table 3:** SPC usage by group, hospital and period (all charts)

Control group				Intervention group			
Hospital	Preintervention	Postintervention	Post-Pre	Hospital	Preintervention	Postintervention	Post-Pre
	SPC/chart (%)	SPC/chart (%)	% difference		SPC/chart (%)	SPC/chart (%)	% difference
1	2/62 (3)	0/81 (0)	−3	11	1/206 (0)	9/225 (4)	4
2	0/87 (0)	0/127 (0)	0	12	0/149 (0)	0/131 (0)	0
3	0/13 (0)	2/119 (2)	2	13	0/123 (0)	0/84 (0)	0
4	0/643 (0)	0/687 (0)	0	14	3/140 (2)	91/256 (36)	34
5	0/158 (0)	0/170 (0)	0	15	52/116 (45)	47/67 (70)	25
6	0/101 (0)	15/179 (8)	8	16	0/70 (0)	58/81 (72)	72
7	0/157 (0)	0/151 (0)	0	17	0/18 (0)	27/67 (40)	40
8	0/104 (0)	0/101 (0)	0	18	18/176 (10)	42/457 (9)	−1
9	2/153 (1)	6/200 (3)	2	19	0/89 (0)	27/86 (31)	31
10	5/107 (5)	0/85 (0)	−5	20	15/148 (10)	27/113 (24)	14
Total	9/1585 (0.6)	23/1900 (1.2)	0.6	Total	89/1235 (7)	328/1567 (21)	14
Average difference in control group (95% CI)			0 (−2 to 2)	Average difference in intervention group (95% CI)			22 (2 to 42)
				Average difference between intervention and control group* (95% CI)			17 (6 to 27)
				Average relative change between intervention and control group† (95% CI)			9 (3 to 32)

For each hospital in preintervention and postintervention periods, the number of SPCs, the number of all charts and percentage of SPCs out of all charts are reported.

*T-test comparing average difference in proportions between intervention and control group. Percentage difference and 95% CI are reported.

†Zero-inflated negative Binomial regression models. Outcome is number of SPCs in postintervention period, adjusting for preintervention proportion of SPCs. Exposure is all charts. Risk ratios and 95% CI are reported.

SPC, statistical process control chart.

#### Subset of quality and safety charts only

As planned, we carried out an analysis restricted to quality and safety charts. The raw number and proportions of SPCs used by group (control, intervention), hospital, and time-period (preintervention versus postintervention) for quality and safety charts are shown in table 4. In the control group, there was very little change in use of SPCs before (7/657, 1%) to after (12/741, 2%) the training intervention period (average difference 0%, 95% CI −3% to 4%). In the training intervention group, use of SPCs was 71/684 (10%) before and 213/921 (23%) after the training, and the average difference was 21% (95% CI 0% to 42%). On average, the difference in use of SPCs was 18% (95% CI 7% to 29%) higher in the intervention group compared with the control group. In model-based analyses, use of SPCs in the postintervention period was nine times higher (95% CI 2 to 41) in the intervention group compared with the control group.

**Table 4:** SPC usage by group, hospital and period (planned subgroup analysis—quality and safety charts only)

Control group				Intervention group			
Hospital	Preintervention	Postintervention	Post-Pre	Hospital	Preintervention	Postintervention	Post-Pre
	SPC/chart (%)	SPC/chart (%)	% difference		SPC/chart (%)	SPC/chart (%)	% difference
1	2/23 (9)	0/16 (0)	-9	11	1/130 (0)	3/125 (2)	2
2	0/56 (0)	0/95 (0)	0	12	0/87 (0)	0/71 (0)	0
3	0/13 (0)	2/26 (8)	8	13	0/38 (0)	0/29 (0)	0
4	0/189 (0)	0/198 (0)	0	14	3/95 (3)	49/152 (32)	29
5	0/80 (0)	0/86 (0)	0	15	37/70 (53)	33/47 (70)	17
6	0/50 (0)	9/98 (9)	9	16	0/47 (0)	26/41 (63)	63
7	0/86 (0)	0/86 (0)	0	17	0/11 (0)	25/48 (52)	52
8	0/60 (0)	0/52 (0)	0	18	16/74 (22)	35/285 (12)	−10
9	0/40 (0)	1/44 (2)	2	19	0/50 (0)	19/46 (41)	41
10	5/60 (8)	0/40 (0)	−8	20	14/82 (17)	23/77 (30)	13
Total	7/657 (1)	12/741 (2)	1	Total	71/684 (10)	213/921 (23)	13
Average difference in control group (95% CI)			0 (−3 to 4)	Average difference in intervention group (95% CI)			21 (0 to 42)
				Average difference between intervention and control group* (95% CI)			18 (7 to 29)
				Average relative change between intervention and control group† (95% CI)			9 (2 to 41)

For each hospital in preintervention and postintervention periods, the number of SPCs, the number of all charts and percentage of SPCs out of all charts are reported. Subgroup analysis safety and quality charts only.

*T-test comparing average difference in proportions between intervention and control group. Percentage difference and 95% CI are reported.

†Zero-inflated negative Binomial regression models. Outcome is number of SPCs in postintervention period, adjusting for preintervention proportion of SPCs. Exposure is all charts. Risk ratios and 95% CI are reported. Subgroup analysis safety and quality charts only.

SPC, statistical process control.

#### Subset of time series charts

Further analyses regarding changing the exposures to time series charts and between group charts are reported in online supplemental file 7, Tables S7-2 and S7-3. For the model with the time series chart exposure, the results were broadly similar to the main analysis.

#### Subset of time series and between group charts

For the model with the times series and between group exposure, the average difference in use of SPCs was 10% (95% CI 0% to 20%) higher in the intervention group compared with the control group. The zero-inflated negative binomial model did not converge for these data, possibly due to the high number of zero cells in the outcome (37/40 observations).

#### Thematic analysis of qualitative data

Written responses from the feedback forms were available for 7 out of 10 hospitals in the training intervention sample, including two hospitals that increased the SPCs in board papers by less than 10%. Most comments consisted of a few words or one sentence. The main themes relating to responses to the question about what went well were the general format, content and delivery of the training (n=21/66), such as ‘Topic relevant and timely’; practical and personal examples that use own hospitals’ data (n=19/66), such as ‘trust (hospital) data brought it alive’; conversation, discussion and interaction (n=10/66), such as ‘interactive opportunity to discuss examples’; formatting, use and insights (n=10/66), such as ‘good explanation of SPC rules’ and other general comments (n=6/66).

The question about what could have been done differently during the training elicited fewer responses overall (n=32) than did the question about what went well (n=66); this was true across hospitals, including those that changed their use of SPCs both more and less than 10%. The main themes relating to what could have been done differently were the session format (n=15/32), such as ‘more time for discussion’ and ‘break out into groups’; no suggestions for doing anything differently (5/32); the training content (4/32), such as having a ‘technical supplement’ and ‘more on the calculation of control limits’ and requests for more examples using own hospital data (3/32), providing handouts (3/32) and other (2/32).

Most participants mentioned awareness of SPCs themselves as a key takeaway (n=29/70). Others commented on the general use of SPCs (n=23/70), such as trend lines, tools and templates, and understanding ‘how poor presentation can lead to poor decisions’. Several participants commented that the training changed how they interpret data (n=6/70), intend to report data (6/70) or generally think about data and reporting (4/70). The other comments (n=2/70) were about encouraging others and timelines for implementation.

Finally, when asked for any other comments, most participants made generally positive comments on the training (25/26). Only one (1/26) participant suggested that ‘next steps are important’, which may reference the need to consider implementation steps in training.

## Discussion

### Summary of main results

This study investigated whether an educational training intervention increased the use of SPCs in NHS hospitals. We studied the board papers of 10 hospitals that received the training before and after the intervention, along with those from 10 control hospitals that did not receive training over the same time-period. The results showed that most hospitals increased the proportion of SPCs in their board papers after the training intervention, while there was almost no change in the proportion of SPCs among the controls. In model-based analyses, trained hospitals increased their uptake nine-fold relative to controls. The intervention consisted of a day of training for quality improvement staff and 90 min for board members. As this is not a highly intensive intervention, it should be scalable across most contexts.

### Interpretation of main results

#### Interpretation with reference to prior literature

These results are important for several reasons. First, many hospitals do not depict statistical variation in the documents used to inform decision-making about process variation.^7^ Second, the use of SPCs enables management’s recommendations to align with statistical findings.^4^ A recent trial in France found that surgical departments using SPCs had better patient outcomes than controls. Notably, the French intervention appeared more intensive than the training intervention that we evaluated. It provided departments with SPCs from publicly available data, encouraged structured meetings and supplied logbooks for completion. These activities were all in addition to 3 days of training.^6^ Our results suggest that a simpler approach can effect change in the prevalence of charts in board papers, although it is a matter of opinion as to whether the change in the hospitals that improved was sufficient to influence improvements in processes and outcomes. Evidence on generalisable mechanisms linking the appearance of charts to quality improvement would more fully inform such opinions, such as perceptions of decisions taken based on the charts and hospital culture.

#### Interpretation of heterogeneity of the results

Improvement was not uniform across intervention hospitals. The qualitative data do not explain why some hospitals improved but not others, as nearly all respondents reported positive perceptions of the training—including in hospitals that did not change their use of SPCs in board papers. However, these positive responses may have been shaped by social desirability bias.^19^ Moreover, some respondents requested more information, including a technical supplement and more on calculating control limits, suggesting that not all training needs had been fulfilled and further sessions or re-engagement may be required.

#### Interpretation of proportional changes

There are several mechanisms by which the proportional changes in this study could be brought about. As intended, many charts that were previously not produced as SPCs could be transformed into SPCs. However, the total number of charts in the denominator could have decreased because of the intervention, thereby exaggerating improvement in the proportion of hospitals using SPCs (see detailed discussion in online supplemental file 8). Note that this mechanism is possible even in a randomised trial, as the intervention could have prompted changes in the number of charts presented to boards. On balance, we interpret our results as supporting the increased adoption of SPCs while acknowledging the alternative mechanisms. We also note that there is no agreed proportion of SPCs in board papers that would indicate sufficient usage after training, and the need for SPCs could vary by context as topics of concern may change over time.

10.1136/bmjqs-2021-013514.supp8 Supplementary data

### Issues related to the presentation of SPCs in board papers

The presentation of SPCs could be further improved. Nearly half of SPCs did not state where the control limit had been set, either not mentioning the limit or simply recording ‘UCL’ and ‘LCL’ without specifying the limit (eg, three SD). Without labels on limits, the degree of uncertainty that they represent is unclear. We did not compare the labelling and limits of intervention and control hospitals due to the small number of identified SPCs.

### Issues related to the implementation of SPCs in hospitals

The use of SPCs takes place within broader organisational contexts. It is possible that SPCs are not included in board papers but are used elsewhere—such as in quality and safety subcommittees. We believe this is unlikely given the explicit quality assurance function of hospital boards. Training alone may be insufficient to encourage adoption of SPCs if the organisational context is not supportive. Importantly, SPC usage is not a sufficient condition for improvement, just as checklists cannot, by themselves, effect safe practice.^20^ There must be a supportive implementation context: a team of analysts to create the charts, board members who view and interpret charts, managers who discuss and act on the information presented in the chart and staff at the front line. SPCs are but one element in a chain of events influencing the safety and quality of patient care.

### Limitations

#### Limitations of our study

Our research design does not fully permit a causal interpretation of the results. However, the use of contemporaneous controls showed that our results are not likely due to a ‘rising tide’ of greater use of SPCs among all NHS hospitals.^21^ Although control hospitals were selected to be as similar as possible to intervention hospitals, clear differences were observed at baseline, including in use of SPCs (Hospitals 15, 18, 20). We adjusted for observed differences between hospitals and the before and after design allows us to adjust for differences in baseline rates of the outcome variable (use of SPCs). However, especially given baseline differences, we must suspect unobserved confounders; for example, the intervention hospitals might have been more motivated to change in response to the training.

#### Limitations of research in the area

Future research should consider an investigation that randomly assigns hospitals to training interventions to balance these factors between groups. Other investigations might also research effects for other forms of hospitals, such as mental health or community care hospitals, to explore generalisability. Studies could explore which aspects of the training are effective, such as the personalisation element, trainers themselves and trainees’ understanding and confidence.^20^ Importantly, the causal chain linking the prevalence of charts in board papers to patient outcomes should be evidenced, including by qualitatively understanding decision-making related to patient care.

#### Limitations of qualitative research

A limitation of our qualitative data is that it came from feedback solicited only shortly after the intervention, which restricts the investigation of mechanisms like confidence in the longer term.

## Conclusion

Certainly, not all the charts within board papers could or should be SPCs. SPCs are not a panacea for understanding data related to all quality improvement issues. However, the high proportion charts with time series information in the board papers (90%), combined with lack of use of SPCs, suggests substantial scope to better visualise chance variation in the data presented to decision-makers. Our results suggest that educational training initiatives may bolster progress towards this aim.

## Data Availability

Data are available on reasonable request.

## References

[1] Dekker S . Foundations of safety science : a century of understanding accidents and disasters. Boca Raton, Florida: Taylor & Francis Group, CRC Press, 2019.

[2] Shewhart WA . Statistical methods from the viewpoint of quality control. Dover 1986 edition with Foreword by W. Edwards Deming, originally published 1939. Mineola, N.Y.: Dover Publications, 1986.

[3] Mohammed MA , Cheng KK , Rouse A , et al . Bristol, Shipman, and clinical governance: Shewhart's forgotten lessons. Lancet 2001;357:463–7. 10.1016/S0140-6736(00)04019-8 11273083

[4] Schmidtke KA , Watson DG , Vlaev I . The use of control charts by laypeople and hospital decision-makers for guiding decision making. Q J Exp Psychol 2017;70:1114–28. 10.1080/17470218.2016.1172096 27028900

[5] Tversky A , Kahneman D . Judgment under uncertainty: Heuristics and biases. Science 1974;185:1124–31. 10.1126/science.185.4157.1124 17835457

[6] Duclos A , Chollet F , Pascal L , et al . Effect of monitoring surgical outcomes using control charts to reduce major adverse events in patients: cluster randomised trial. BMJ 2020;371:m3840. 10.1136/bmj.m3840 33148601 PMC7610189

[7] Bird P . Understanding the right and wrong time for intervention. Health Serv J 2017 https://www.hsj.co.uk/quality-and-performance/understanding-the-right-and-wrong-time-for-intervention/7015608.article

[8] NHS England . Making data count. Available: https://www.england.nhs.uk/publication/making-data-count/ [Accessed 29 Jan 2021].

[9] Juran JM . The quality trilogy. A universal approach management fot quality. Qual Prog 1986;19:19–24.

[10] Kudrna L , Bird P , Hemming K , et al . Protocol for a retrospective evaluation of an intervention based on training sessions to increase the use of control charts in the NHS 2021. 10.17605/OSF.IO/2ZXA9 PMC9887349 35750493

[11] Ogrinc G , Davies L , Goodman D , et al . Squire 2.0 (standards for quality improvement reporting excellence): revised publication guidelines from a detailed consensus process. BMJ Qual Saf 2016;25:986–92. 10.1136/bmjqs-2015-004411 PMC5256233 26369893

[12] Hoffmann TC , Glasziou PP , Boutron I , et al . Better reporting of interventions: template for intervention description and replication (TIDieR) checklist and guide. BMJ 2014;348:g1687. 10.1136/bmj.g1687 24609605

[13] Lilford RJ , Chilton PJ , Hemming K , et al . Evaluating policy and service interventions: framework to guide selection and interpretation of study end points. BMJ 2010;341:c4413–20. 10.1136/bmj.c4413 20802000

[14] Frison L , Pocock SJ . Repeated measures in clinical trials: analysis using mean summary statistics and its implications for design. Stat Med 1992;11:1685–704. 10.1002/sim.4780111304 1485053

[15] NHS Digital . Nhs trust peer finder tool. Available: https://digital.nhs.uk/data-and-information/data-tools-and-services/data-services/innovative-uses-of-data/multi-dataset-analysis/nhs-trust-peer-finder-tool [Accessed 29 Jan 2021].

[16] Department for Communities and Local Government . The English indices of deprivation, 2010. Available: https://assets.publishing.service.gov.uk/government/uploads/system/uploads/attachment_data/file/6320/1870718.pdf [Accessed 8 Nov 2021].

[17] Schmidtke KA , Poots AJ , Carpio J , et al . Considering chance in quality and safety performance measures: an analysis of performance reports by boards in English NHS trusts. BMJ Qual Saf 2017;26:61–9. 10.1136/bmjqs-2015-004967 27034337

[18] Braun V , Clarke V . What can "thematic analysis" offer health and wellbeing researchers? Int J Qual Stud Health Well-being 2014;9:26152. 10.3402/qhw.v9.26152 25326092 PMC4201665

[19] Groves R JFF , Couper M , et al . Survey methodology. Hoboken, N.J.: John Wiley & Sons, 2009.

[20] Dixon-Woods M , Bosk CL , Aveling EL , et al . Explaining Michigan: developing an ex post theory of a quality improvement program. Milbank Q 2011;89:167–205. 10.1111/j.1468-0009.2011.00625.x 21676020 PMC3142336

[21] Chen Y-F , Hemming K , Stevens AJ , et al . Secular trends and evaluation of complex interventions: the rising tide phenomenon. BMJ Qual Saf 2016;25:303–10. 10.1136/bmjqs-2015-004372 PMC4853562 26442789

