# Supplementary material for: Retrospective evaluation of an intervention based on training sessions to increase the use of control charts in hospitals
Source: BMJ Qual Saf. 2022 Jun 24;32(2):100–8. doi: 10.1136/bmjqs-2021-013514 (PMC9887349; doi:10.1136/bmjqs-2021-013514)
Supplement: Supplementary data [file bmjqs-2021-013514supp001.pdf]

Supplementary 1 - Protocol

Protocol for a retrospective evaluation of an intervention based on training sessions to increase the use of control charts in the NHS

Supplementary 1 - Protocol

Abstract

Background. Hospital board members use data to inform their decision-making. The way that these data are presented can impact whether hospital board members choose to intervene. Existing research shows that the process limits on statistical process control (SPC) charts improve the ability of hospital decision-makers to align their investigative recommendations with statistical findings. Yet SPCs are not widely used within the UK National Health Services (NHS). An educational training initiative called 'Making Data Count' was established by NHS Improvement/England (NHS-I/E) to improve the uptake of SPC charts in NHS institutions. The present research will evaluate the impact of NHS-I/E training sessions on SPC chart usage.

Methods. A controlled before and after design. SPC chart usage will be examined in a sample of 40 board papers across 20 trusts. The sample will include 20 board papers across ten trusts that have completed the training intervention (ten pre and ten post intervention), and 20 board papers across ten different trusts that have not completed the training intervention that will be external controls. These external controls will be matched according to trust characteristics and time period. Poisson regression will be used to compare rates of control chart usage pre and post intervention, and between the intervention and control groups, using a difference in difference approach. Qualitative thematic analysis of feedback forms will be conducted.

Discussion. The present research will evaluate the impact of NHS-I/E training sessions on the use of SPC charts by examining whether SPC charts appear in NHS trust board papers before and after trainings. The results will contribute to our understanding of whether and why educational initiatives are effective in changing how data are used within healthcare settings.

Supplementary 1 - Protocol

Background

Problem description

Consider the following scenario: you are on the board of an NHS trust and have just received new data showing that average waiting times increased last week. Although you have not yet exceeded the national target for waiting times, you are inching ever-closer. You are uneasy. You do not want to be in breach of the target, but you are not sure that the increase from last week is meaningful enough to take any action. What steps would you take in order to decide whether the increase is meaningful?

While there are many aspects of this scenario that you could investigate, such as how far you are from the target and whether there have been any clinic cancellations recently, an important consideration is whether last week's increase is due to chance. In other words, is the variation within the bounds of what would be expected due to random fluctuations in the data that naturally occur over time? Despite the importance of this question, the data presented to boards do not always contain sufficient information for board members to consider how chance influences key indicators over time (Schmidtke et al., 2017). Omitting the role of chance could lead to sub-optimal decision-making and, consequently, inefficient allocation of resources. Adverse consequences might manifest through unnecessary intervention for a metric that has been incorrectly interpreted as deteriorating performance when it is in fact expected (or 'common-cause') variation.

Available knowledge

In the United Kingdom, the term 'trusts' refers to organisations within the National Health Service (NHS) that provide healthcare services. These trusts have boards that are comprised of executive and non-executive members who collaboratively review documents and make decisions about ongoing performance. The documents associated with these meetings are published as publicly available 'board papers', which contain text and charts. Some of the charts are statistical process control (SPC) charts, whereas others are not SPC charts.

Historically, SPCs charts were first developed for the manufacturing industry and their use in the health sector is widely recommended (Mohammed, Cheng, Rouse & Marshall, 2001).

SPC charts can help decision-makers consider the role of chance by displaying 'process limits' that depict statistically informed thresholds, such as how far away a data point is from the mean. Examples of charts without and with process limits are shown in Figures 1 and 2, respectively. These are fictitious and stylised charts displaying 'diagnostic assessment compliance' rates for a disease from April 2016 to October 2017.

Figure 1: Run chart showing monthly changes in diagnostic assessment compliance – without process limits (stylised example)

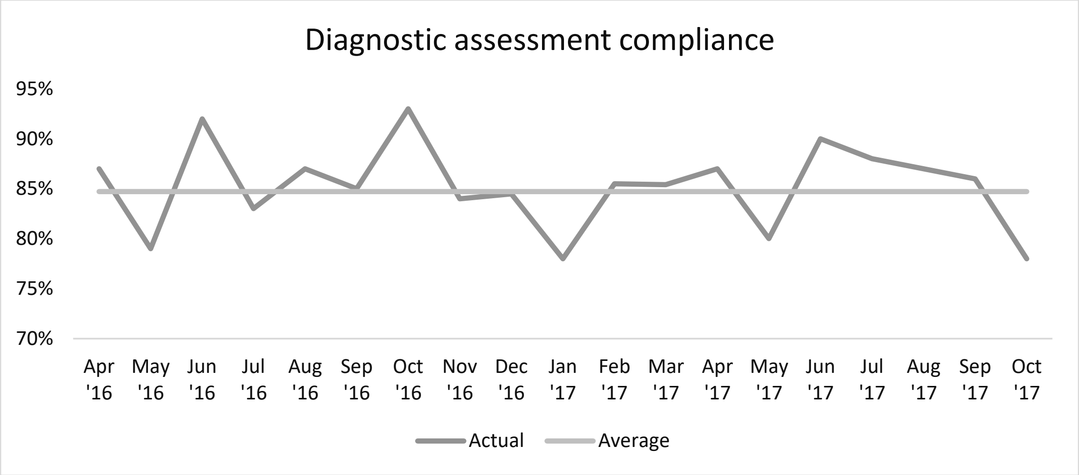

Figure 2: Statistical process control chart showing monthly changes in diagnostic assessment compliance – with process limits (stylised example)

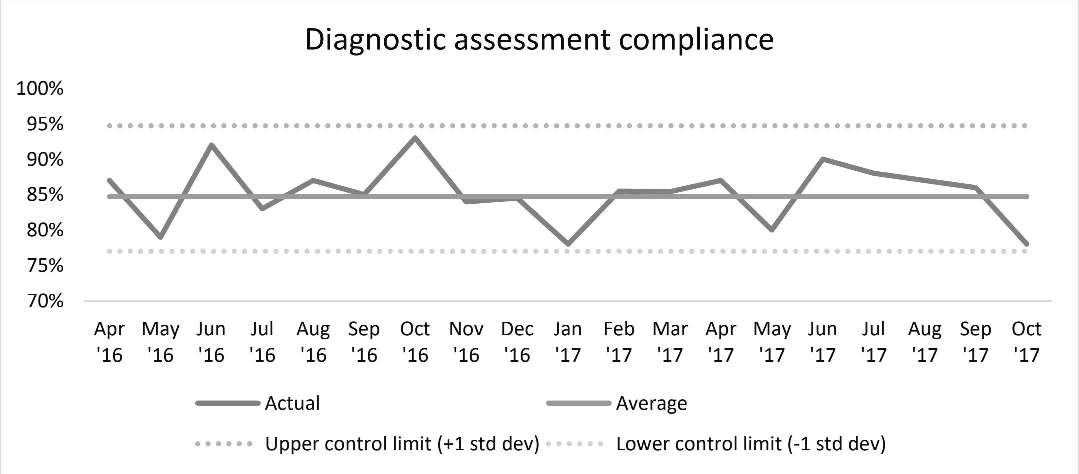

In Figure 1, where the data do not have process limits, it is difficult to ascertain whether monthly compliance rates that are above and/or below the mean are departures from natural

variation over time. In Figure 2, with process limits, it is possible to see that the variations can be predicted by chance, at least within the specified process limits displayed as dashed lines. Further examples of SPC charts are contained in Appendices A and B (available here, https://bit.ly/3j0N4Iu), which are discussed in more detail in the Methods.

Despite recommendations to use SPC charts to monitor performance measures, SPC charts are still sparsely used in healthcare (Schmidtke et al., 2017). Other data presentation methods that do not include the role of chance are prevalent, such as R-A-G charts. R-A-G charts are typically tables of data colour coded to indicate whether data fail to meet a specific target (*r*ed), are in danger of not meeting that target (*a*mber), or are achieving and meeting that target (*g*reen). These targets are seldom informed[1] by the data, and, therefore, are not always well suited to guide quality improvement (Anhøj & Hellesøe, 2017). In contrast, the process limits in SPC charts are data-driven, such as two or three sigma or standard deviations above or below the mean (Wheeler, 2013).

SPC charts can improve people's abilities to identify outliers and align their investigative recommendations with statistical findings (Schmidtke, Watson & Vlaev, 2016). One of the reasons that incorporating process limits into run charts assists with interpreting the data is that they make sample size more salient, thus mitigating a cognitive bias called 'base-rate neglect' (Tversky & Kahneman, 1974; Schmidtke et al, 2017). However, whether SPC charts improve decision-making through automatic or meaningfully reflective cognitive processes may depend on various factors, including what other information is presented in the chart. One factor may be whether the chart includes a label describing where the process limits are set, such as the use of one standard deviation in Figure 2. Labelling enables decision-makers to more accurately understand what it means if data are outside the control limits. Without these limits, decision-makers choices may still align with statistical recommendations, but only in an automatic cognitive capacity brought about by what the chart dictates as a statistical aberration using the r-a-g method.[2]

[1] The thresholds at which the RAG limits are set are sometimes user-defined. For example, if national target is to be above 90%, one Trust may define Amber as being performance below 94% - another may decide on 92%.
[2] These are not the only criteria that may influence whether decision-makers engage in reflective and/or automatic thinking. For example, decision-makers also need to have sufficient skills and knowledge to interpret the process data being modelled within the chart, in addition to the opportunity to so (Michie et al, 2011)

Of course, many other factors can influence whether board members can use control charts effectively. In order for any behaviour to occur, people must possess the relevant capabilities (psychological, physical), opportunities (social, physical) and motivations (reflective, automatic; Michie, et al. 2011). For example, board members need to know how to interpret the information presented in the control chart (a capability factor), have the motivation to engage with the data at a deeper level than they would with target-focused evaluations (a motivational factor), and have access to satisfactorily constructed SPC charts in their board papers (an opportunity factor). The present study focuses on the capability and opportunity factors: explaining the use of SPC charts to board members and increasing the number of control charts present in NHS board papers, respectively.

There are a large number of studies about specific quality improvement methodologies such as Lean, Six-sigma and Plan-Do-Action cycles that may use SPC methodology as part of the improvement process (Deblois & Lepanto, 2016). We are, however, not interested in the use of SPC methods as part and parcel of an intervention to improve a given process. Rather, we are interested in SPC methods being used in routine surveillance to identify processes to be improved. To understand if any similar studies had already been conducted, we therefore carried out a systematic literature search for methods to improve the use of SPC for routine surveillance. Our search strategy is laid out in Figure 3 and discussed in detail in Appendix C (available here: https://bit.ly/3j0N4Iu). We found no papers that replicated our study, and we can assert that this is the first study to examine the effectiveness of an intervention to increase the use of SPC charts across a range of routine monitoring programs at the institutional level.

Figure 3: Results of systematic review seeking studies on training interventions to increase the use of SPC charts for routine monitoring within institutions

Supplementary 1 - Protocol

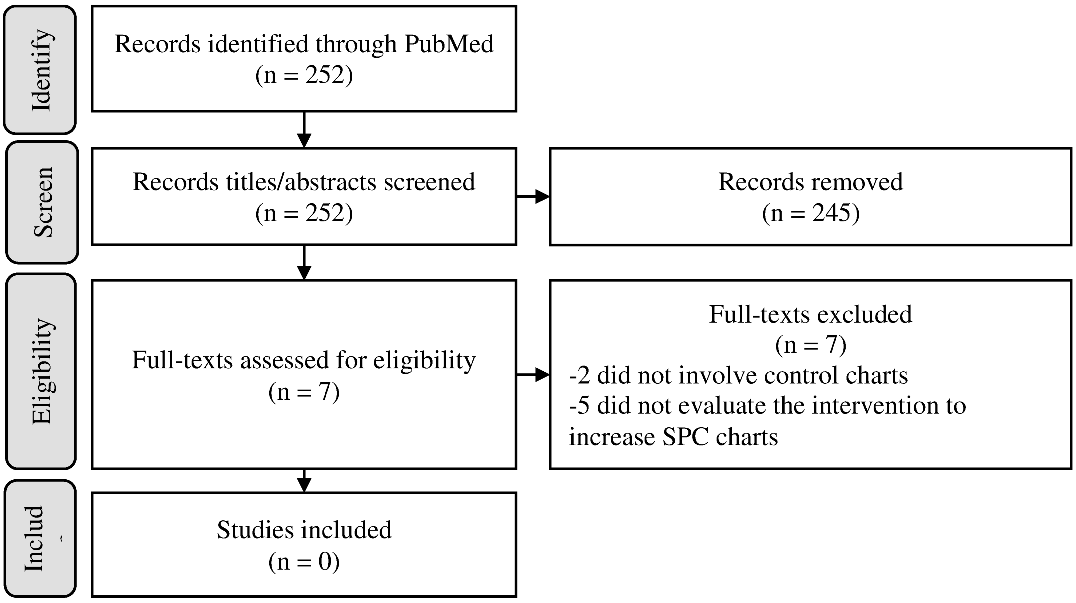

Rationale

A previous review of 30 randomly selected English acute care trusts' board papers found that 13 (43%) lacked even a single chart depicting the role of chance (Schmidtke et al., 2017). This problem was summarised in a popular health management magazine (Bird, 2017), and underpinned the NHS Improvement/England (NHS I/E) (2019) initiative called 'Making Data Count' to increase the use of SPC charts in NHS institutions. This initiative has involved a set of educational resources along with a series of training sessions on the implementation and use of SPCs. The effectiveness of this training initiative has not yet been captured in a scientifically rigorous manner.

Specific aims

We set out to evaluate the effects of the Making Data Count training sessions on the use of SPC charts by NHS trusts, and to assess how the trainees perceived the training sessions.

Methods

Supplementary 1 - Protocol

Our objectives were to identify if the training resulted in an increase in the proportion of SPC charts in board papers, and to thematically analyse participants' reactions to the training sessions. This research protocol was prepared according to SQUIRE guidelines (Ogrinc, Davies, Goodman, Batalden, Davidoff & Stevens, 2016).

Context

The Making Data Count training sessions were delivered to NHS trust boards and to teams of hospital analysts by NHS Improvement from November 2017 onwards. NHS Improvement uses social media, email and word of mouth to invite trusts to participate. Thus, there is self-selection into the training sessions, and the approach to recruitment into the training sessions is effectively snowball sampling. All trusts that received a training session that we will investigate are based in England.

Intervention

This intervention is described here according to TIDieR guidelines (Hoffman et al, 2014). Our completed TIDier checklist is in Appendix D (https://bit.ly/3j0N4Iu). The brief name of the intervention is 'Making Data Count SPC training sessions'. The training sessions are conducted to improve knowledge about SPC charts and increase their uptake. Two examples of PowerPoints slides used in the training sessions are shown in Appendices A and B (https://bit.ly/3j0N4Iu). The two Making Data Count guidebooks that supplement the training are available online (NHS Improvement, 2019). The training sessions cover the strengths and weaknesses of presenting data in different ways, and include background on what SPC charts are, when and how to use them, why they should be used, and how they can improve decision-making. Topics include identifying trends (e.g. seven points in one direction), special versus common cause variation, and summarising data using icons (see Appendix B, Slide 47, https://bit.ly/3j0N4Iu). The limitations of *R-A-G* systems are discussed, and, importantly, each training is personalised: trusts' data from their board papers are presented using SPCs in order to demonstrate the value of using SPCs.

The Making Data Count training sessions are delivered at each trust to up to two groups of people separately, as mentioned above: board members and analysts. The training sessions

Kudrna L, *et al. BMJ Qual Saf* 2023; 32:100–108. doi: 10.1136/bmjqs-2021-013514

for board members are delivered over about 90 minutes, while training sessions for analysts are delivered over one working day. Training sessions are delivered by two experienced trainers from NHS Improvement with higher educational backgrounds in statistics and work experience in data analytics. One trainer visits each trust to deliver the training face-to-face to board members and, separately, to teams of analysts. Board and analyst trainings are not necessarily given on the same day and can be separated by around a month.

Study of the intervention (evaluation design)

Design

This study will conduct a quantitative and qualitative evaluation of the training sessions. The quantitative evaluation will be a controlled before and after design that uses data from ten acute care trusts that received the training, as well as ten different acute care trusts that will be external matched controls. Board papers from before and after the training dates will be selected. The qualitative evaluation will thematically analyse responses to feedback forms from some of the trusts. Overall, the study design is pragmatic and determined by resource capacity to find and extract data from the board meeting papers.

Selection of acute care trusts

Due to resource constraints, we will be unable to examine board papers in all trusts that received a training from the start of the intervention period in March 2018. Instead, we will focus on the acute care trusts from the first year of trainings through March 2019. We will select ten trusts that received the training during different months in order to maximise temporal heterogeneity. These ten trusts are the training intervention sample.

We will also identify ten acute trusts that have not received the training intervention to be external matched controls. The ten trusts in the intervention group will be matched to ten other trusts using the NHS Digital (2020) Peer Finder tool. This tool identifies trust peers based on variables such as attendances, deprivation, and patient profiles, and proposes ten peers with the smallest Euclidean distance to the selected trust. We will view the ten closest matches using the default tool weightings. From these ten closely matched trusts, we will select (without replacement) trusts that meet the following criteria (in order):

• did not receive a training intervention,
• similar number of attendances,
• similar degree of specialisation, and
• similar level of deprivation.

Occasionally, as tie breakers, other factors such as the number of FTE (full time equivalent) staff, urban location, and foundation status may be used as additional criteria.

Selection of board papers from acute care trusts

For the intervention group, we will identify board papers published in the month before the intervention was delivered (pre intervention observation) and approximately six months after delivery in each trust (post intervention observation). Boards do not publish their papers every month. In some cases, therefore, it is not possible to sample board papers exactly immediately prior to the training or six months immediately after the training. When it is not possible to select a board paper from the assigned month pre training, the first board paper published at least one month before intervention delivery will be selected; when it is not possible to select a board paper from the assigned month post-training, the first board paper published at least six months after the intervention delivery will be selected. This approach is shown in Figure 4, which represents the realised design in the intervention sample accounting for the fact that not all trusts have board papers available in the first month before the intervention or six months post intervention roll-out.

Supplementary 1 - Protocol

Figure 4: Selected board papers from for pre intervention and post intervention periods, and month of training intervention, for ten acute trusts that received ‘Making Data Count’ training sessions

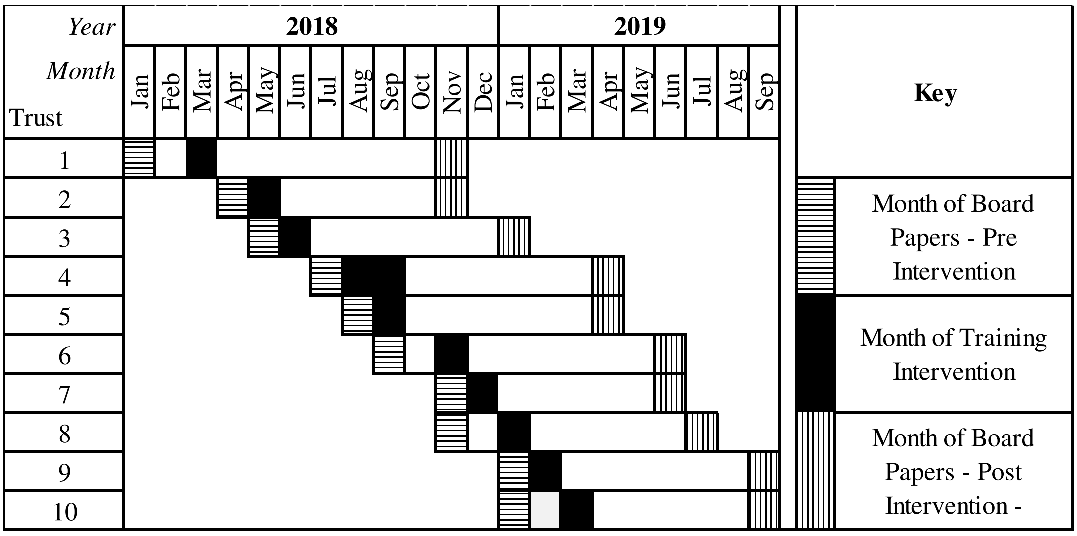

Notes: The black boxes show the month of the training intervention. The ten cells with horizontal lines are those months where we will sample the pre intervention board papers from before the training. The ten cells with vertical lines are those months where we will sample the post-intervention board papers from after the training, which occur at least six months after the training. Trust 4 received trainings in August and September.

For the external matched control group (not shown in Figure 4), we will identify board papers published in the months closest to the pre and post intervention observations from the matched trust in the intervention group. Overall, this equates to 20 different intervention and matched control trusts in total, each contributing two papers, for a total sample of 40 board papers.

Quantitative measures

We will create three quantitative measures from data in the board papers. The main outcome measure will be the proportion of charts that were SPC charts out of all charts presented. The other two outcomes will be the proportion of charts that were SPC charts out of all time series charts, and the proportion of charts that were SPC charts out of all time series *and* between group charts.

The rationale behind selecting the first outcome is that increasing the use of SPC charts is a main focus of the training intervention, and it can be created from information that is publicly available in board papers. This outcome may be considered a broad level at which the effects of training on control chart usage may be evidenced. Not all charts, however, can be easily transformed into SPC charts. The rationale behind selecting the other two outcomes is that time series and between group charts can be more directly transformed into SPC charts than can other types of charts, such as pie charts. Time series and between groups charts are, therefore, the types of charts that we most expect the training sessions to influence. We focus on time series charts separately because time sequences "in order" were the types of charts that Shewart's original SPC methodology encouraged (Shewhart, 1939/1986, p.12). Some additional descriptive information about all of the charts in the board papers, as well as about the SPC charts specifically, will be recorded (this is discussed in the section further below on 'data extraction').

Data extraction from board papers

We will extract information from the board papers to populate the quantitative outcomes discussed above: number of SPC charts, total number of charts, number of time series charts, and number of between group charts. We will also extract additional information about the charts to illustrate the specific contexts where the training may be effective. The charts will be classified as 'quality and safety' charts or not, following Schmidtke et al (2017), which may be interpreted in various ways. One definition of quality and safety is whether care "conforms to established treatment goals and care processes" (quality) and "avoids injuries to patients" (safety), as discussed by the Institute of Medicine (2002, p.92). Guided by this definition, our approach will use multiple raters to assess whether a particular chart depicts quality and safety information.

Additional information about the nature and content of SPC charts identified will be recorded (see Appendix E, all Appendices here: https://bit.ly/3j0N4Iu): control limits (Appendix A, p. 38; Appendix E, item 10), recalculation of control limits (Appendix B, p. 44-46; Appendix E, item 11), run/trend points (Appendix A, p. 14; Appendix E, item 12), and whether there are comments about reasons for variation, or suggestions about intervening (Appendix B, p. 51; Appendix E, items 13-14). Whether the control limits are labelled is of interest because labels provide decision-makers with information that may engage reflective (vs. automatic)

cognitive processes. We will also assess whether r-a-g is still present in charts identified from the board papers (Appendix A, p. 4-7; Appendix E, item 1), and whether there are any icon summaries[3] (Appendix B, p. 47; Appendix E, item 8), which were also covered in training.

Blinding and agreement

One reviewer will download the board papers from the web and four independent reviewers will examine the board papers (reviewers R1, R2, R3, R4). Reviewers examining the board papers for the presence and nature of SPC charts will be blind as to whether the board paper is from the control or post intervention period. To ensure agreement and blinding, the below four steps will be taken. Steps one and three ensure agreement between raters, and steps two and four ensure blinding:

(1) *Identification and sampling of charts.* R1 will download the board papers. R1 and R2 will independently identify the total number of charts, and independently identify whether the chart is a quality and safety chart. R1 and R2 will discuss any disagreements to reach a consensus and inter-rater reliability will be calculated (prior to the consensus). Any unresolved disagreements will be referred to the chief project investigator.

(2) *Assessment of sample charts.* R1 screenshots the charts and removes any information about name of trust and/or date of board paper, randomises the order of trusts, and sends them to R3 and R4.

(3) *Examination of charts.* R3 and R4 will examine the charts and decide if the charts are SPC charts, time series charts, between group charts, or other types of charts. R3 and R4 will also give descriptions of the SPC charts according to the measures in Appendix E, described above.[4] Inter-rater reliability will be calculated and R3 and R4 will subsequently discuss to reach a consensus. Any unresolved disagreements will be referred to the chief project investigator.

(4) *De-blinding report.* R3 and R4 note if they have been de-blinded at any point.

[3] See Appendix E for example of SPC icon summary (here: https://bit.ly/3j0N4Iu).

Sample size calculation

We are looking for a substantial effect size because, in contrast to a clinical intervention which affects patients directly, this service intervention affects patients indirectly (Lilford et al., 2010). It is, therefore, doubtful whether service managers would want to replicate the training intervention unless they could achieve a substantial improvement in uptake. Our sample size is based on the detection of a 30 percentage point improvement in the proportion of charts that are SPC from 10% to 40% between pre and post intervention measures. Sample size is calculated with an alpha of 0.05 and power of 0.80. Due to the study design, adjustment for the correlation between pre and post intervention measures is made, which is estimated at r=0.90 (Frison & Pocock, 1992). A minimum of 16 hospitals with pre and post intervention measures is required.

Quantitative analysis

Information on the 20 hospitals will be summarised, including key characteristics used for the matching (attendances, specialisation, level of deprivation). Details about the identified SPC charts (control limits, recalculation of control limits, run/trend points, and whether there are comments about reasons for variation, or suggestions for intervention – see above and Appendix E) will be summarised as counts and proportional measures.

For each hospital, we will have information on the number of charts depicted as a SPC chart (the outcome), the total number of charts (an offset), the month of the observation, whether the observation was from the intervention or control group and whether the observation was from a pre or post intervention period. Other analyses will have an offset in two different ways; (1) time series charts only and (2) time series and between group charts.

A Poisson regression model will be fitted with an offset for the total number of charts and the outcome as the number of charts presented as an SPC control chart. We will adjust for group (intervention or control group), for period (pre or post intervention exposure) and an interaction between group and period (treatment effect) using a difference in difference approach. The offset in the model will be changed dependent on the outcome. Results will be reported on the rate ratio scale with 95% confidence intervals. Subgroup analysis will be conducted using quality and safety charts only.

Inter-rater reliability will be calculated using Kappa statistics and percentage agreement to quantity the level of agreement between reviewers for deciding on whether they were SPC charts, time series charts, between group charts, and quality and safety charts.

Qualitative evaluation

In addition to quantitative outcomes and analyses, we will conduct a qualitative evaluation to better understand barriers and facilitators to the uptake of SPC charts. Our qualitative process outcomes come from feedback forms that were filled out by training session participants during the board sessions (see Appendix F). These forms were designed by NHS-I/E and shared with the research team. We will analyse responses to the following four items:

1. What went well today?
2. What could have been done differently?
3. What are your key takeaways?
4. Any other comments about today.

We will conduct thematic analysis of written responses to these questions to identify barriers to and facilitators of using SPC charts (Braun & Clark, 2014).

Ethical considerations

This research has been approved by the University of Warwick Biomedical and Scientific Research Ethics committee (BSREC 116/18-19).

Discussion

Summary

Overall, this research will provide evidence about the impact of training sessions on the use of SPC charts among acute care hospital trusts in England. In addition, qualitative reactions to the training will also be provided. The findings will provide new empirical evidence about

whether these training sessions are effective and may inform the design of any future work to increase the use of SPC charts. To the research team's knowledge, this is the first project to directly evaluate the effectiveness of such training using a controlled before and after analysis of the documents the training should influence.

There are some limitations to our approach that stem from our time and resource constraints, as well as the nature of the retrospective evaluation. One is about the validity of our outcome measures. Although our use of publicly available board papers does overcome potential errors resulting from self-reported data, such as social desirability bias and recall errors (Groves et al, 2011), it may not capture all of the ways that trusts use SPC charts. For example, trusts may increase their use of SPC charts in other routine monitoring reports. This would decrease the validity of our findings. However, it is not possible to assess the impact of this issue without further investigation with more time and resources, and we leave it for future research. Further, as the board papers comprise many sub-reports, and are monitored by top-level decision-makers, they serve as the best publically available documents for the present evaluation.

Another limitation relates to the precision of our estimates. It may be that having more pre and post intervention time period measurements would increase our precision. Given resource constraints, a decision was taken to include external matched controls rather than additional time series data. We may, therefore, sacrifice some precision for more plausible causal inference. Trusts who receive the training later on may get swept up in a 'rising tide' of greater use of SPC charts by trusts in general – and so the training could appear to be effective, even if it was not relatively effective within the context of greater usage overall (Chen et al., 2016). The external controls approach allows us to evaluate the rising tide phenomenon, although it is not a perfect solution. Control trusts were selected to be similar to intervention trusts on observable characteristics, and it is possible that control trusts will differ according to unobservable characteristics, such as motivation or openness to change, which could bias the results.

Finally, generalisability is an issue. We study a sample of self-selecting trusts that elected to take part in a training intervention. As such, our results may not apply to any mandated training initiatives if these become a requirement. To put this another way, trusts that elect to be part of the training may be more susceptible to change than other trusts that may not come

willingly. Moreover, because we limit our sample to acute trusts, our results may not hold when extended to other forms of trusts – such as mental health or community care trusts. That said, it seems unlikely that other types of healthcare institutions or that hospitals elsewhere would be 'immune' from the influence of training. While there may be quantitative differences, we consider it unlikely that there will be qualitative differences. Similarly, because we limited our investigation to trusts in England, the generalisability of our results may not hold in other geographic areas.

References

Anhøj J, Hellesøe AM. The problem with red, amber, green: the need to avoid distraction by random variation in organisational performance measures. BMJ Qual Saf. 2017 Jan 1;26(1):81-4.

Benning A, Dixon-Woods M, Nwulu U, Ghaleb M, Dawson J, Barber N, Franklin BD, Girling A, Hemming K, Carmalt M, Rudge G. Multiple component patient safety intervention in English hospitals: controlled evaluation of second phase. BMJ. 2011 Feb 3;342:d199. https://doi.org/10.1136/bmj.d199

Bird, P. Understanding the right and wrong time for intervention. Use of charts by hospital boards can help decision makers set control limits to performance indicating where intervention may be required. Health Service Journal; 2017 14 Feb.

Braun V, Clarke V. What can "thematic analysis" offer health and wellbeing researchers?. International Journal of Qualitative Studies on Health and Well-being. 2014;9. https://doi.org/10.3402/qhw.v9.26152

Britto MT, Fuller SC, Kaplan HC, Kotagal U, Lannon C, Margolis PA, Muething SE, Schoettker PJ, Seid M. Using a network organisational architecture to support the development of Learning Healthcare Systems. BMJ Quality & Safety. 2018 Nov 1;27(11):937-46. doi: 10.1136/bmjqs-2017-007219.

Chen YF, Hemming K, Stevens AJ, Lilford RJ. Secular trends and evaluation of complex interventions: the rising tide phenomenon. BMJ Quality & Safety. 2016 May 1;25(5):303-10. https://doi.org/10.1136/bmjqs-2015-004372

Deblois S, Lepanto L. Lean and Six Sigma in acute care: a systematic review of reviews. International Journal of Health Care Quality Assurance. 2016 Mar 14. https://doi.org/10.1108/IJHCQA-05-2014-0058

Deeny SR, Steventon A. Making sense of the shadows: priorities for creating a learning healthcare system based on routinely collected data. BMJ Qual Saf. 2015 Aug 1;24(8):505-15. doi:10.1136/bmjqs-2015-004278.

Frison L, Pocock SJ. Repeated measures in clinical trials: analysis using mean summary statistics and its implications for design. Statistics in Medicine. 1992;11(13):1685-704. https://doi.org/10.1002/sim.4780111304

Goodman D, Ogrinc G, Davies L, Baker GR, Barnsteiner J, Foster TC, Gali K, Hilden J, Horwitz L, Kaplan HC, Leis J. Explanation and elaboration of the SQUIRE (Standards for Quality Improvement Reporting Excellence) Guidelines, V. 2.0: examples of SQUIRE elements in the healthcare improvement literature. BMJ Qual Saf. 2016 Dec 1;25(12):e7. https://doi.org/10.1136/bmjqs-2015-004411

Groves RM, Fowler Jr FJ, Couper MP, Lepkowski JM, Singer E, Tourangeau R. Survey methodology. John Wiley & Sons; 2011 Sep 20.

Hemming K, Haines TP, Chilton PJ, Girling AJ, Lilford RJ. The stepped wedge cluster randomised trial: rationale, design, analysis, and reporting. BMJ. 2015 Feb 6;350:h391. https://doi.org/10.1136/bmj.h391

Hoffmann TC, Glasziou PP, Boutron I, Milne R, Perera R, Moher D, Altman DG, Barbour V, Macdonald H, Johnston M, Lamb SE. Better reporting of interventions: template for

intervention description and replication (TIDieR) checklist and guide. BMJ. 2014 Mar 7;348:g1687. https://doi.org/10.1136/bmj.g1687

Institute of Medicine. Fostering rapid advances in healthcare: Learning from system demonstrations. Washington, DC: National Academies Press; 2002. https://doi.org/10.17226/10565

Kaplan HC, Lannon C, Walsh MC, Donovan EF, Ohio Perinatal Quality Collaborative. Ohio statewide quality-improvement collaborative to reduce late-onset sepsis in preterm infants. Pediatrics. 2011 Mar 1;127(3):427-35. doi: 10.1542/peds.2010-2141.

Larsson J, Landstad BJ, Wiklund H, Vinberg S. Control charts as an early-warning system for workplace health outcomes. Work. 2011 Jan 1;39(4):409-25. doi: 10.3233/WOR-2011-1191.

Lilford RJ, Chilton PJ, Hemming K, Girling AJ, Taylor CA, Barach P. Evaluating policy and service interventions: framework to guide selection and interpretation of study end points. BMJ. 2010 Aug 27;341:c4413. https://doi.org/10.1136/bmj.c4413

Mazzocato P, Holden RJ, Brommels M, Aronsson H, Bäckman U, Elg M, Thor J. How does lean work in emergency care? A case study of a lean-inspired intervention at the Astrid Lindgren Children's hospital, Stockholm, Sweden. BMC Health Services Research. 2012 Dec;12(1):28. doi: 10.1186/1472-6963-12-28.

Michie S, Van Stralen MM, West R. The behaviour change wheel: a new method for characterising and designing behaviour change interventions. Implementation Science. 2011 Dec 1;6(1):42. https://doi.org/10.1186/1748-5908-6-42

Mohammed MA, Cheng KK, Rouse A, Marshall T. Bristol, Shipman, and clinical governance: Shewhart's forgotten lessons. The Lancet. 2001 Feb 10;357(9254):463-7. https://doi.org/10.1016/s0140-6736(00)04019-8

NHS Digital (2020). Peer Finder Too. Retrieved 18 May 2020 from https://digital.nhs.uk/data-and-information/data-tools-and-services/data-services/innovative-uses-of-data/multi-dataset-analysis/nhs-trust-peer-finder-tool

NHS Improvement. Making Data Count website. Retrieved 14 August 2019 from https://improvement.nhs.uk/resources/making-data-count/

Schmidtke KA, Poots AJ, Carpio J, Vlaev I, Kandala NB, Lilford RJ. Considering chance in quality and safety performance measures: an analysis of performance reports by boards in English NHS trusts. BMJ Qual Saf. 2017 Jan 1;26(1):61-9. https://doi.org/10.1136/bmjqs-2015-004967

Schmidtke KA, Watson DG, Vlaev I. The use of control charts by laypeople and hospital decision-makers for guiding decision making. The Quarterly Journal of Experimental Psychology. 2017 Jul 3;70(7):1114-28. https://doi.org/10.1080/17470218.2016.1172096

Shewhart, W. Statistical method from the viewpoint of quality control. Dover 1986 edition with foreword by W. Edwards Deming, originally published 1939. Mineola, N.W: Dover Publications.

Tversky A, Kahneman D. Judgment under uncertainty: Heuristics and biases. Science. 1974 Sep 27;185(4157):1124-31. https://doi.org/10.1126/science.185.4157.1124

Wheeler, W. Contra two sigma: the consequences of using the wrong limits. Quarterly Digest Daily, 2013 1 May, Manuscript 255. Retrieved from: https://www.spcpress.com/pdf/DJW255.pdf
