# Supplementary material for: Retrospective evaluation of an intervention based on training sessions to increase the use of control charts in hospitals
Source: BMJ Qual Saf. 2022 Jun 24;32(2):100–8. doi: 10.1136/bmjqs-2021-013514 (PMC9887349; doi:10.1136/bmjqs-2021-013514)
Supplement: Supplementary data [file bmjqs-2021-013514supp002.pdf]

Supplementary 2 – SQUIRE checklist

Reporting checklist for quality improvement in health care.

Based on the SQUIRE guidelines.

		Reporting Item	Page Number *[refers to submission document, not published document]*
**Title**			
	#1	Indicate that the manuscript concerns an initiative to improve healthcare (broadly defined to include the quality, safety, effectiveness, patientcenteredness, timeliness, cost, efficiency, and equity of healthcare)	Title page
**Abstract**			
	#02a	Provide adequate information to aid in searching and indexing	Abstract (0)
	#02b	Summarize all key information from various sections of the text using the abstract format of the intended publication or a structured summary such as: background, local problem, methods, interventions, results, conclusions	Abstract (0)
**Introduction**			
Problem description	#3	Nature and significance of the local problem	1=
Available knowledge	#4	Summary of what is currently known about the problem, including relevant previous studies	2-3
Rationale	#5	Informal or formal frameworks, models, concepts, and / or theories used to explain the problem, any	2-3

Supplementary 2 – SQUIRE checklist

		reasons or assumptions that were used to develop the intervention(s), and reasons why the intervention(s) was expected to work	
Specific aims	#6	Purpose of the project and of this report	3
**Methods**			
Context	#7	Contextual elements considered important at the outset of introducing the intervention(s)	3
Intervention(s)	#08a	Description of the intervention(s) in sufficient detail that others could reproduce it	4
Intervention(s)	#08b	Specifics of the team involved in the work	3
Study of the Intervention(s)	#09a	Approach chosen for assessing the impact of the intervention(s)	5-6
Study of the Intervention(s)	#09b	Approach used to establish whether the observed outcomes were due to the intervention(s)	5-6
Measures	#10a	Measures chosen for studying processes and outcomes of the intervention(s), including rationale for choosing them, their operational definitions, and their validity and reliability	6-8
Measures	#10b	Description of the approach to the ongoing assessment of contextual elements that contributed to the success, failure, efficiency, and cost	7-8
Measures	#10c	Methods employed for assessing completeness and accuracy of data	7
Analysis	#11a	Qualitative and quantitative methods used to draw inferences from the data	8-10
Analysis	#11b	Methods for understanding variation within the data, including the effects of time as a variable	8
Ethical considerations	#12	Ethical aspects of implementing and studying the intervention(s) and how they were addressed, including, but not limited to, formal ethics review and potential conflict(s) of interest	9
**Results**			

	#13a	Initial steps of the intervention(s) and their evolution over time (e.g., time-line diagram, flow chart, or table), including modifications made to the intervention during the project	Fig 4, S4, S5
	#13b	Details of the process measures and outcome	17-18
	#13c	Contextual elements that interacted with the intervention(s)	N/A
	#13d	Observed associations between outcomes, interventions, and relevant contextual elements	9-16
	#13e	Unintended consequences such as unexpected benefits, problems, failures, or costs associated with the intervention(s).	N/A
	#13f	Details about missing data	N/A
**Discussion**			
Summary	#14a	Key findings, including relevance to the rationale and specific aims	18
Summary	#14b	Particular strengths of the project	18
Interpretation	#15a	Nature of the association between the intervention(s) and the outcomes	18
Interpretation	#15b	Comparison of results with findings from other publications	18-19
Interpretation	#15c	Impact of the project on people and systems	18-21
Interpretation	#15d	Reasons for any differences between observed and anticipated outcomes, including the influence of context	21
Interpretation	#15e	Costs and strategic trade-offs, including opportunity costs	N/A
Limitations	#16a	Limits to the generalizability of the work	23
Limitations	#16b	Factors that might have limited internal validity such as confounding, bias, or imprecision in the design, methods, measurement, or analysis	19-21

Supplementary 2 – SQUIRE checklist

Limitations	#16c	Efforts made to minimize and adjust for limitations	19-21
Conclusion	#17a	Usefulness of the work	21
Conclusion	#17b	Sustainability	21
Conclusion	#17c	Potential for spread to other contexts	21
Conclusion	#17d	Implications for practice and for further study in the field	21
Conclusion	#17e	Suggested next steps	21
**Other information**			
Funding	#18	Sources of funding that supported this work. Role, if any, of the funding organization in the design, implementation, interpretation, and reporting	29-30

None The SQUIRE 2.0 checklist is distributed under the terms of the Creative Commons Attribution License CC BY-NC 4.0. This checklist can be completed online using https://www.goodreports.org/, a tool made by the EQUATOR Network in collaboration with Penelope.ai
