# Supplementary material for: Retrospective evaluation of an intervention based on training sessions to increase the use of control charts in hospitals
Source: BMJ Qual Saf. 2022 Jun 24;32(2):100–8. doi: 10.1136/bmjqs-2021-013514 (PMC9887349; doi:10.1136/bmjqs-2021-013514)
Supplement: Supplementary data [file bmjqs-2021-013514supp003.pdf]

Supplementary 3 – TIDieR checklist

Item No	Item	Current Research
**Brief name**		
1	Provide the name or a phrase that describes the intervention	Making Data Count SPC Training Sessions
**Why**		
2	Describe any rationale, theory, or goal of the elements essential to the intervention	Trainings are conducted because improved knowledge about statistical process control charts (SPCs) may increase their uptake and then prevent unnecessary interventions in the NHS
**What**		
3	Materials: Describe any physical or informational materials used in the intervention, including those provided to participants or used in intervention delivery or in training of intervention providers. Provide information on where the materials can be accessed (such as online appendix, URL)	Two examples of PowerPoints used in the training are shown in Supplementary Materials 4 and 5. The two Making Data Count guidebooks are available online (https://improvement.nhs.uk/resources/making-data-count/). The training events cover the strengths and weaknesses of presenting data in different ways, and include background on what SPCs are, when and how to use them, why they should be used, and how they can improve decision making. Topics include identifying trends (e.g. seven points in one direction), special versus common cause variation, and summarising data using icons (see Supplementary Materials 4, Slide 47). The limitations of r-a-g systems are discussed, and, importantly, each trust's data is presented to them using control charts to demonstrate the value of using SPCs.

Supplementary 3 – TIDieR checklist

4	Procedures: Describe each of the procedures, activities, and/or processes used in the intervention, including any enabling or support activities	Two examples of PowerPoints used in the training are shown in Supplementary Materials 4 and 5. The two Making Data Count guidebooks are available online (https://england.nhs.uk/resources/making-data-count/)
**Who provided**		
5	For each category of intervention provider (such as psychologist, nursing assistant), describe their expertise, background, and any specific training given	Trainings were delivered by two experienced trainers from NHS Improvement with higher educational background in statistics and work experience in data analytics.
**How**		
6	Describe the modes of delivery (such as face to face or by some other mechanism, such as internet or telephone) of the intervention and whether it was provided individually or in a group	One trainer visited each trust to deliver the training face-to-face to board members and, separately, to teams of analysts and ambassadors.
**Where**		
7	Describe the type(s) of location(s) where the intervention occurred, including any necessary infrastructure or relevant features	The trainings were delivered in meeting rooms at each Trust, which varied in the available facilities
**When and How Much**		

Supplementary 3 – TIDieR checklist

8	Describe the number of times the intervention was delivered and over what period of time including the number of sessions, their schedule, and their duration, intensity, or dose	The trainings were delivered to board members and, separately, to teams of analysts and ambassadors. Board and analyst trainings were not necessarily given on the same day and could be separated by around a month. Board training sessions are delivered over about 90 minutes while training for analysts teams is delivered over one work day.
**Tailoring**		
9	If the intervention was planned to be personalised, titrated or adapted, then describe what, why, when, and how	Trusts' data were presented to them using control charts to demonstrate the value of using SPCs
**Modifications**		
10*	If the intervention was modified during the course of the study, describe the changes (what, why, when, and how)	N/A
**How well**		
11	Planned: If intervention adherence or fidelity was assessed, describe how and by whom, and if any strategies were used to maintain or improve fidelity, describe them	N/A

Supplementary 3 – TIDieR checklist

12*	Actual: If intervention adherence or fidelity was assessed, describe the extent to which the intervention was delivered as planned	N/A
*If checklist is completed for a protocol, these items are not relevant to protocol and cannot be described until study is complete.		
