# Supplementary material for: Retrospective evaluation of an intervention based on training sessions to increase the use of control charts in hospitals
Source: BMJ Qual Saf. 2022 Jun 24;32(2):100–8. doi: 10.1136/bmjqs-2021-013514 (PMC9887349; doi:10.1136/bmjqs-2021-013514)
Supplement: Supplementary data [file bmjqs-2021-013514supp004.pdf]

Supplementary 4 - Making Data Count Powerpoint (1)

NHS Improvement

Making data count

[redacted]

Samantha Riley, Head of Improvement Analytics
[redacted]

collaboration trust respect innovation courage compassion

Where we are now.............

NHS Improvement

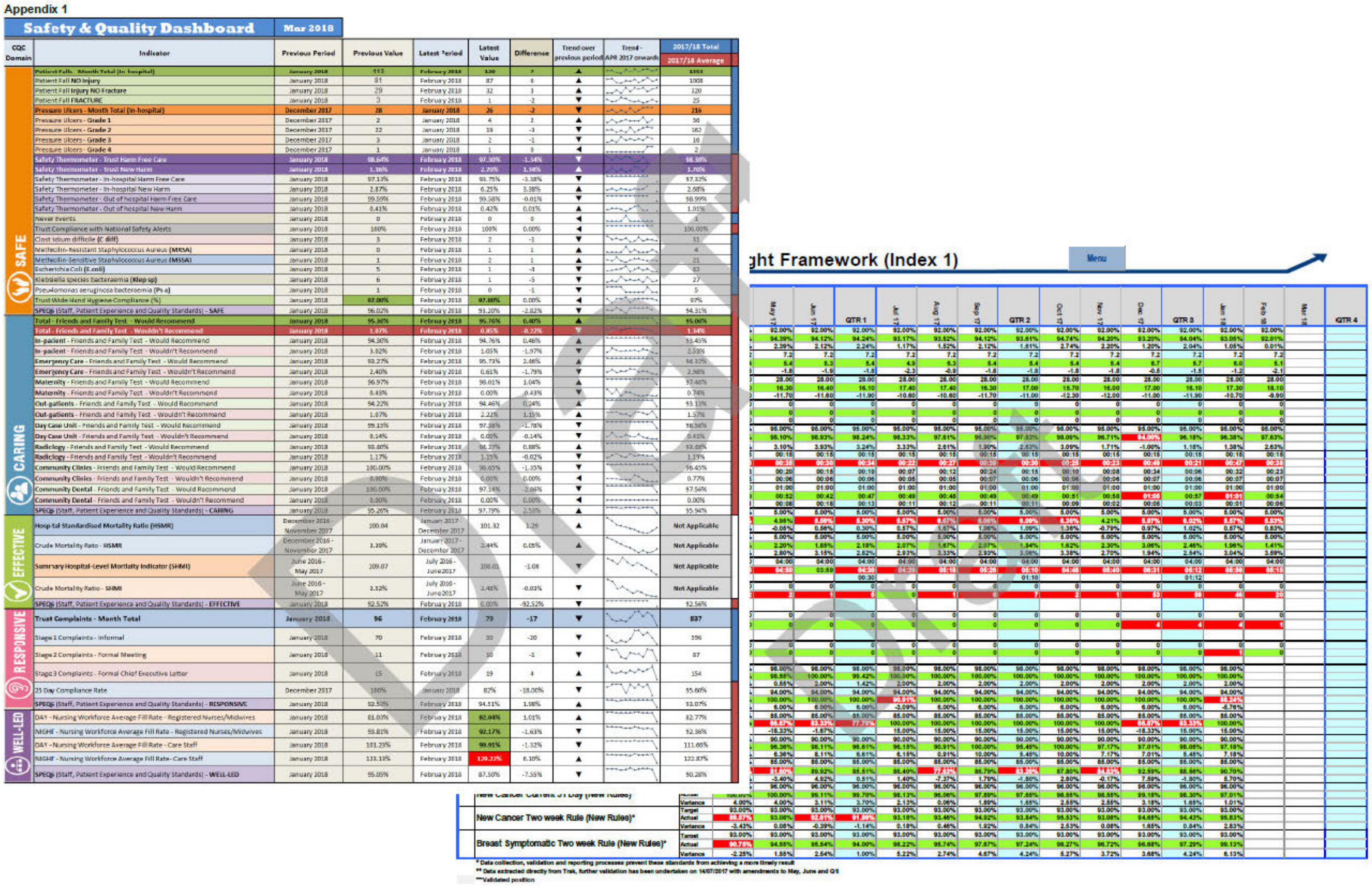

The importance of focus

NHS Improvement

Safety & Quality Dashboard | **Mar 2018**

CQC Domain	Indicator	Previous Period	Previous Value	Latest Period	Latest Value	Difference	Trend over previous period	Trend - APR 2017 onwards	2017/18 Total / 2017/18 Average
	Emergency Care - Friends and Family Test - Would Recommend	January 2018	93.27%	February 2018	95.73%	2.46%	▲		94.32%

Trend over previous period
▲

→ One month trend…….

Is an increase from 95.36% to 95.76% important or distracting narrative?

Caring

7 Family and Friends Test (FFT) (data up to February 2018)

7.2 The Trusts 'Would Recommend' for Friends and Family returns *increased* to 95.76% for February 2018 from 95.36% in January 2018. The percentage of patients who stated they 'Wouldn't Recommend' *decreased* to 0.85% in February 2018 from 1.07% in January 2018.

[redacted]

4

NHS Improvement

% Delayed transfers of Care by Type - source SITREPS 7/1/02-31/08/03

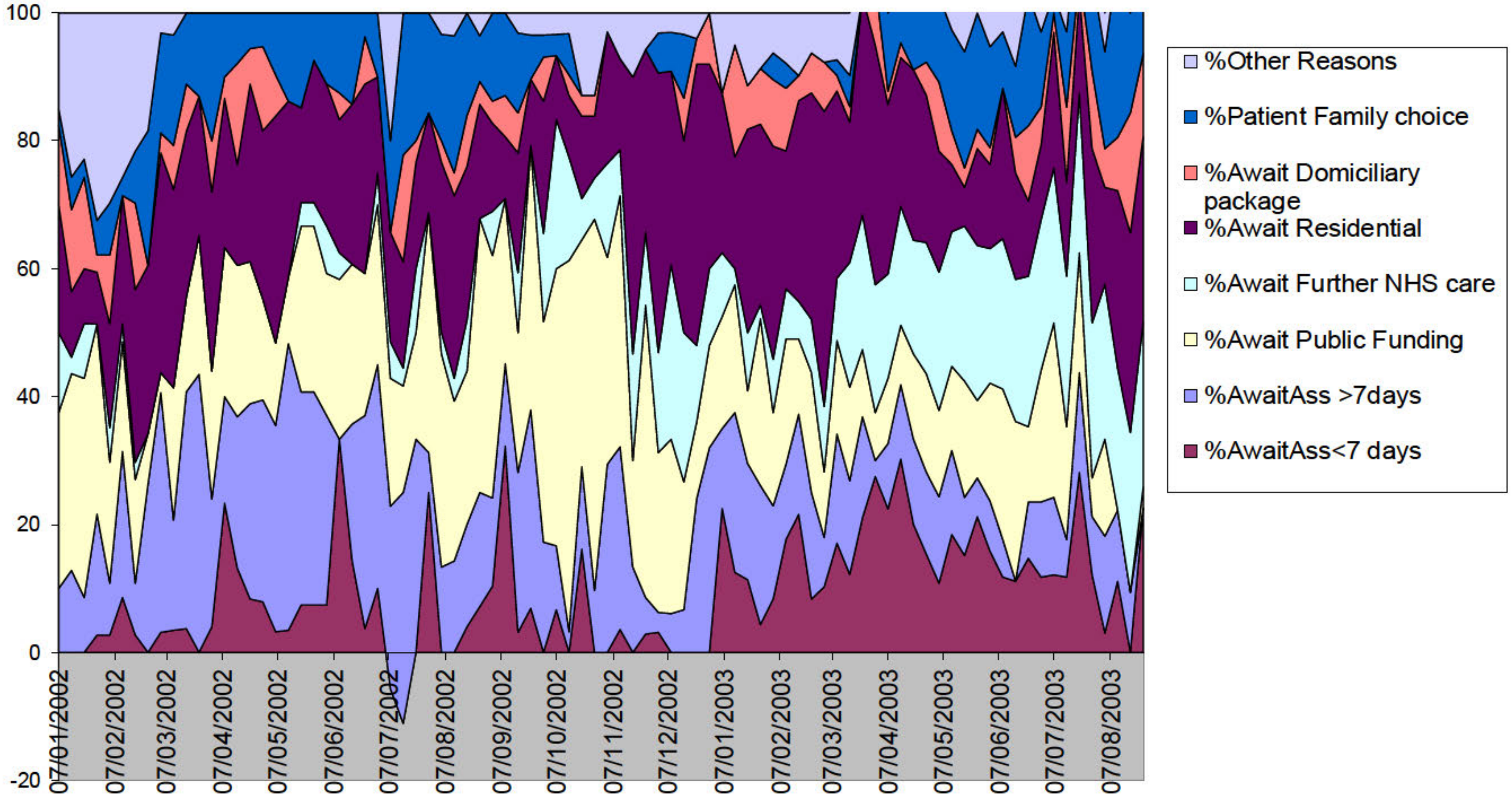

NHS Improvement

Activities summary from the monthly measures : Mar'02-

	Patients within the scope of the project treated this month		Patients in scope of the project treated this month under an agreed Care Pathway		Time* from referral to first definitive treatment (days)		Maximum wait* for 1st specialist appointment		Average wait* for 1st specialist appointment		Booked appointments through new clinics		Booked admissions through new clinics		Number of Patient Discovery Interviews		Team self-assessment
Pilot sites	Mar '02	change from last month	Mar '02	change from last month	Mar '02	change from last month	Mar '02	change from last month	Mar '02	change from last month	Mar '02	change from last month	Mar '02	change from last month	Mar '02	change from last month	Mar '02
	-	0	-	0	184	-17	-	-140	45	-2	278	54	14	4	-	0	-
	8	-1	8	8	741	434	175	-7	123	-4	0	0	0	0	0	-1	2
	-	0	-	0	-	-8	-	-70	-	-30	-	0	-	0	-	0	-
	97	22	17	11	-	-16	84	0	57	-4	17	2	7	7	0	0	4
	37	-19	12	6	41	-65	84	0	82	22	0	0	0	0	0	0	-
	-	-38	-	0	-	-669	-	-182	-	-123	-	0	-	-48	-	0	-
	15	0	15	0	294	-34	245	14	84	0	0	0	0	-87	0	0	3
	366	24	291	3	395	48	55	10	39	-8	0	-379	0	0	0	0	4
	-	-46	0	-46	282	-52	235	64	119	0	1217	46	0	0	0	0	4
	15	2	3	3	-	0	97	-8	23	1	699	238	6	0	-	0	3
	29	-4	9	3	369	53	228	-49	186	37	0	0	0	0	8	8	2
	18	10	14	9	200	-40	32	1	30	2	0	0	39	2	0	0	-
	175	-77	38	-88	172	-1	137	11	45	5	0	0	18	3	0	0	-
	25	8	25	8	1	-1	98	-14	77	14	123	-3	62	-6	14	4	3
	-	0	-	0	-	0	-	-618	-	-106	-	0	-	0	-	0	-
	30	12	15	5	318	0	89	-155	209	43	0	0	0	0	0	0	3
	11	-3	0	0	400	90	214	-47	210	13	0	0	0	0	0	0	-
	-	0	-	0	-	-416	-	-297	-	-91	-	0	0	-29	9	-9	4
	12	2	12	2	210	113	306	-250	114	43	0	0	0	0	0	0	-
	-	-4	-	-4	204	57	222	-445	192	99	0	0	0	0	0	0	-
	3	0	3	0	226	-81	322	-195	195	104	0	0	0	0	0	0	-

NHS Improvement

Safer Staffing Report	Assessment			Medical			Stroke			Surgical		
	Current month	Last month	Year to date	Current month	Last month	Year to date	Current month	Last month	Year to date	Current month	Last month	Year to date
Day fill rate	104	80	99	101	79	104	96	86	87	94	101	104
Night fill rate	94	70	101	105	104	93	72	97	100	85	94	71
Sickness	20	39	24	30	36	32	39	29	38	27	37	28
Vacancy	23	21	35	39	37	37	26	39	21	39	30	21

NHS Improvement

Improving Access to Psychological Therapies – performance against target

Metric	Target	Jan-17	Feb-17	Mar-17	Apr-17	May-17	Jun-17	Jul-17	Aug-17
IAPT Treatment 18 Weeks	95%	100.0%	99.5%	99.9%	99.8%	99.4%	99.7%	99.6%	99.7%
IAPT Treatment 6 Weeks	75%	86%	84%	83%	81%	75%	80%	81%	81%
IAPT Recovery Rate	50%	59%	57%	54%	55%	54%	52%	55%	55%
EIS First Episode Psychosis	50%	100%	100%	83%	63%	100%	89%	100%	85%

NHS Improvement

IAPT recovery rate

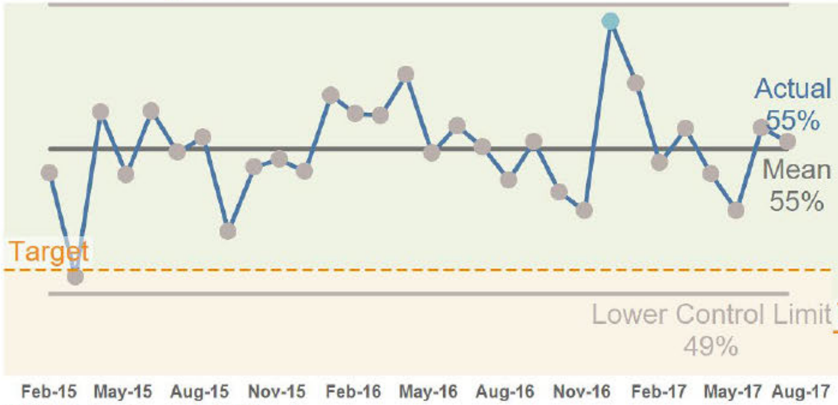

EIS - First Episode Of Psychosis

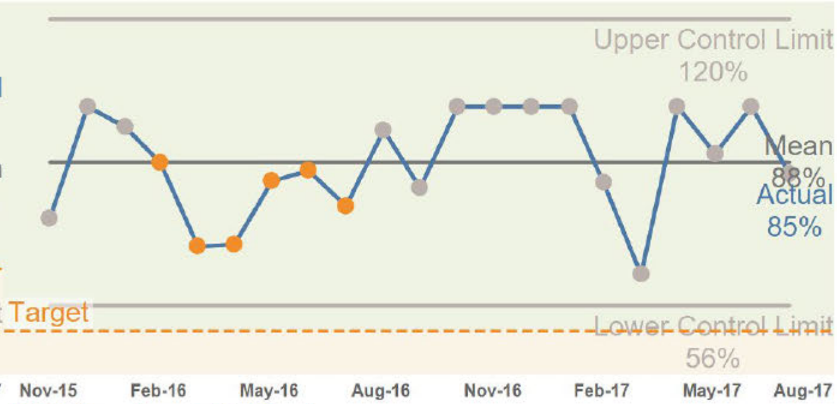

IAPT Treatment 6 Weeks

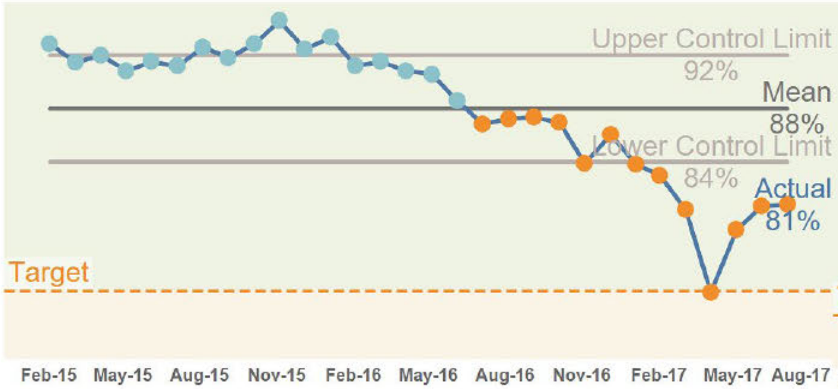

IAPT Treatment 18 Weeks

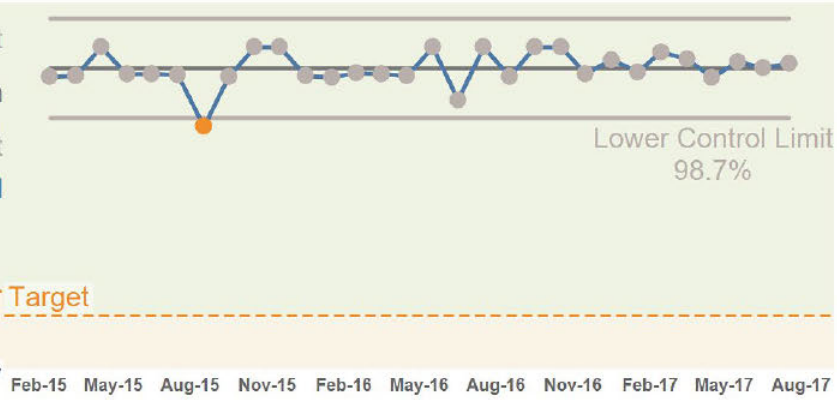

NHS Improvement

Did green provide true assurance?

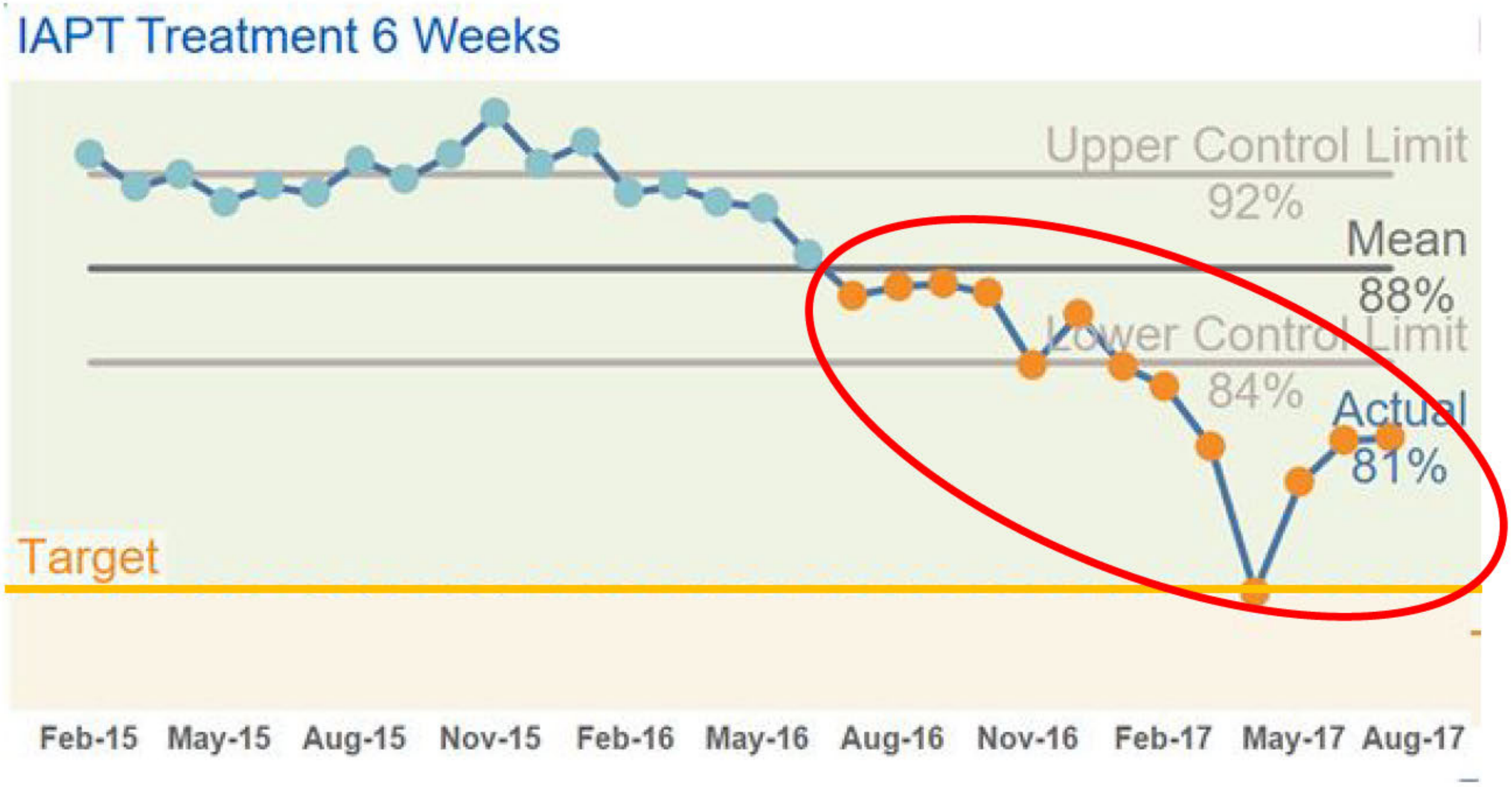

Introducing John and Mary

NHS Improvement

[redacted]

[redacted]

[redacted]

NHS Improvement

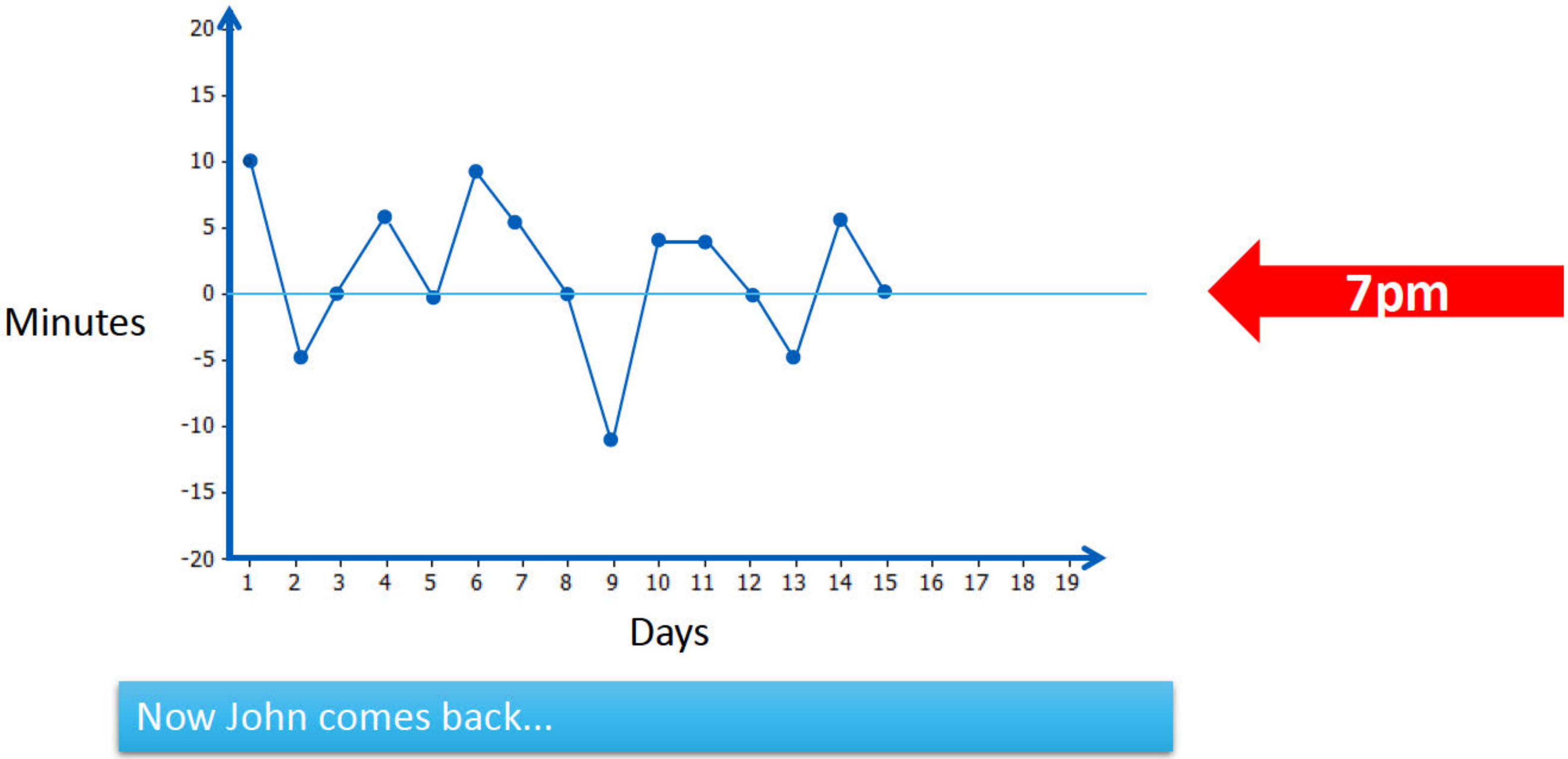

Kudrna L, *et al. BMJ Qual Saf* 2023; 32:100–108. doi: 10.1136/bmjqs-2021-013514

NHS Improvement

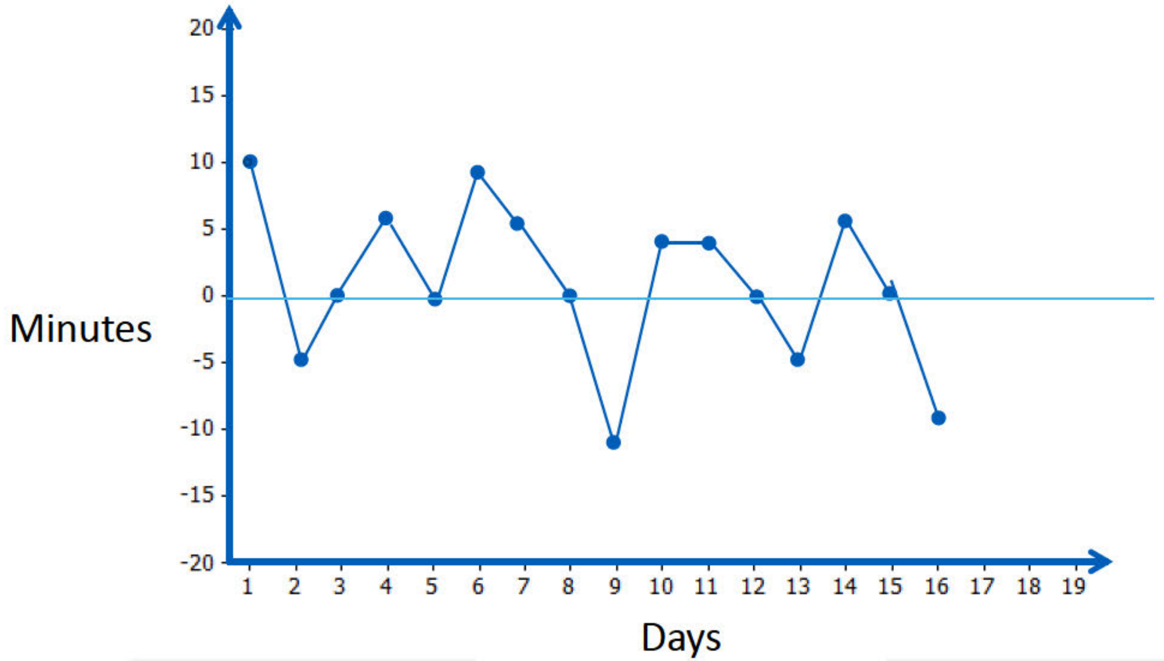

Mary arrives at 18:50

John asks, why have you arrived 10 minutes early?

NHS Improvement

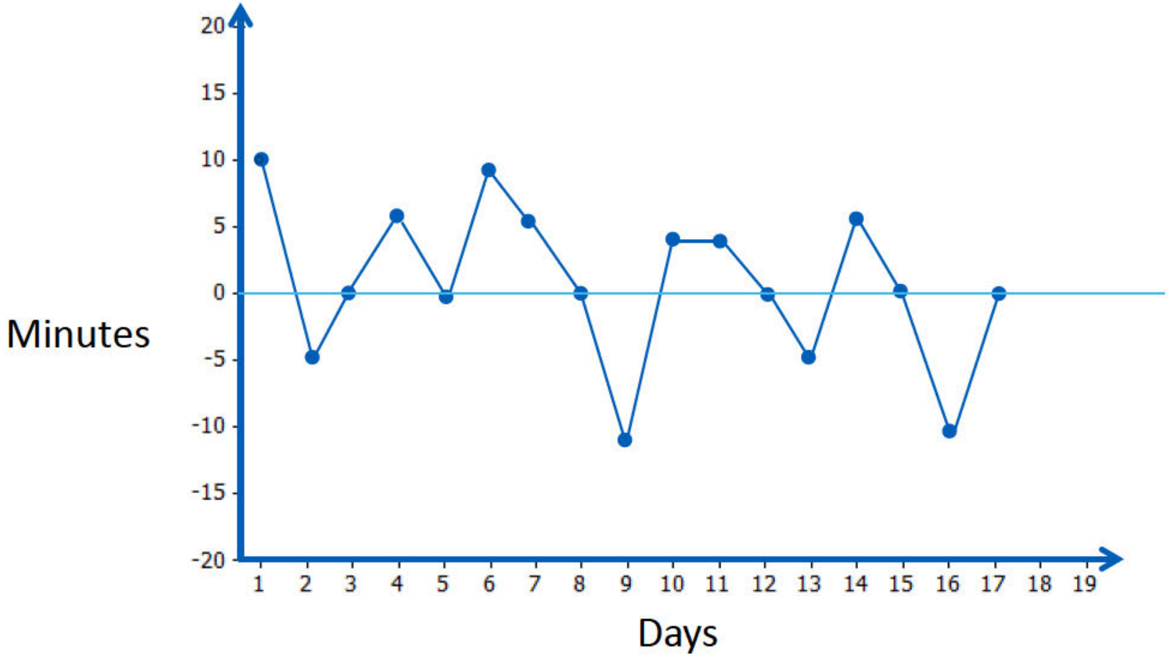

Mary arrives at 19:00.

John asks: yesterday you arrived at 18.50 – why have you arrived at 19:00 today?

NHS
Improvement

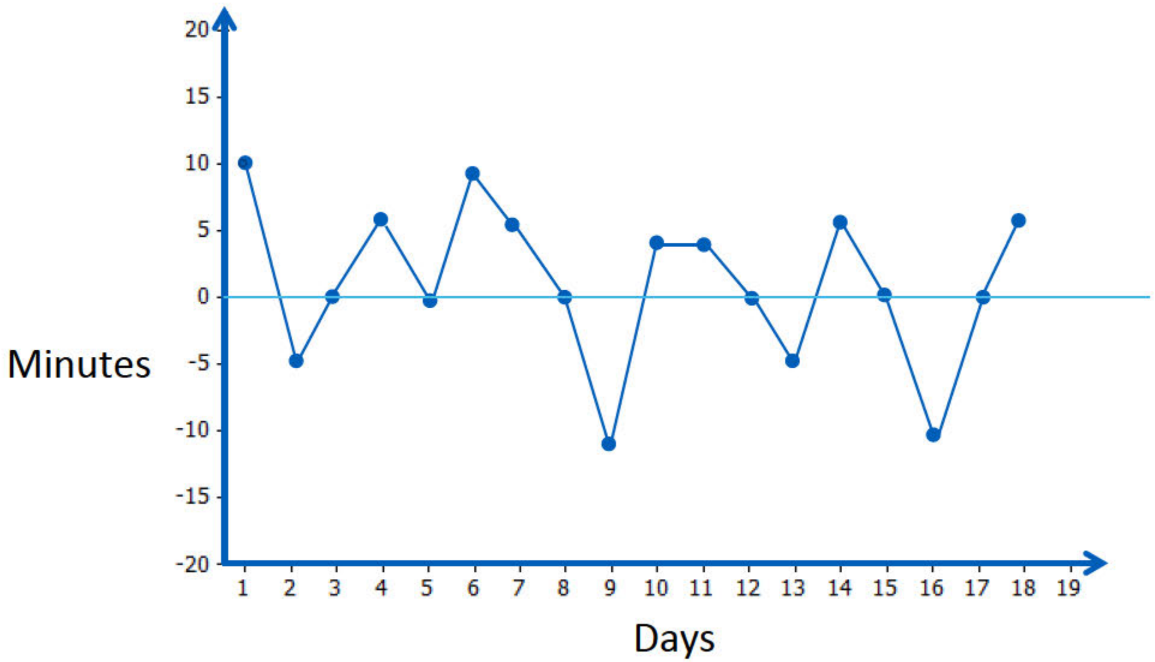

Mary arrives at 19:05

John asks: yesterday you arrived at 7pm – why are you late?

NHS Improvement

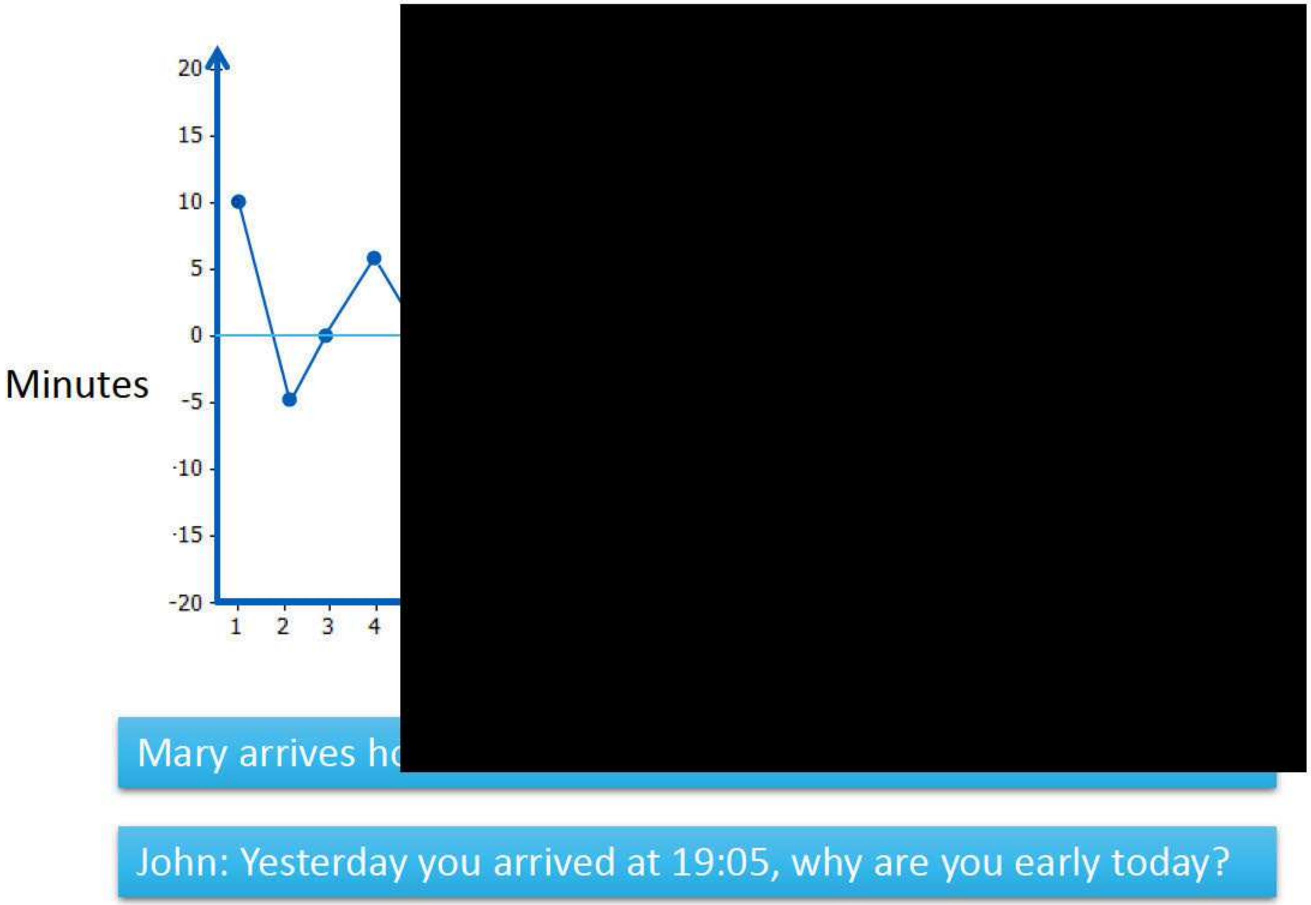

[redacted]

Mary arrives h[redacted]

John: Yesterday you arrived at 19:05, why are you early today?

Thoughts on John & Mary ?

NHS Improvement

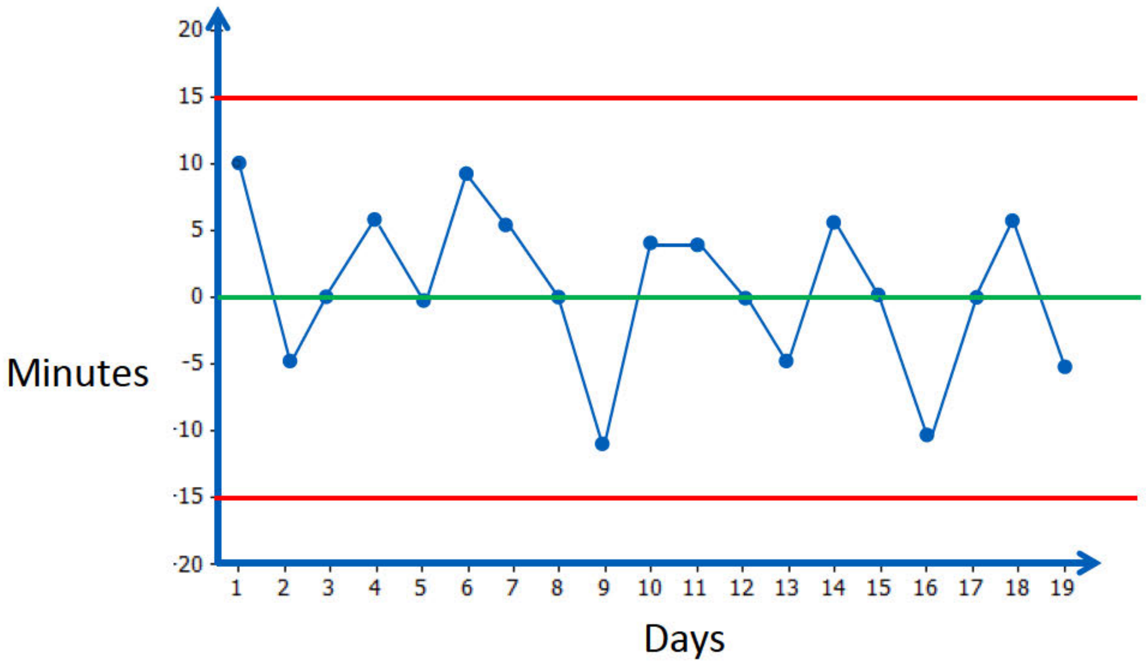

17 | Making data count

Frequently seen in the NHS

NHS Improvement

Spuddling

To make a lot of fuss about trivial things, as if they were important

Tampering

Scenario

We're going to simulate some **real data** in a healthcare setting

We'll be thinking about **how people react to patterns and trends** in data

Can you spot an **improvement or decline** when it occurs? We'll begin plotting our data in a **run chart**

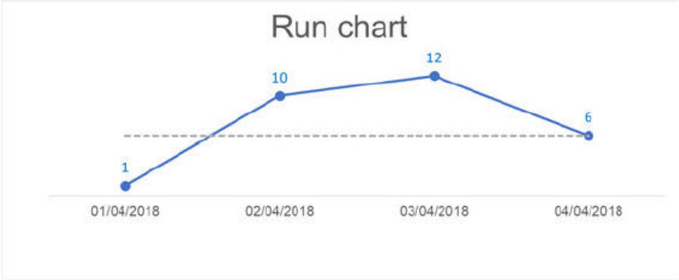

NHS Improvement

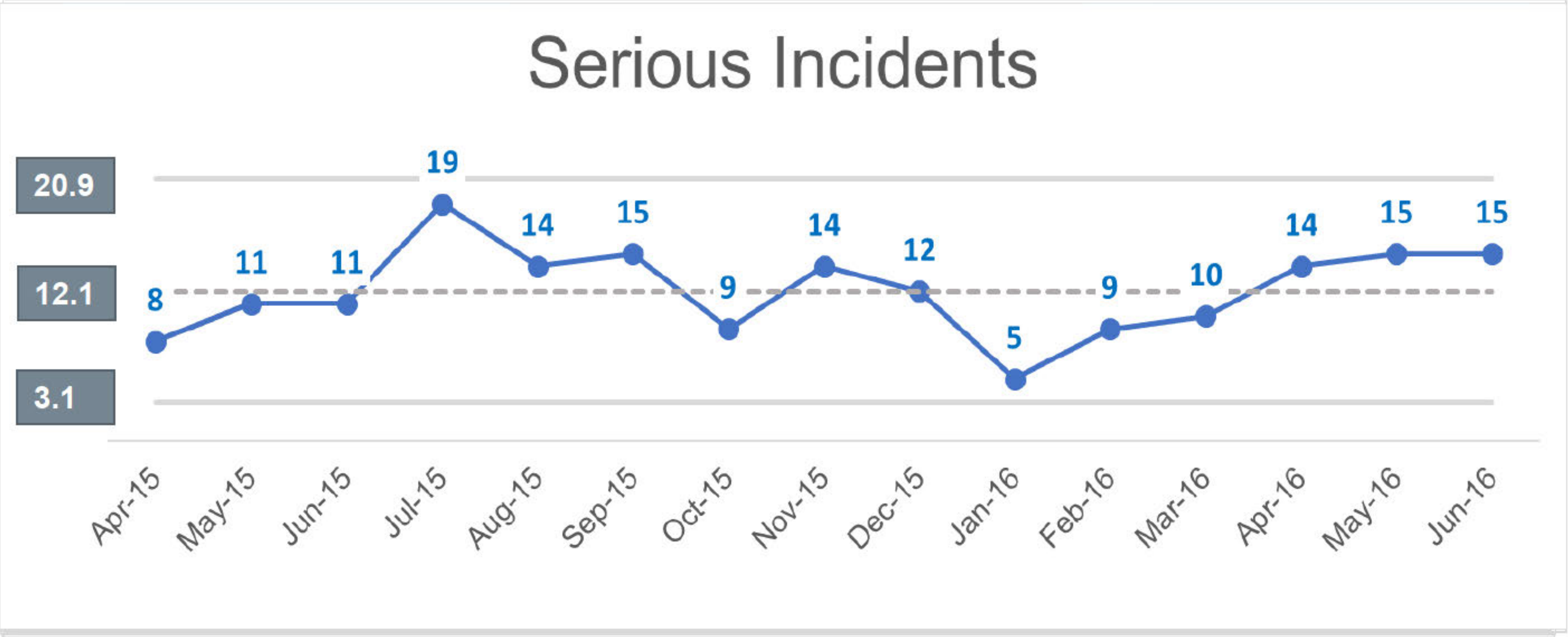

We now have enough data for robust process limits, lets change our run chart to an SPC chart

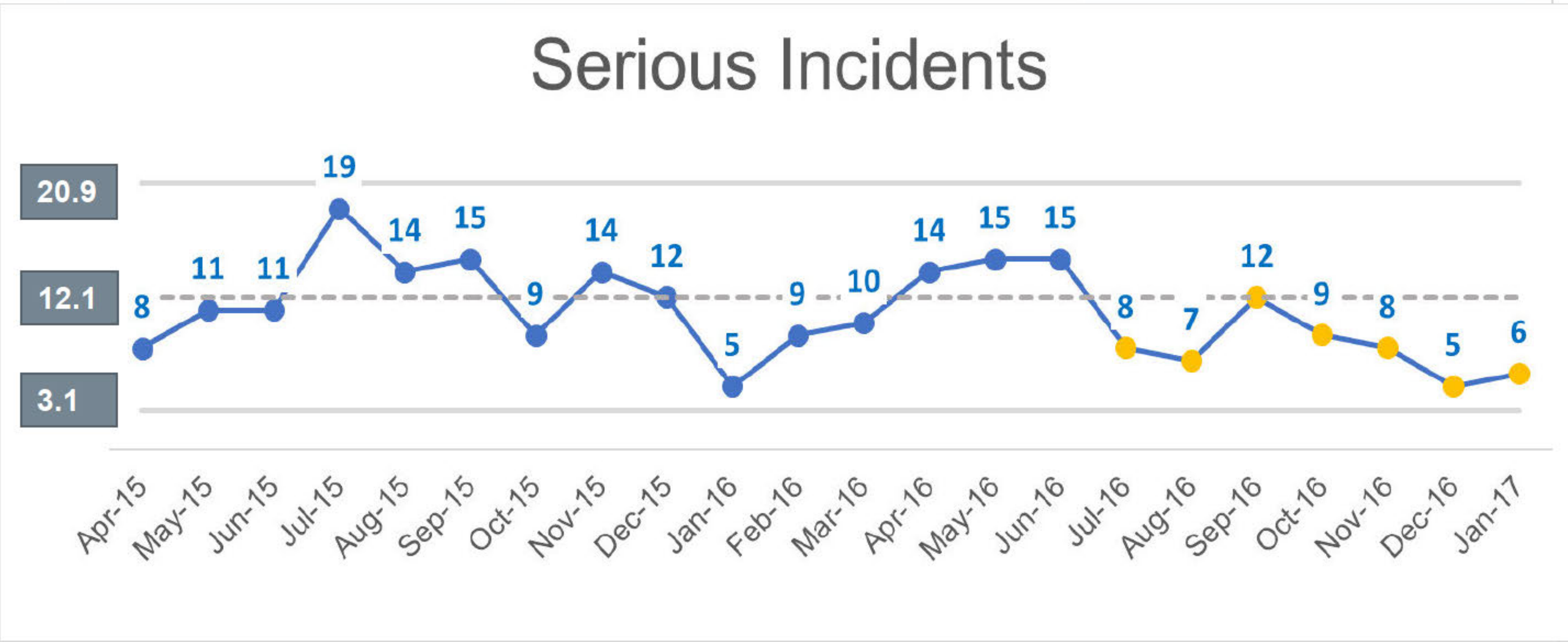

7 points below mean line put your hand if you think the improvement is successful

21 | Presentation title

NHS Improvement

This data set was randomly generated using the number of letters and consonants in Beatles number 1 singles

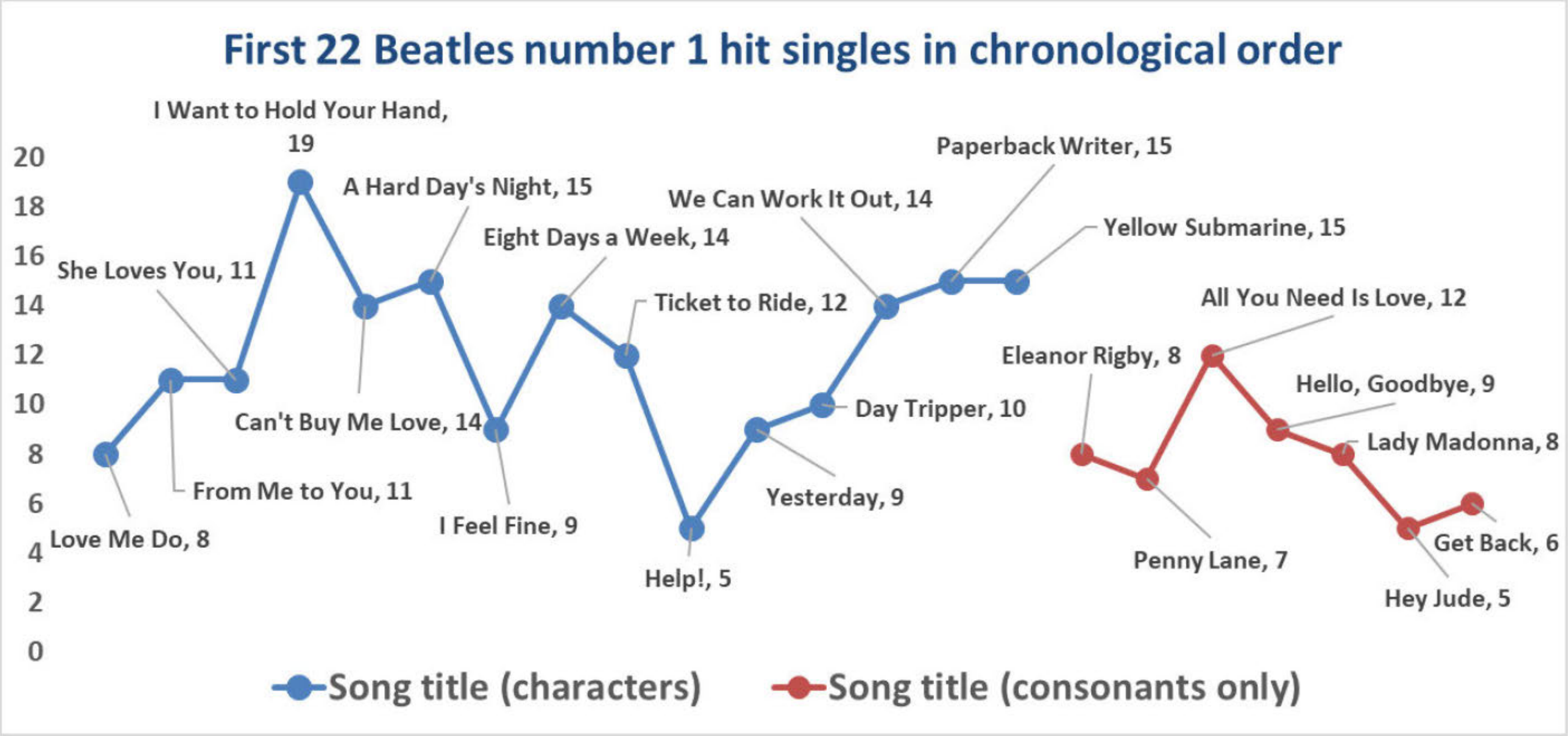

NHS Improvement

Strong evidence base

THE PROBLEM WITH...

The problem with red, amber, green: the need to avoid distraction by random variation in organisational performance measures

Jacob Anhøj, Anne-Marie Blok Hellesoe

Centre for Diagnostic Investigation, Rigshospitalet, University of Copenhagen, Copenhagen, Denmark

Correspondence to Dr Jacob Anhøj, Centre for Diagnostic Investigation, Rigshospitalet, University of Copenhagen, Blegdamsvej 9, Copenhagen 2100, Denmark; jacob@anhoej.net

Accepted 18 January 2016
Published Online First 31 March 2016

'The Problem with...' series covers controversial topics related to efforts to improve healthcare quality, including widely recommended but deceptively difficult strategies for improvement and pervasive problems that seem to resist solution.

INTRODUCTION

Many healthcare organisations now track a number of performance measures like infection and complication rates, waiting times, staff adherence to guidelines, etc. Our own organisation, The Capital Region of Denmark, provides healthcare for 1.7 million people and runs 6 hospitals and 11 mental health centres. Measures of clinical quality have been widely used in our region locally at hospitals and departments for many years. Recently, our region started to systematically define and track strategical key performance measures also at the top management level. Approximately 25 measures on a wide range of subjects from hospital infections to public transportation are being tracked by the top management and the Regional Council.

The measurement strategy for hospitals involves a bottom-up approach allowing each hospital and department to, if needed, define its own performance measures that feed into one or more of the overall measures. For example, bacteraemia is one of the overall measures, and some acute-care departments, who rarely see hospital-acquired bacteraemia, have started to work on reducing the use of bladder catheters in order to reduce the risk of bacteraemia from catheter-related urinary tract infections diagnosed after their patients have been transferred to other departments. To support their work, they have developed a handful of measures that track the use of catheters and staff compliance with standard procedures related to catheter use.

We welcome this development very much. The choice of relatively few overall measures combined with the bottom-up approach is a helpful strategy that focuses and aligns improvement work and stimulates the use of data at all levels of the organisation while leaving room for meaningful local adaptations of performance measures.

However, we do not at all welcome the widespread use of red, amber, green approaches to data analysis that is everywhere in our organisation.

By 'red, amber, green', we are referring to graphical data displays that use colour coding of individual data values based on whether this value is on the right (green) or wrong (red) side of a target value. Often amber or yellow is used to indicate data values that are somewhere between 'right' and 'wrong'.

The problem with red, amber, green management is that at best is it useless, at worst it is harmful.

THE PROBLEM WITH RED, AMBER, GREEN

Figure 1 was captured from the February 2015 report on regional performance measures. It shows the monthly count of a certain type of unwanted incident in mental healthcare. The horizontal line represents the target value of 10.5. That is, we do not want more than 10 incidents per month. Red bars show months above target. Green bars show months below target.

The data display in figure 1 is formally correct (green is better than red). However, it fails to convey a very

Linked

▸ http://dx.doi.org/10.1136/bmjqs-2015-004967
▸ http://dx.doi.org/10.1136/bmjqs-2016-005303

CrossMark

To cite: Anhøj J, Hellesoe A-MB. *BMJ Qual Saf* 2017;**26**:81–84.

BMJ   Anhøj J, Hellesoe A-MB. *BMJ Qual Saf* 2017;**26**:81–84. doi:10.1136/bmjqs-2015-004951   81

The anatomy of a SPC chart

NHS Improvement

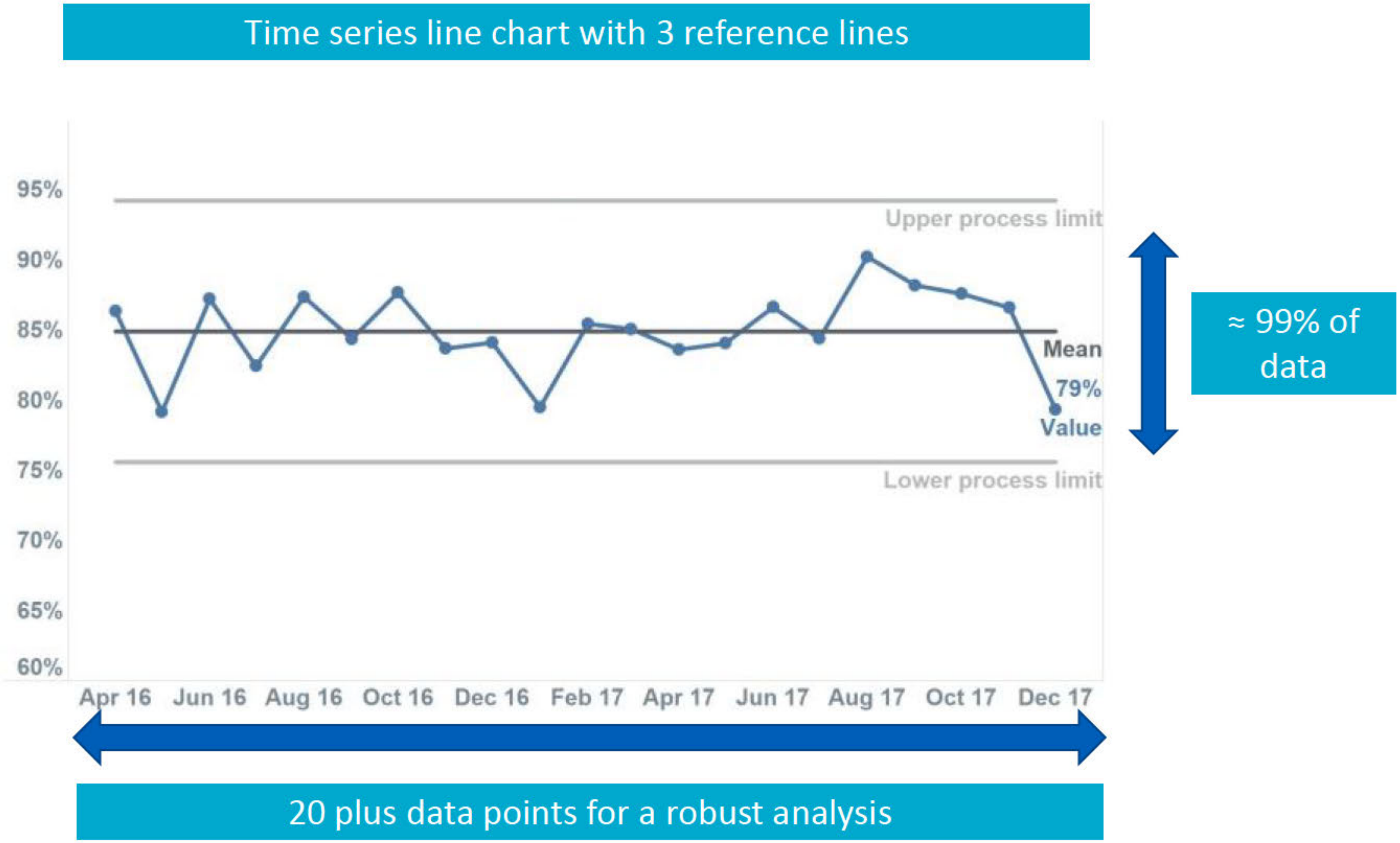

NHS Improvement

SPC rules : special cause variation

A single data point outside the process limits

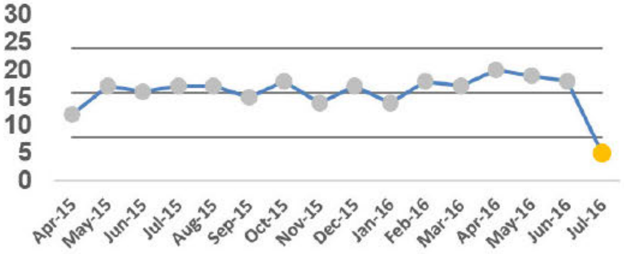

Two out of three points close to the process limits

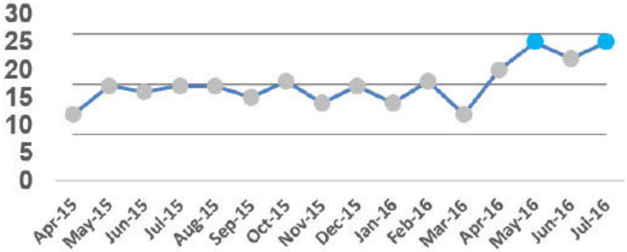

Shift of points above / below mean line

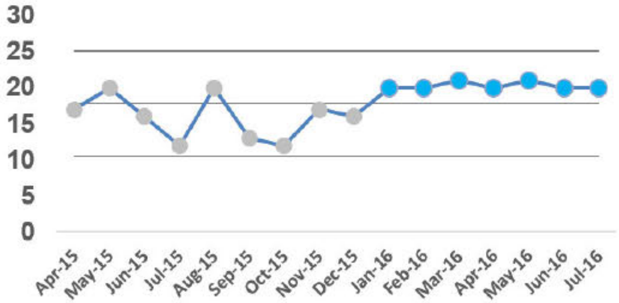

Run of points in consecutive ascending / descending order

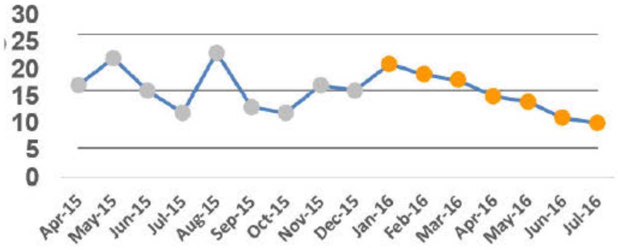

NHS Improvement

Why is 7 points significant?

[redacted]

A trend of 2 has the probability of 25% occurrence (**one in four**)

A trend of 4 has the probability of 6.25% occurrence (**one in sixteen**)

A trend of 6 has the probability of 1.56% occurrence (**one in sixty-four**)

A trend of 7 has the probability of 0.8% occurrence (**one in one hundred and twenty-eight**)

NHS Improvement

If there is special cause…..

[redacted]

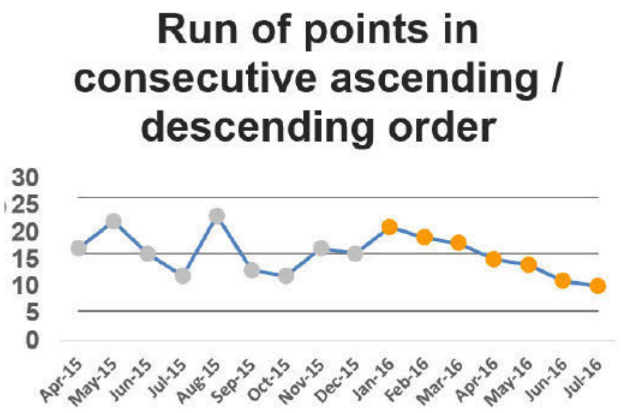

27 | Making data count

NHS Improvement

In control but unacceptable variation (common cause variation)

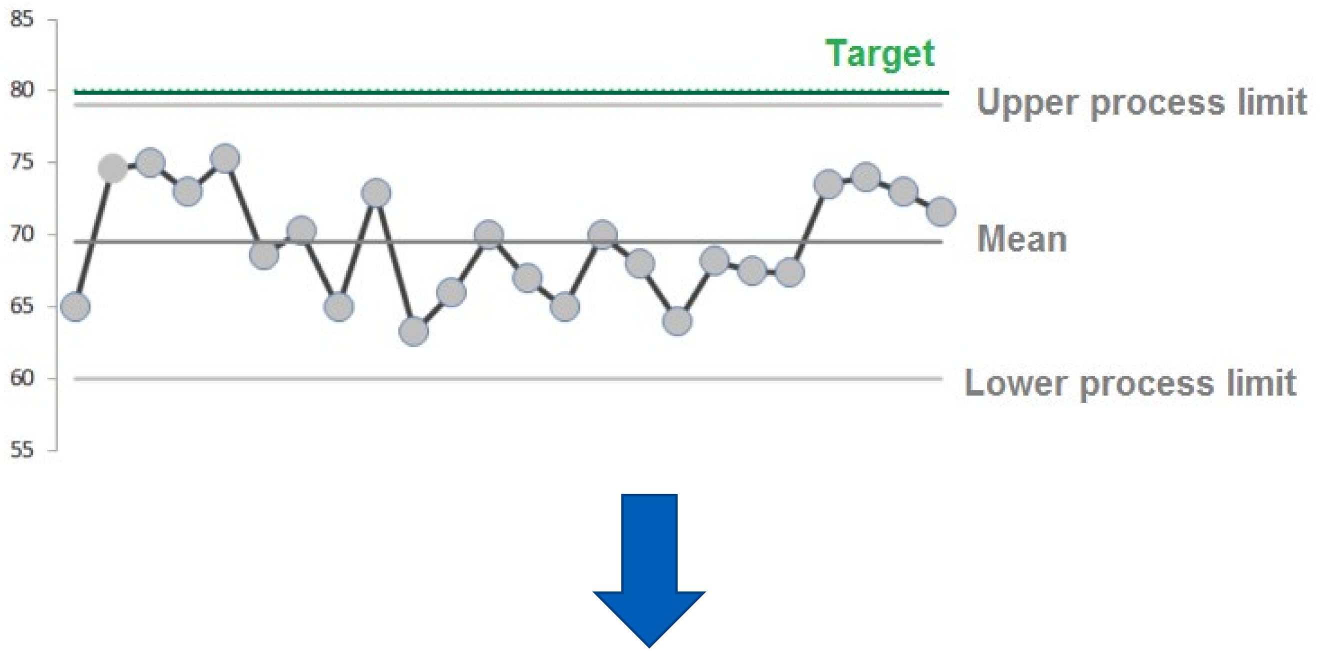

Redesign the system

Has the change worked?

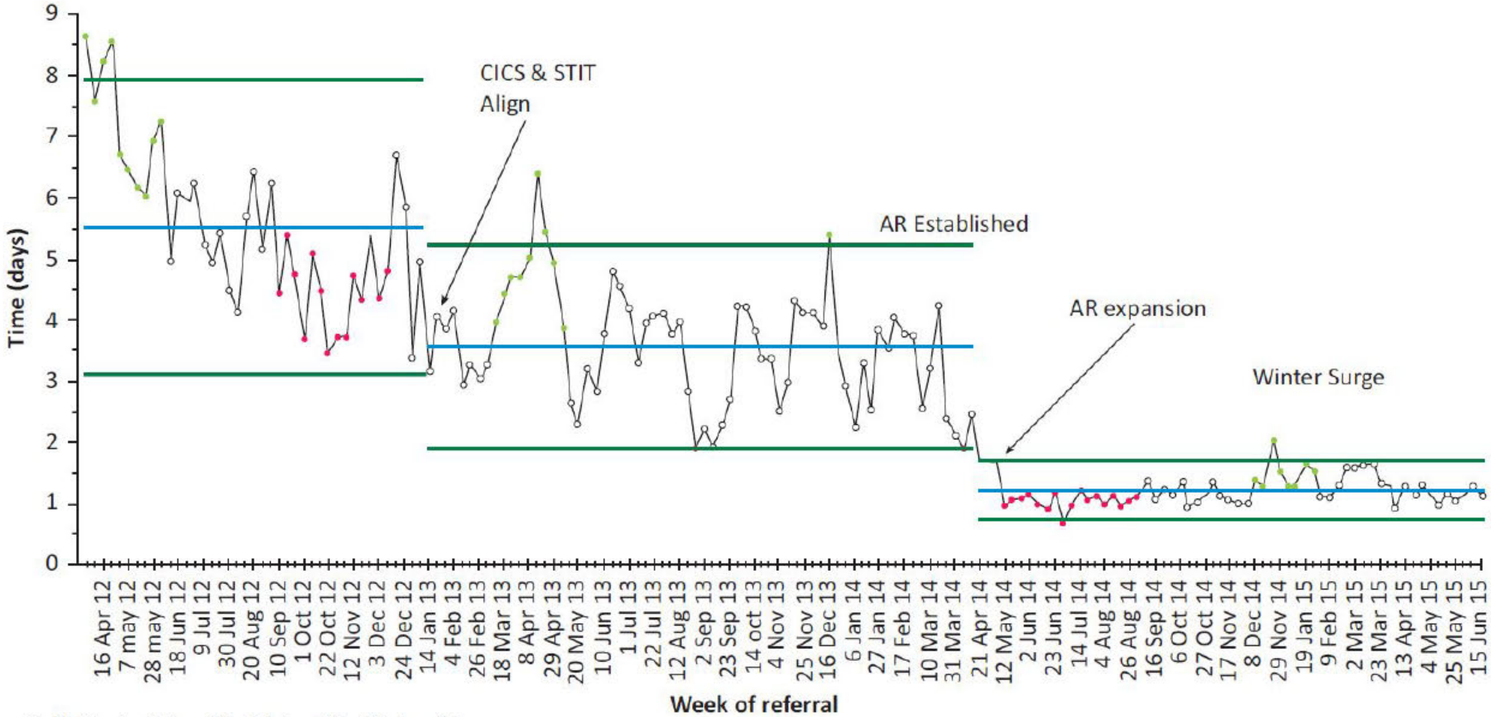

Split Start	2 Apr 12	14 Jan 13	21 Apr 14
U.C.L.	–7.9	5.2	1.7
Mean	–5.5	6.6	1.2

Fig 2. Reducing patient wait for active recovery from a hospital bed. AR = Active Recovery; CICS = Community Intermediate Care Service; STIT = Short Term Intervention Team

NHS Improvement

What extra insight could SPC provide?

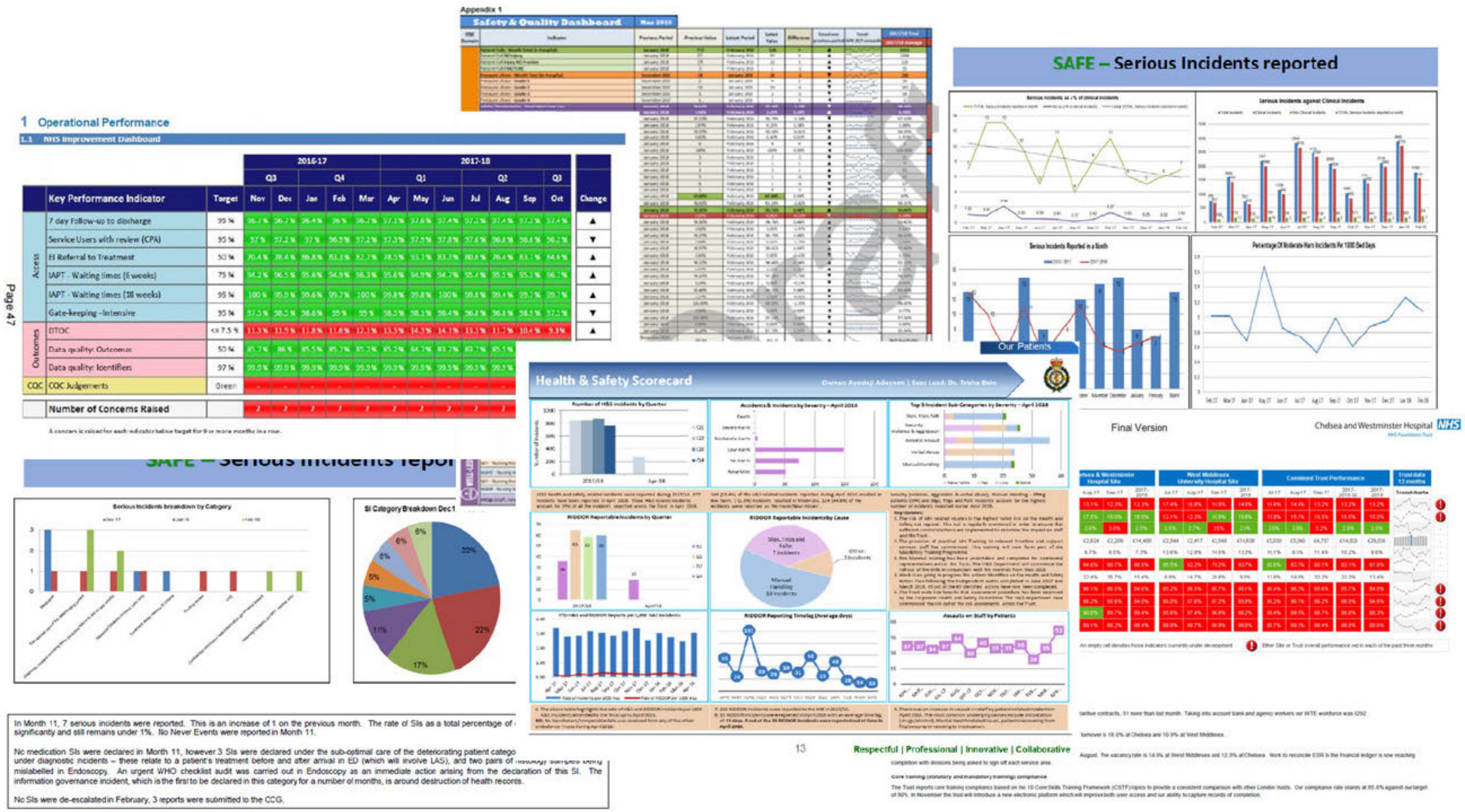

NHS Improvement

What do you think when you see this?

															Trust data 13 months
Domain	Indicator	Jul-17	Aug-17	Sep-17	2017-2018	Jul-17	Aug-17	Sep-17	2017-2018	Jul-17	Aug-17	Sep-17	2017-2018 Q2	2017-2018	Trend charts
Training	Mandatory training compliance (Target: >90%)	85.4%	86.1%	85.5%	84.6%	85.2%	86.5%	85.7%	85.1%	85.4%	86.2%	85.6%	85.7%	84.8%	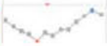

Presentation influences discussion

NHS Improvement

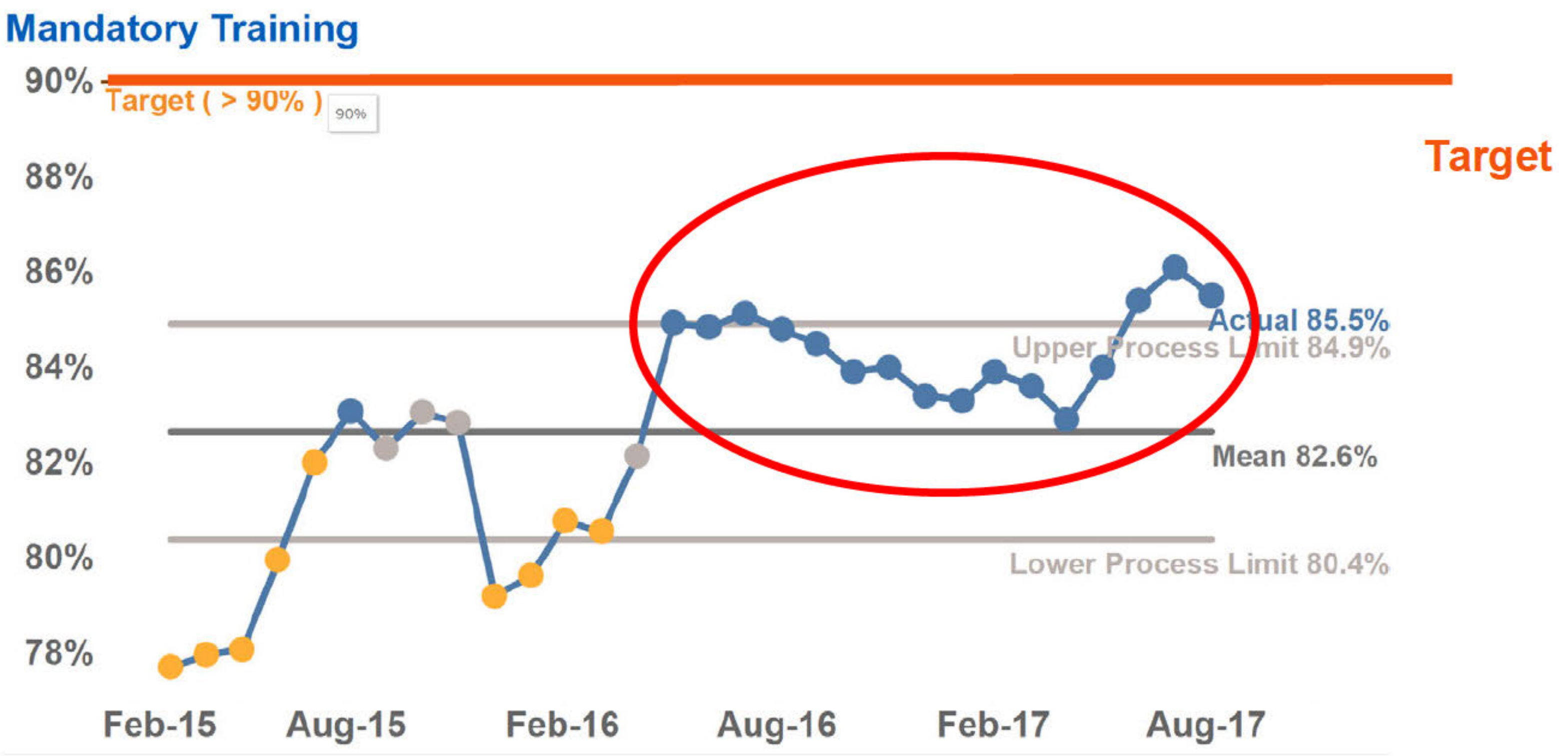

32 | Presentation title

NHS Improvement

Can you spot improvement?

Turnover trust wide (target 10%) ***source ESR***

This remains high for a number of factors, which includes service decommissioning and termination of a number of fixed term contract worker across numerous operational and corporate services.

Quarter 1						Quarter 2			
Apr-17	Apr-18	May-17	May-18	Jun-17	Jun-18	Jul-17	Jul-18	Aug-17	Aug-18
18.08%	11.19%	17.86%	11.95%	18.31%	12.40%	17.91%	12.20%	18.15%	12.10%

Improvement through the red

NHS Improvement

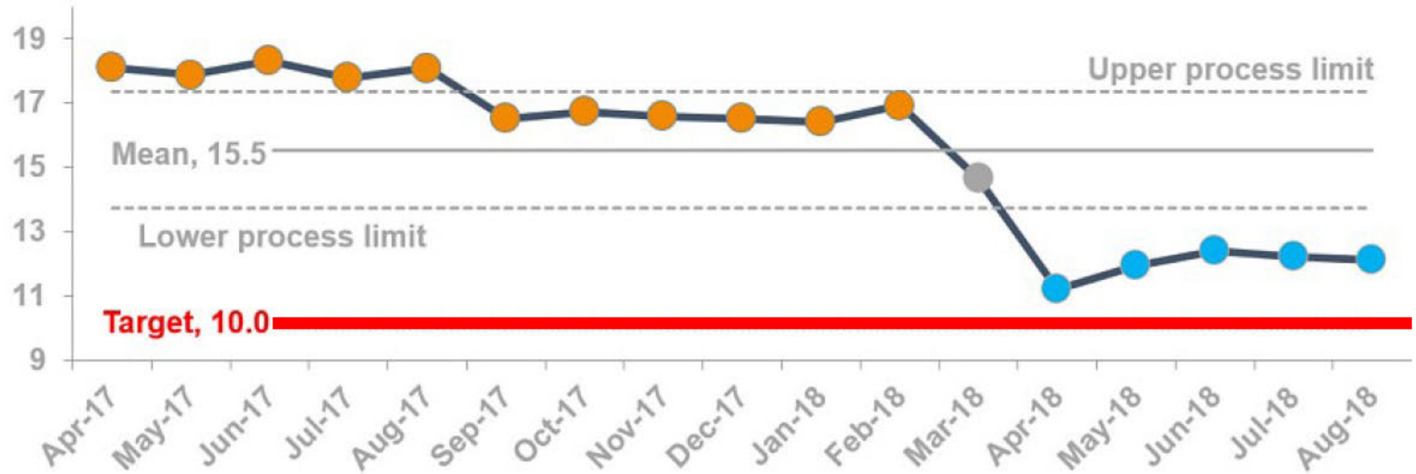

Turnover trust wide (target 10%) *source ESR*

This remains high for a number of factors, which includes service decommissioning and termination of a number of fixed term contract worker across numerous operational and corporate services.

Quarter 1						Quarter 2			
Apr-17	Apr-18	May-17	May-18	Jun-17	Jun-18	Jul-17	Jul-18	Aug-17	Aug-18
18.08%	11.19%	17.86%	11.95%	18.31%	12.40%	17.91%	12.20%	18.15%	12.10%

Encourages knee jerk reactions?

NHS Improvement

Caring Standards	Month 10	Month 11	Month 12	Month 1	Month 2	Month 3	Month 4	Month 5	Month 6	Month 7	Month 8	Month 9	Month 10	Month 11	FYTD Actual	YTD Target	Trend on Month
Friends and Family Test - % Likely to Recommend A&E	93.7	93.6	93.%	95.24	90.2	90.3	89.7	89.5	89.0	91.31	89.8	94.7	92.9	93.4	90.49	90.00	↑

System not capable

NHS Improvement

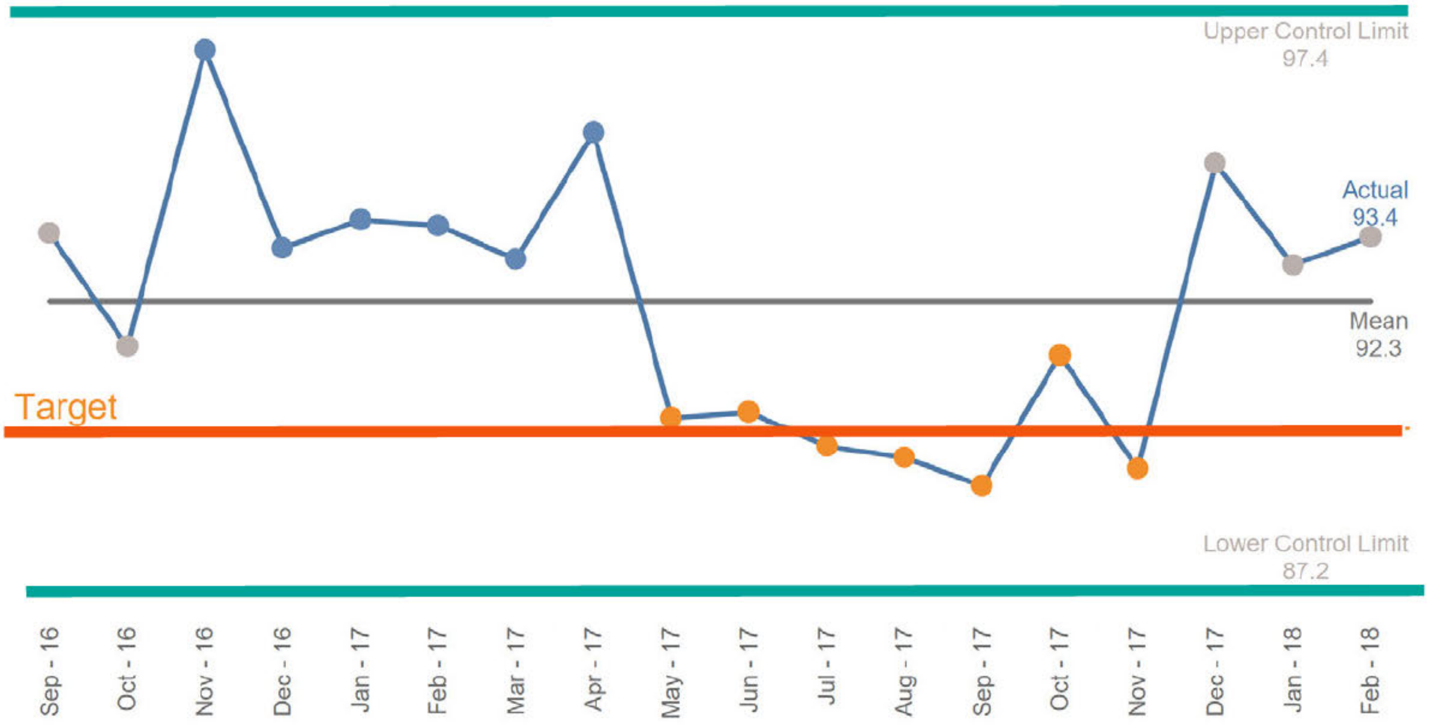

NHS Improvement

Serious incidents : 3 years

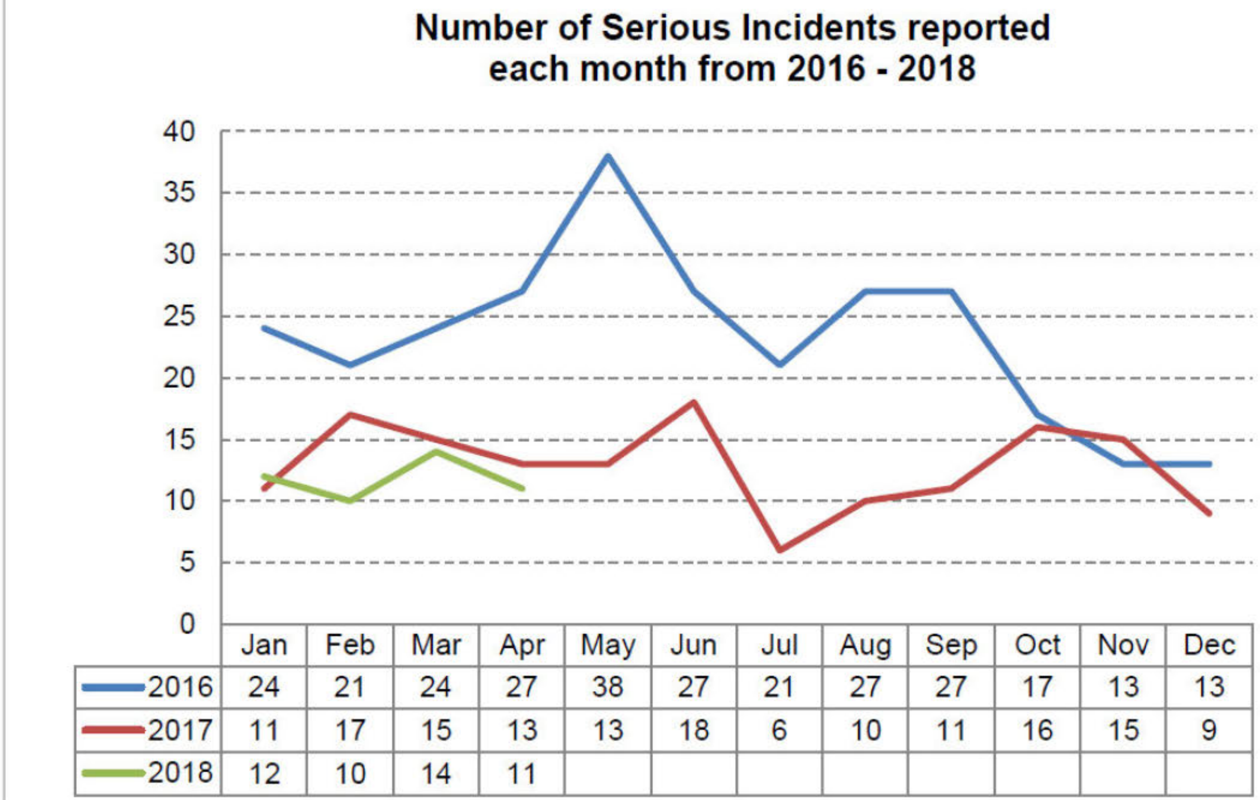

	Jan	Feb	Mar	Apr	May	Jun	Jul	Aug	Sep	Oct	Nov	Dec
2016	24	21	24	27	38	27	21	27	27	17	13	13
2017	11	17	15	13	13	18	6	10	11	16	15	9
2018	12	10	14	11								

NHS Improvement

Improvement (?)

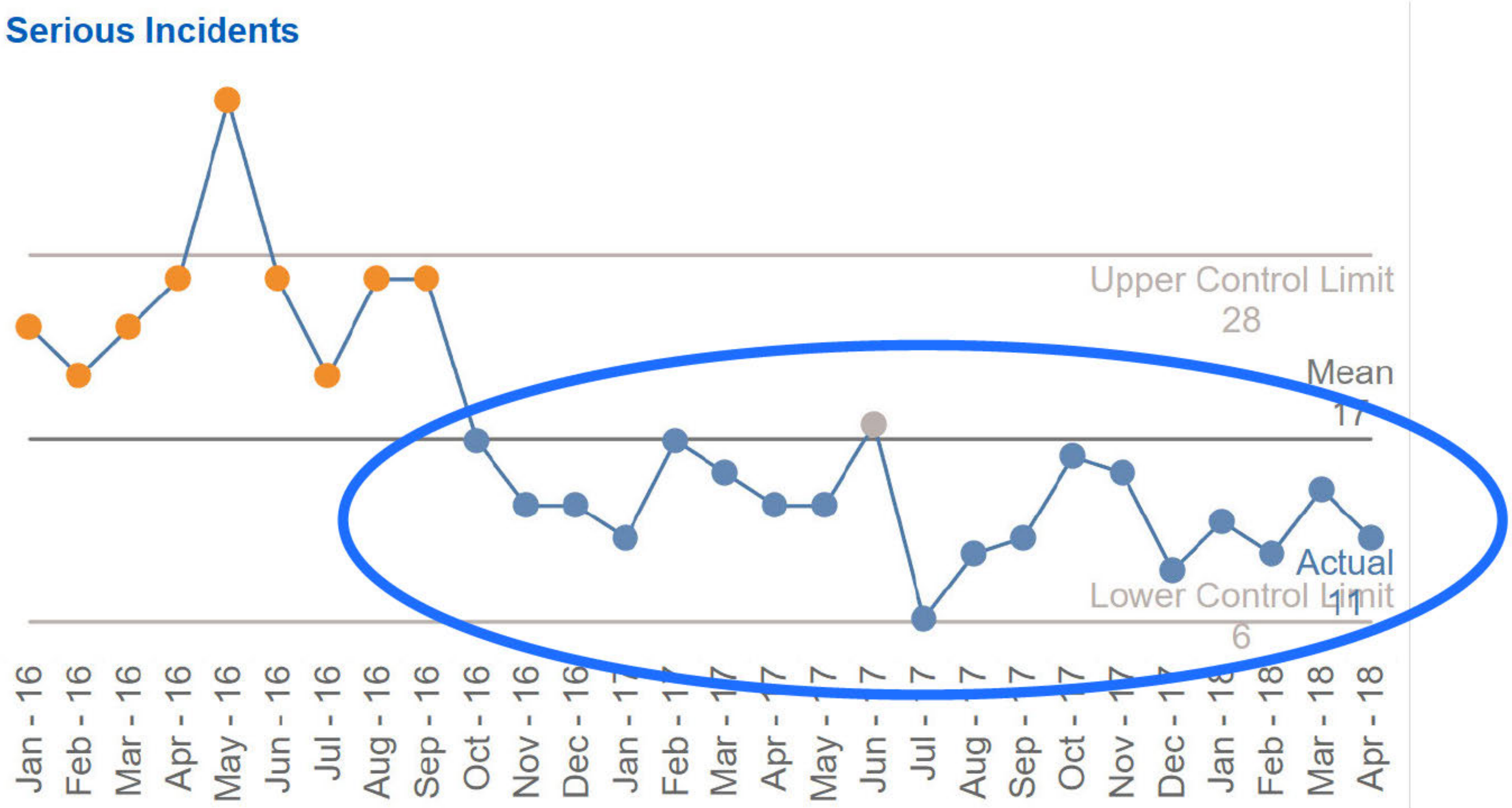

38 | Making data count

NHS Improvement

Spotting improvement and decline

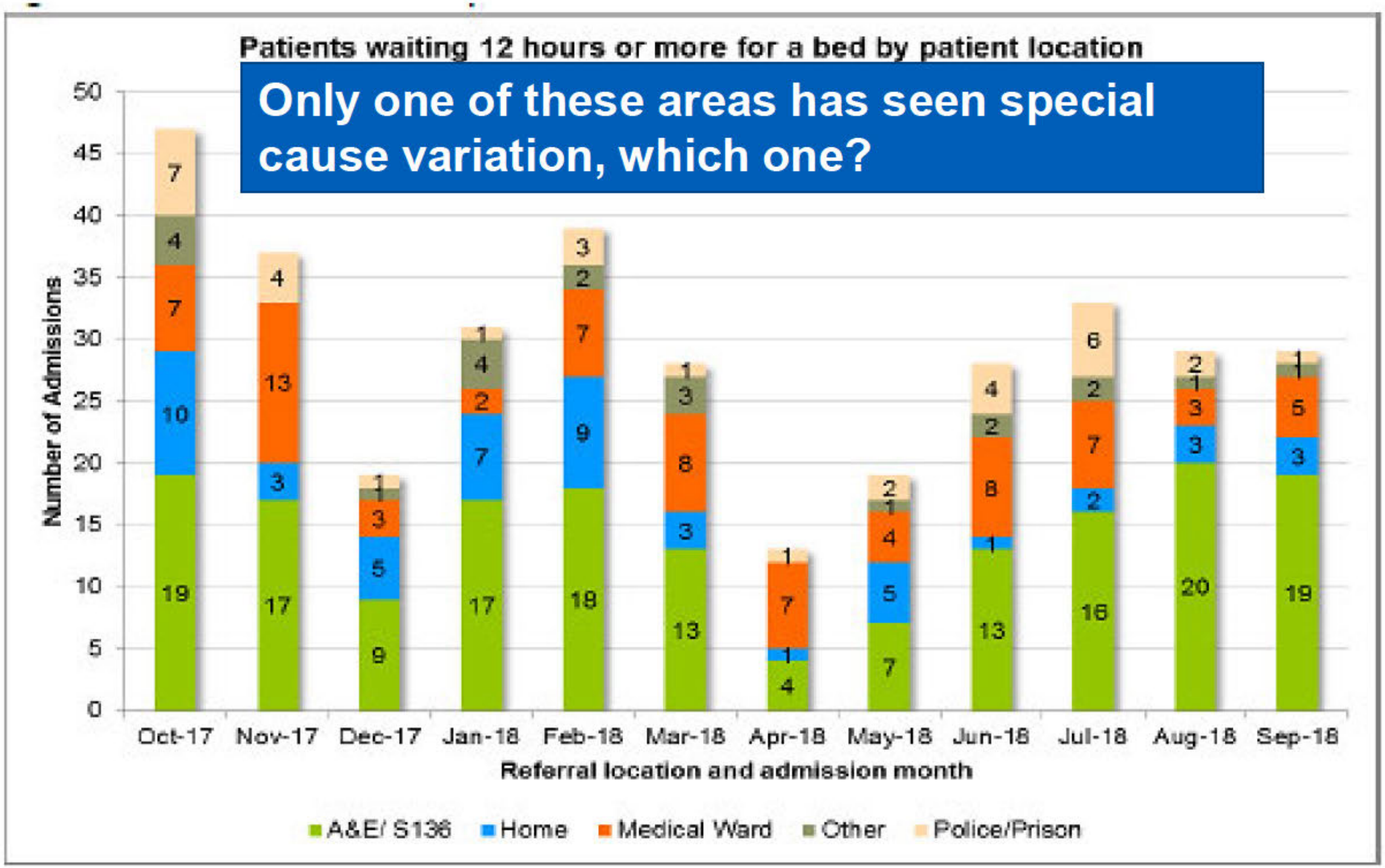

Was it green?

NHS Improvement

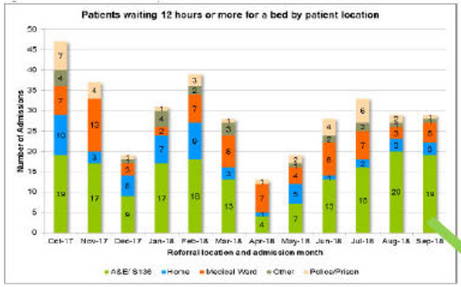

Is this significant?
Count the dots….

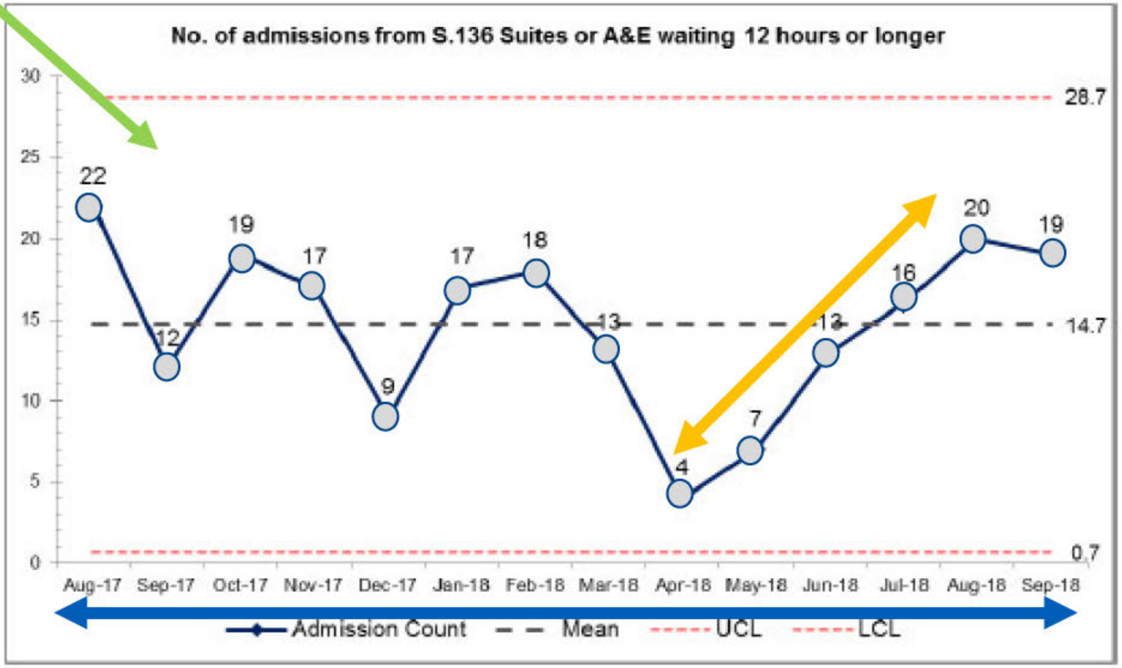

What was significant?

NHS Improvement

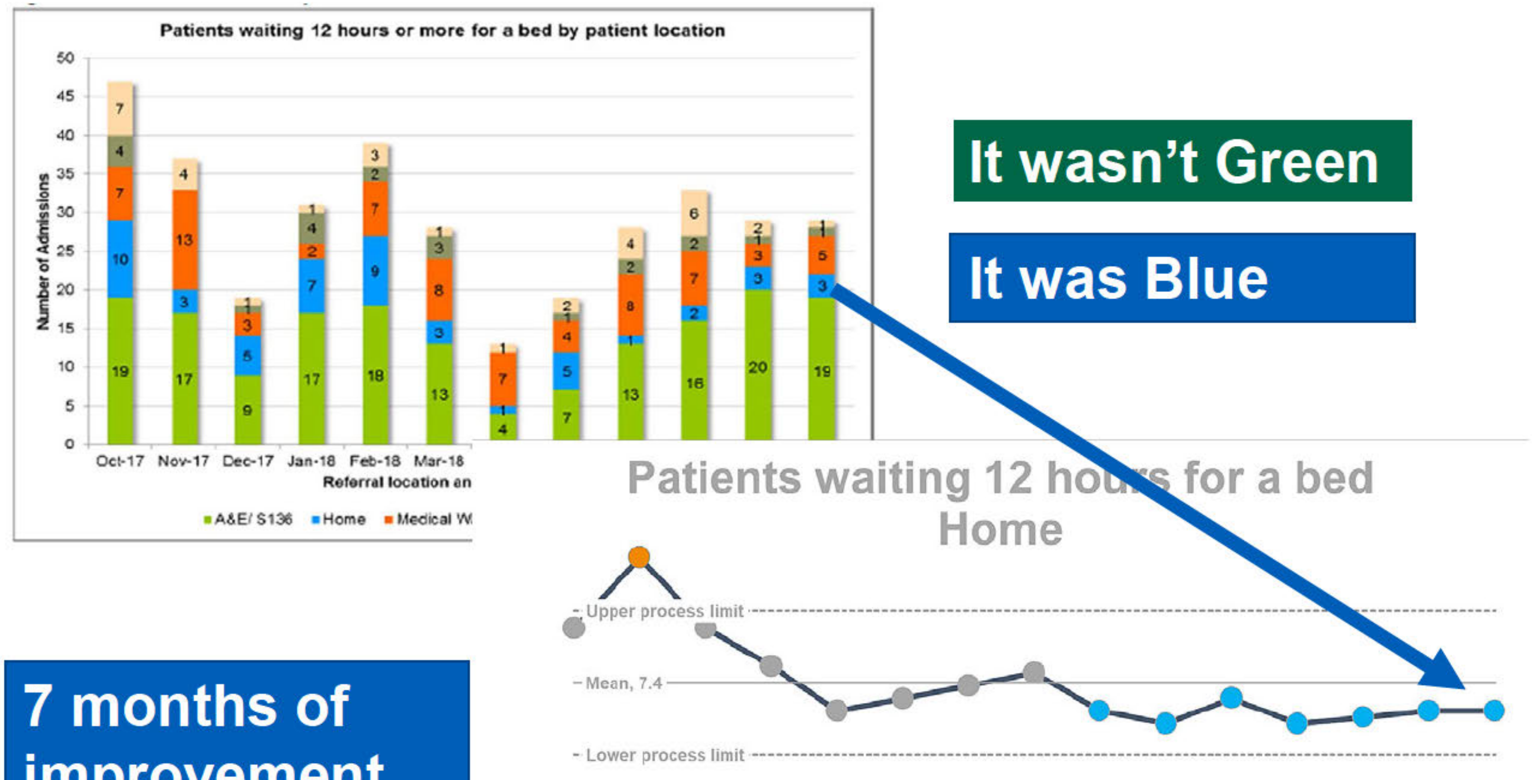

It wasn't Green

It was Blue

7 months of improvement

NHS Improvement

Are things improving?

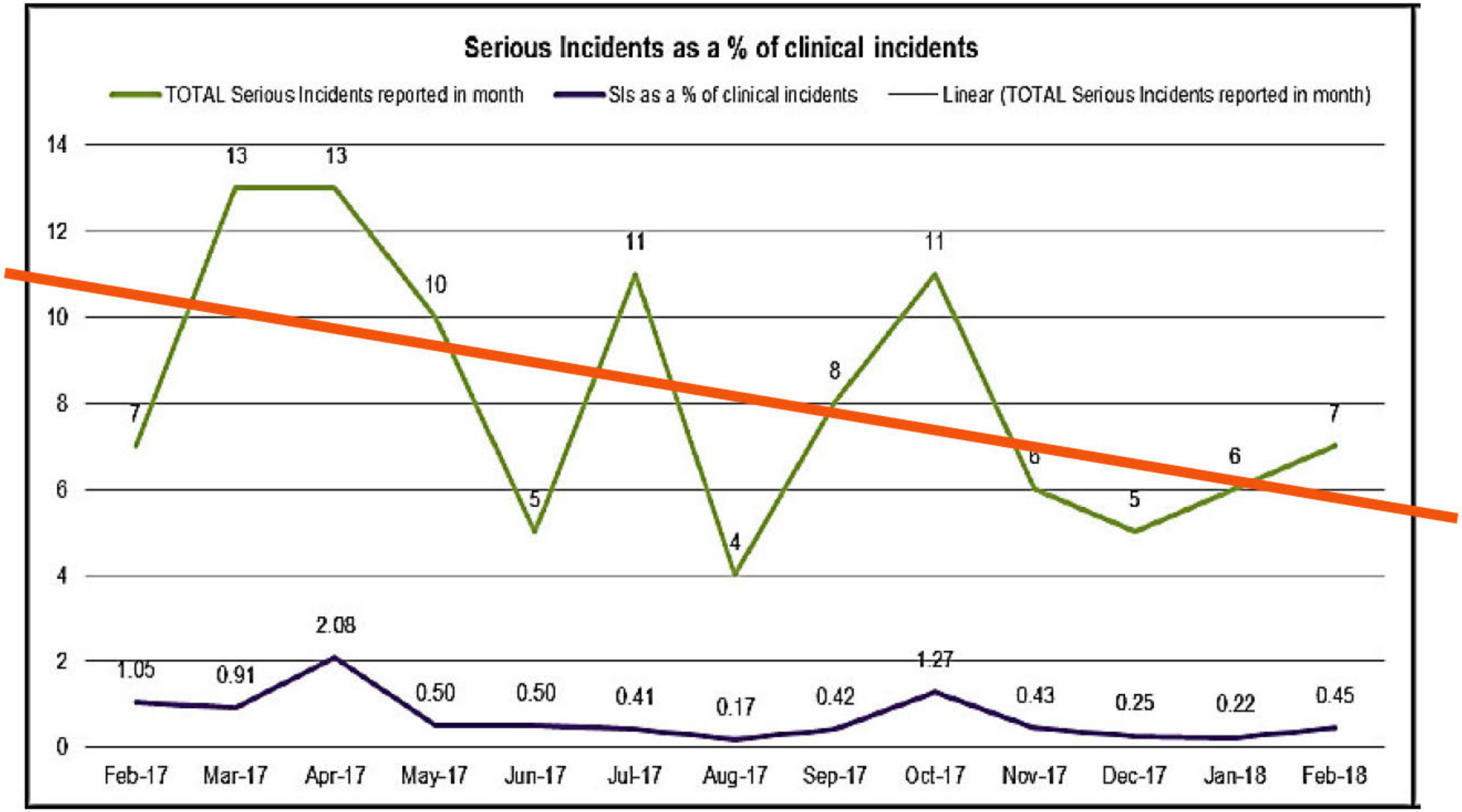

20 serious incidents a month acceptable?

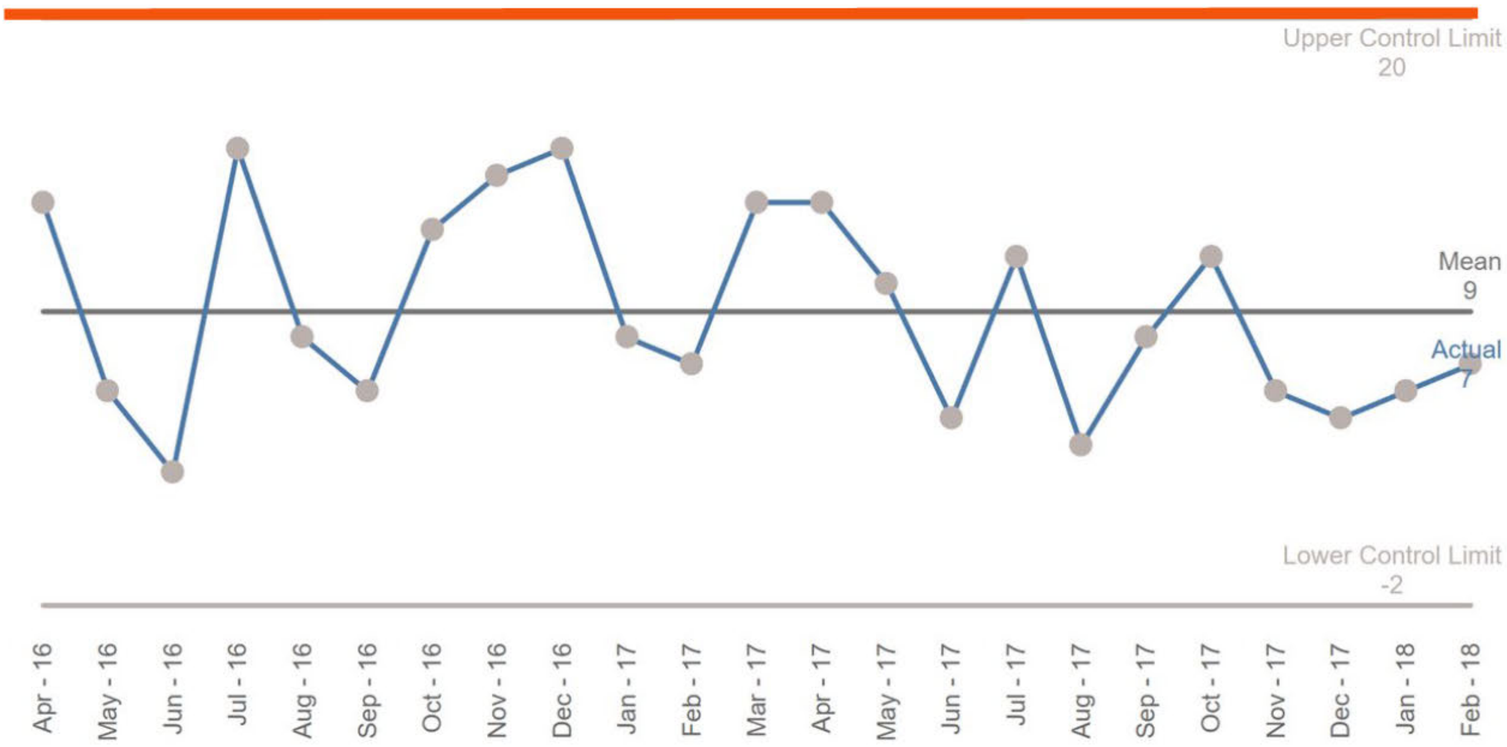

43 | Making data count

NHS Improvement

Changes being made at Avon

Key F
Gate
Dela

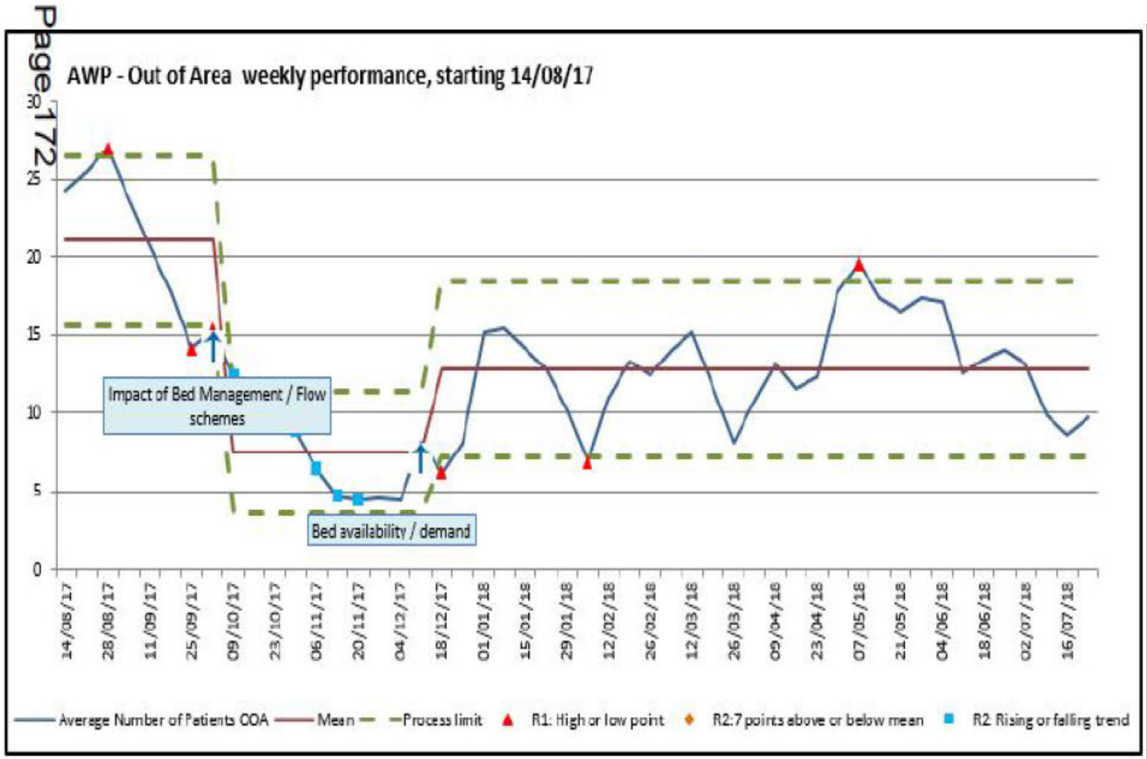

The table to the left highlights the levels of OOA placements on a weekly basis over the last 50 weeks (updated) and highlights the initial drop from 20+ down to a level of circa 7 for a 2 month period before the advent of winter and bed closures shifted the average back up to 13. Although numbers have dropped over the last three weeks of the period (below the 10 beds originally budgeted for), this doesn't yet represent a sustained shift in activity levels.

NHS Improvement

Dorset Healthcare

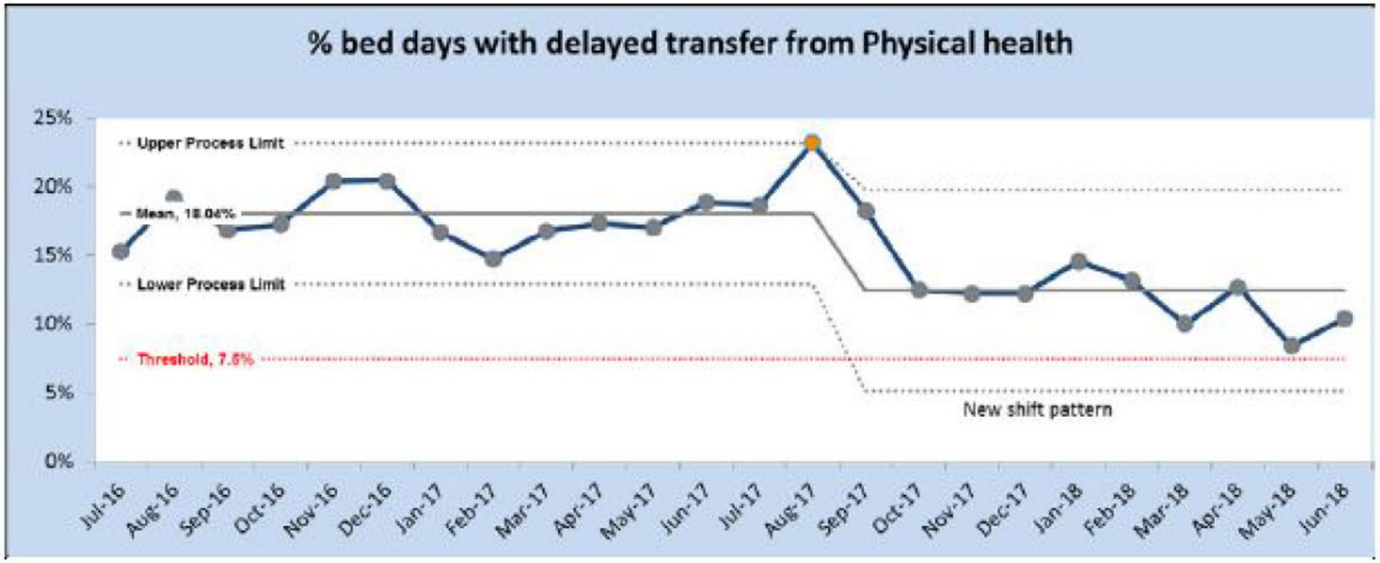

A new shift pattern was introduced in September 2017 and this improved the average DToC performance. However, SPC analysis shows that as the mean is 12.4% and the data is predicted to vary between 5.1% and 19.8% the Trust is unlikely to consistently achieve the threshold. Progress sheet 2.2.2 details improvement actions being taken.

https://www.youtube.com/watch?v=tHUMLtlJxGw

NHS Improvement

Sussex Partnership Trust

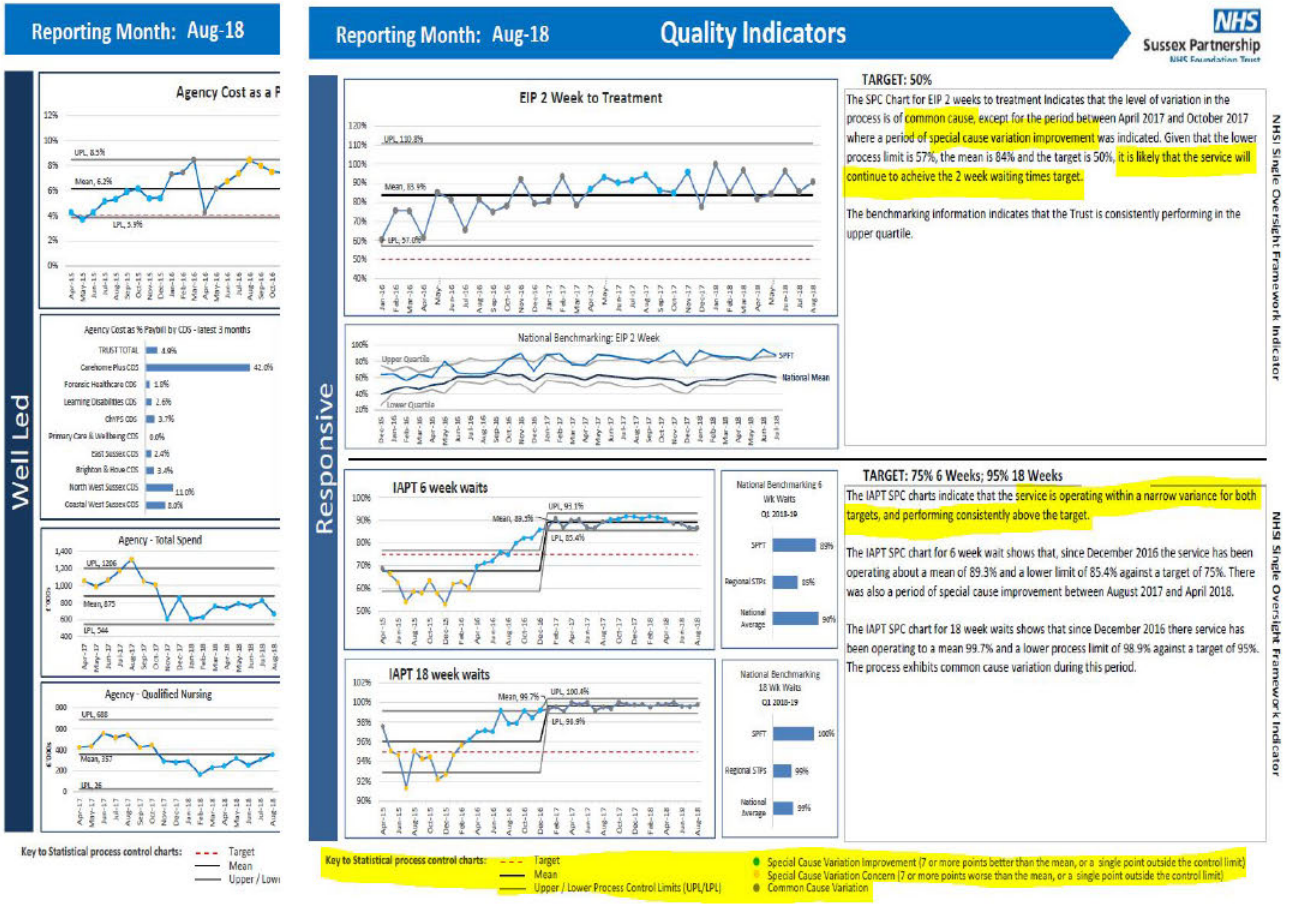

https://www.sussexpartnership.nhs.uk/sites/default/files/documents/v4_final_papers_-_public_board_of_directors_-_26_september_2018.pdf

NHS Improvement

Alternative summary report

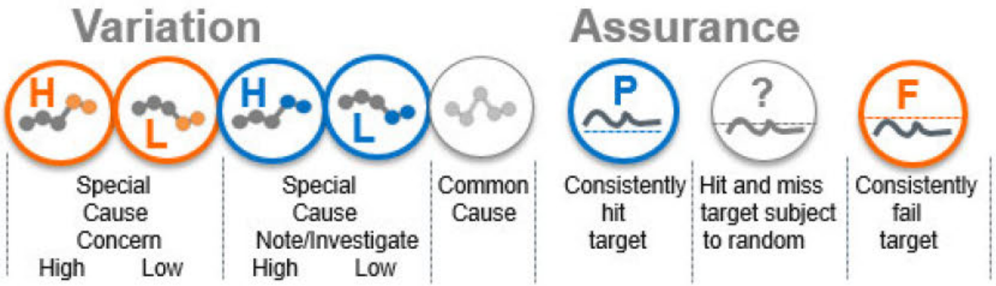

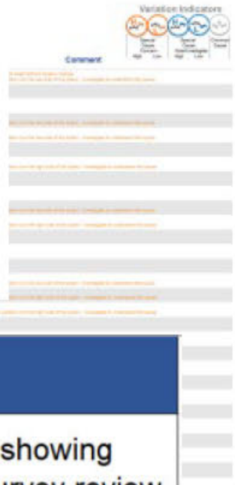

Single line indicator w[illegible] automated decision

	Jun-18	Target	Variation	Target Capability	Comment
Staff Sickness absence	4.4%	3.5%		F	Shift change in August 2017 showing increase in sickness - staff survey review indicated…..

NHS Improvement

What could good look like?

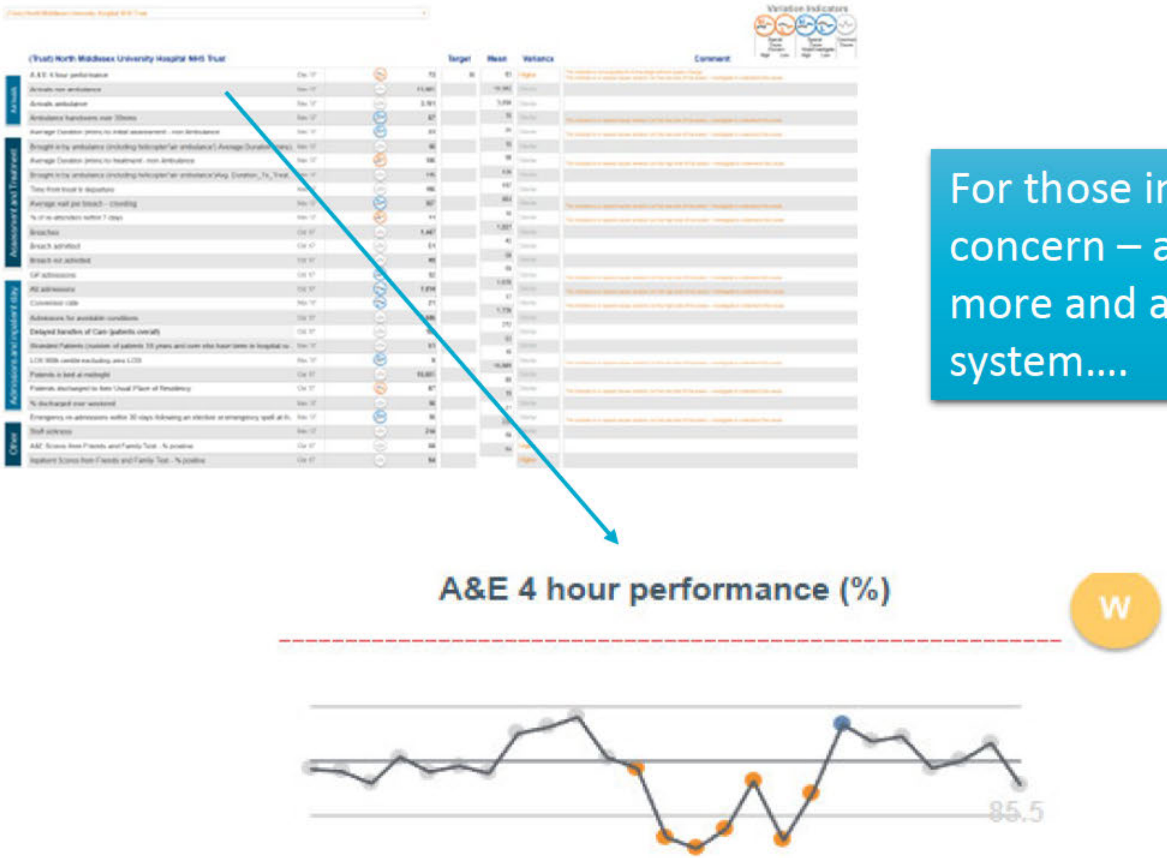

For those indicators that cause concern – ability to find out more and ask questions of the system....

NHS Improvement

SPC SOF dashboard

Operational performance	A&E performance	July 2018	87.4	95.0	84.1	88.8	93.5	Common cause variation which is the type of variation expected
	A&E Quarter Performance	July 2018	87.4	95.0	85.3	88.1	91.0	Common cause variation which is the type of variation expected
	Cancer GP Performance	June 2018	78.9		73.9	83.7	93.6	Common cause variation which is the type of variation expected
	Cancer NHS Performance	June 2018	68.8		65.3	89.6	113.9	Common cause variation which is the type of variation expected
	Diagnostic Performance	June 2018	0.8		-0.5	0.3	1.0	Special cause variation (on the high side of the scale) - investigate t understand the cause
	RTT Performance	June 2018	90.2	92.0	88.9	90.6	92.2	Concerning special cause variation (on the low side of the scale) - investigate to understand the cause
	Cdiff - Infection Rate	June 2018	19.5	0.0	13.5	15.9	18.3	Concerning special cause variation (on the high side of the scale) - investigate to understand the cause
	Cdiff - Variance Plan	June 2018	-1.0		-4.2	1.2	6.6	Common cause variation which is the type of variation expected
	FFT - A&E	[illegible] 2018	[illegible]		[illegible]	[illegible]	[illegible]	Common cause variation which is the type of variation expected
Quality of care	FFT - Community	June 2018	95.9		89.1	96.3	103.5	Common cause variation which is the type of variation expected
	FFT - Inpatient	June 2018	94.9		89.3	95.0	100.6	Common cause variation which is the type of variation expected
	FFT - Maternity Qtr2	June 2018	97.6		92.9	97.3	101.7	Common cause variation which is the type of variation expected
	FFT - Staff	March 2018	70.8		68.6	76.1	83.6	Common cause variation which is the type of variation expected
	MRSA - InfectionRate	March 2018	0.7		0.6	0.9	1.1	Special cause variation (on the low side of the scale) - investigate to understand the cause
	MSSA	June 2018	7.5		7.1	8.5	9.9	Special cause variation (on the low side of the scale) - investigate to understand the cause
	NRLS - UnderReporting	May 2018	46.2		23.9	35.2	46.4	Special cause variation (on the high side of the scale) - investigate t understand the cause
	VTE - Risk	March 2018	1.0		0.9	1.0	1.0	Special cause variation (on the high side of the scale) - investigate t understand the cause
...rship ...d ...ement ...bility	Workforce - Staff Sickness	March 2018	3.8		3.1	3.6	4.2	Concerning special cause variation (on the high side of the scale) - investigate to understand the cause

RTT Performance June 2018 90.2 92.0

NHS Improvement

Tools

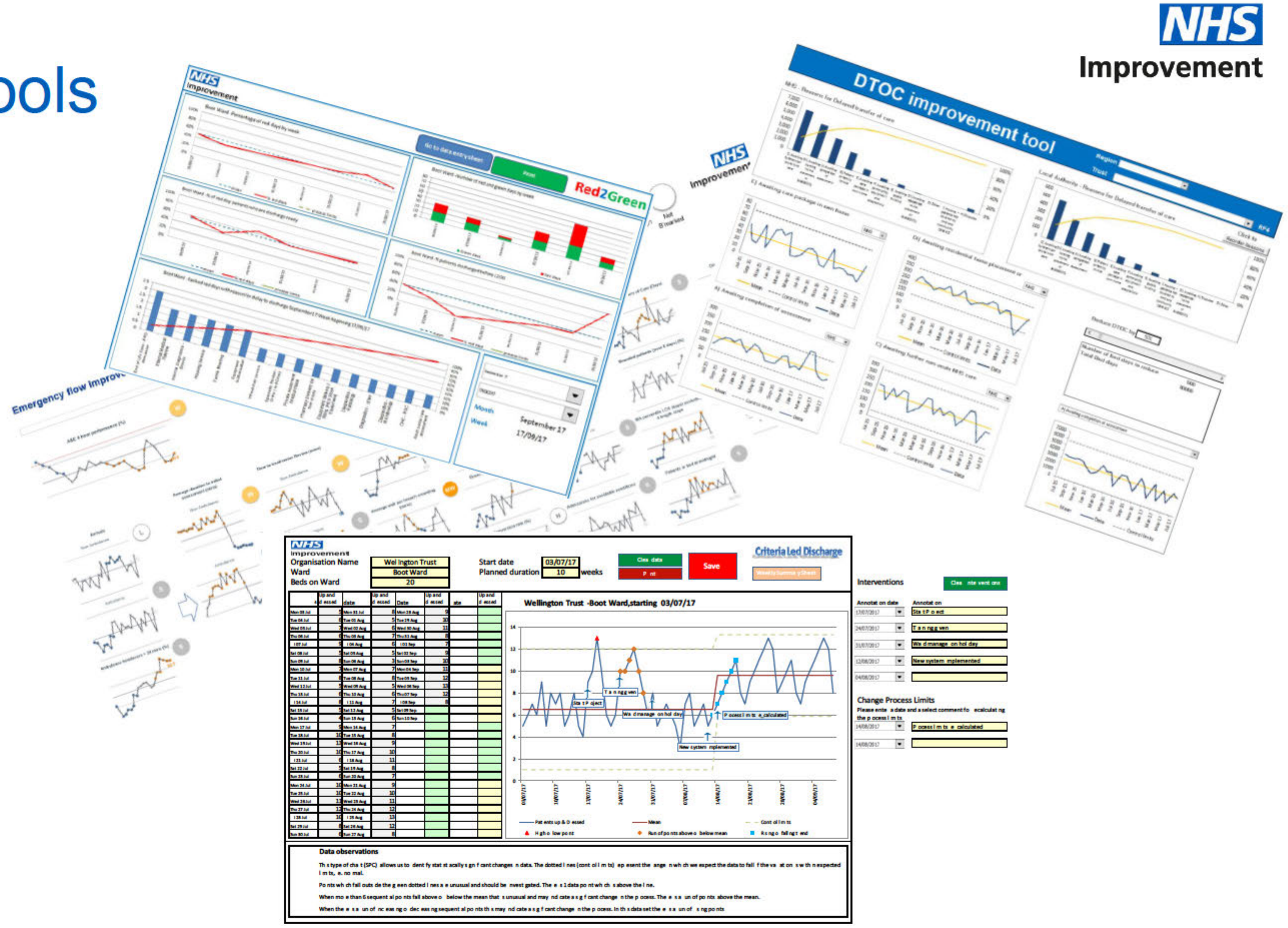

Free SPC tool

NHS Improvement

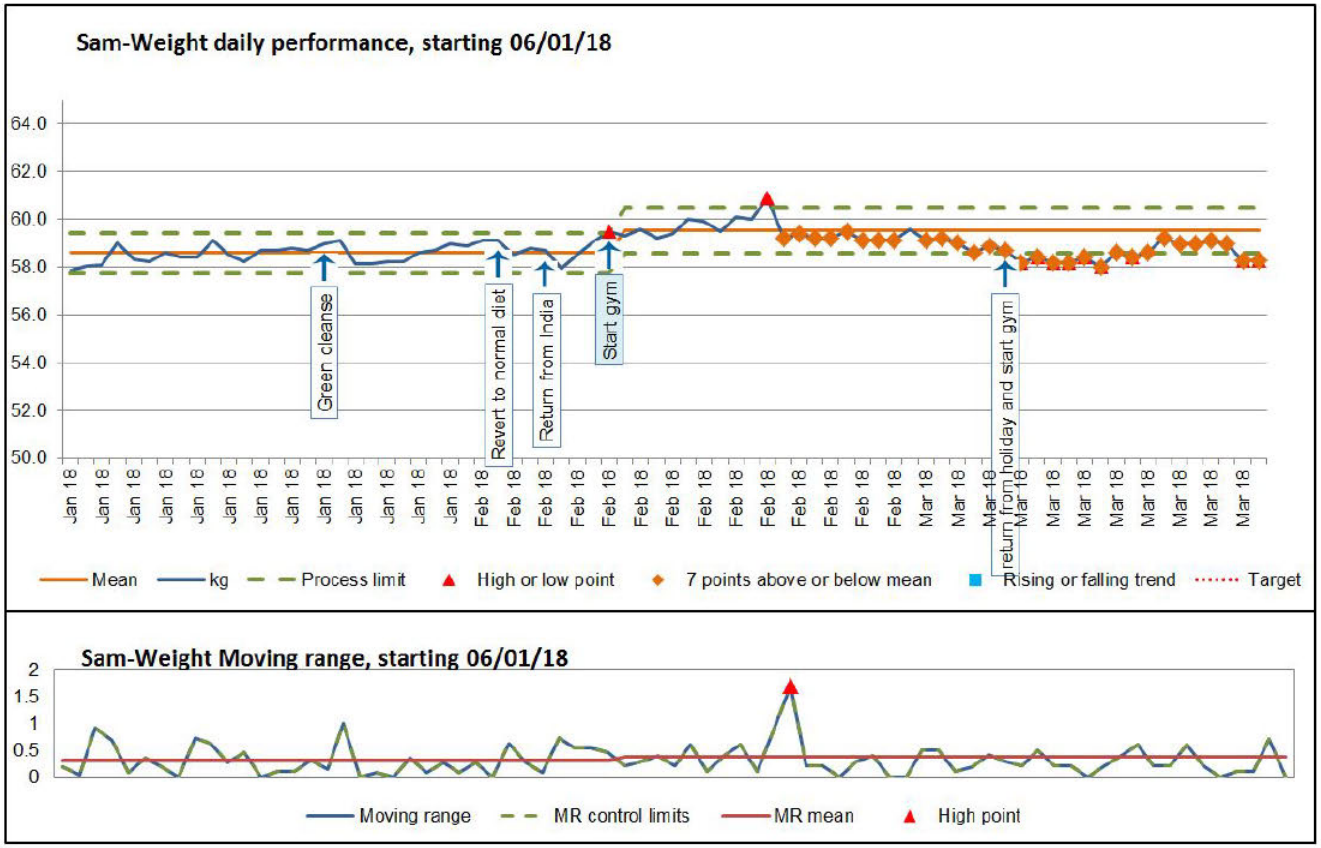

https://improvement.nhs.uk/resources/making-data-count/

NHS Improvement

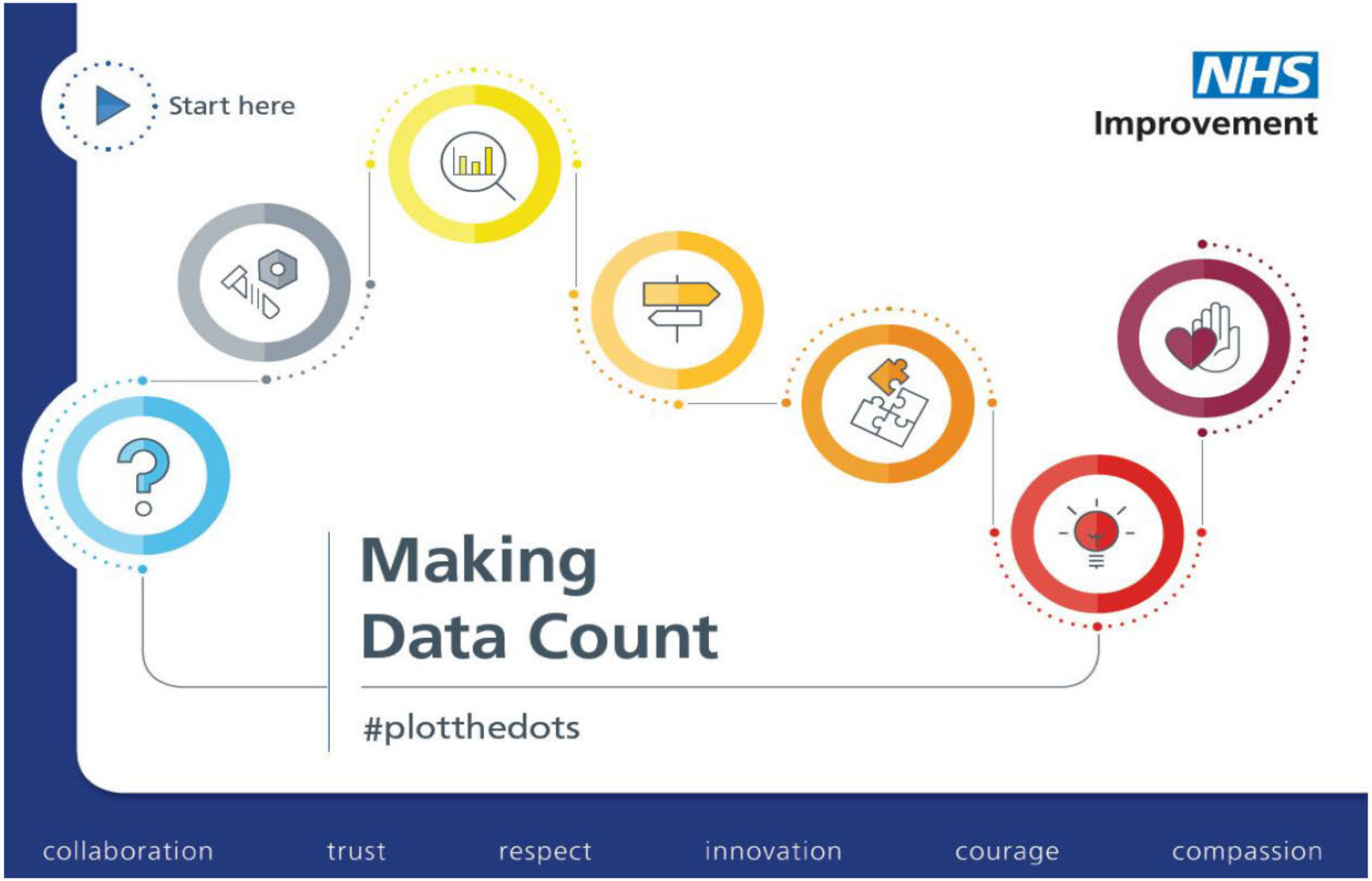

52 | Making data count

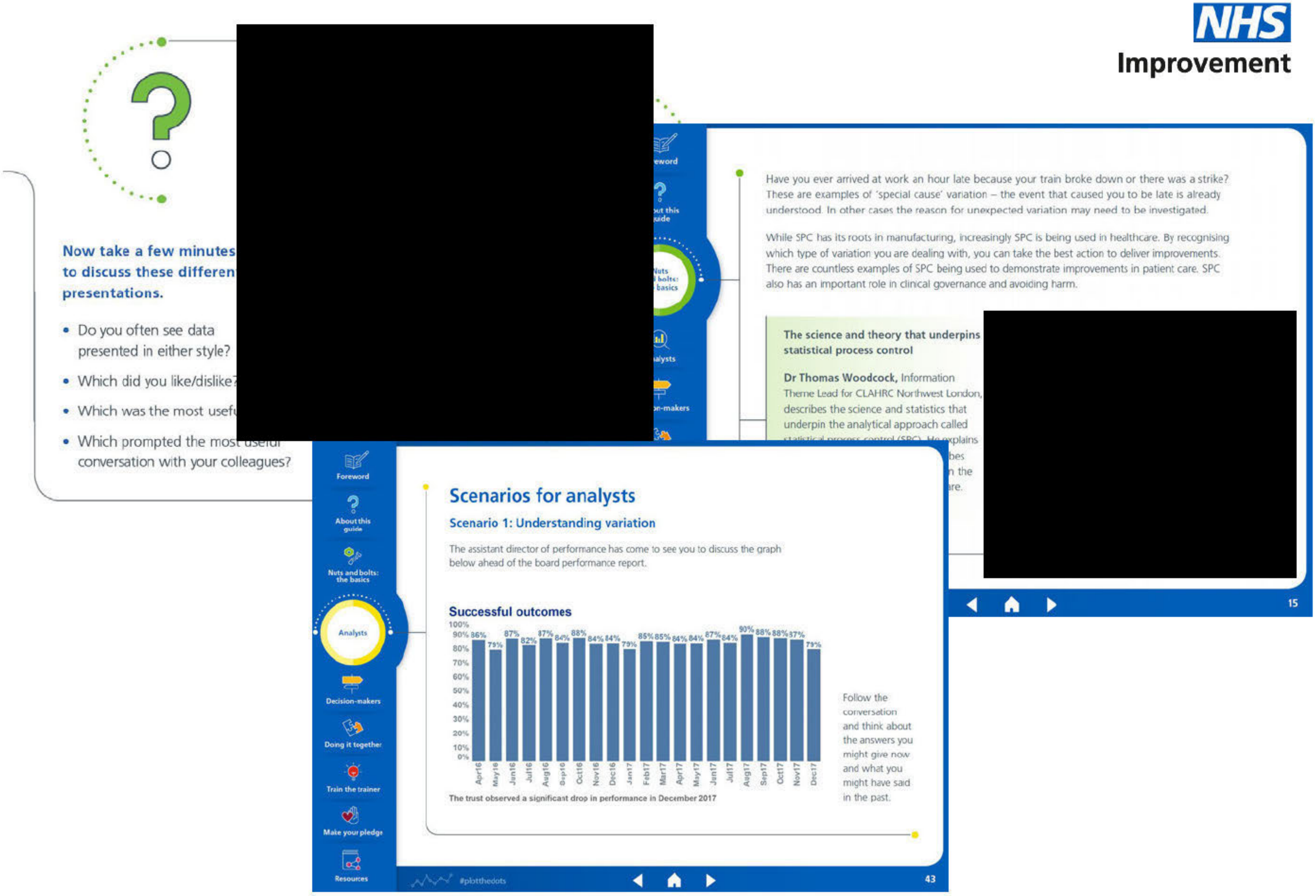
NHS
Improvement
Now take a few minutes to discuss these differen[redacted]
presentations.
• Do you often see data presented in either style?
• Which did you like/dislike?
• Which was the most usef[redacted]
• Which prompted the most [redacted] conversation with your colleagues?
Have you ever arrived at work an hour late because your train broke down or there was a strike? These are examples of 'special cause' variation – the event that caused you to be late is already understood. In other cases the reason for unexpected variation may need to be investigated.
While SPC has its roots in manufacturing, increasingly SPC is being used in healthcare. By recognising which type of variation you are dealing with, you can take the best action to deliver improvements. There are countless examples of SPC being used to demonstrate improvements in patient care. SPC also has an important role in clinical governance and avoiding harm.
The science and theory that underpins statistical process control
Dr Thomas Woodcock, Information Theme Lead for CLAHRC Northwest London, describes the science and statistics that underpin the analytical approach called
[redacted]
15
Foreword
About this guide
Nuts and bolts: the basics
Analysts
Decision-makers
Doing it together
Train the trainer
Make your pledge
Resources
Scenarios for analysts
Scenario 1: Understanding variation
The assistant director of performance has come to see you to discuss the graph below ahead of the board performance report.
Successful outcomes
The trust observed a significant drop in performance in December 2017
Follow the conversation and think about the answers you might give now and what you might have said in the past.
#plotthedots
43

Pledges for action

[redacted]

Making Data Count network

NHS Improvement

To register go to:
https://www.source4networks.org.uk

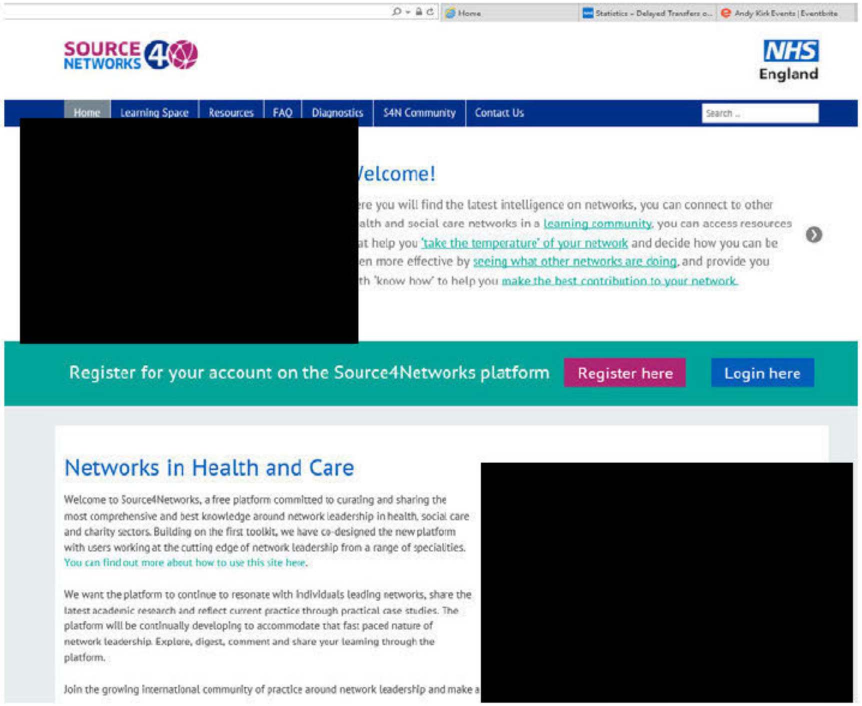

NHS
Improvement

SPC has provoked new questions & made us realise the key issues that we should be discussing

Huge added value – a game changer

All Trusts should do this. It's like switching the light on so you can see the data

NHS
Improvement

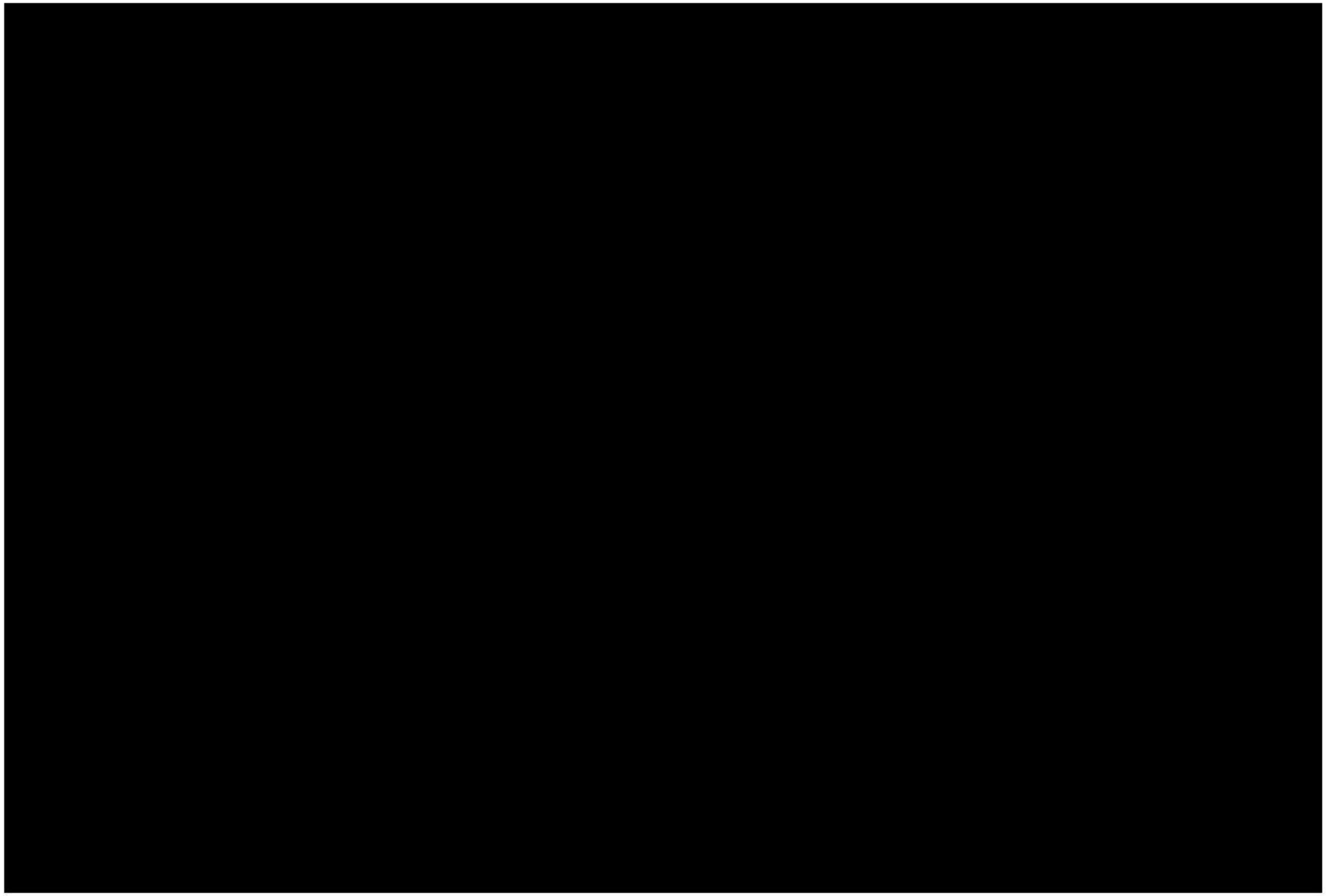

NHS
Improvement

[redacted]

58 | Presentation title
