# Supplementary material for: Retrospective evaluation of an intervention based on training sessions to increase the use of control charts in hospitals
Source: BMJ Qual Saf. 2022 Jun 24;32(2):100–8. doi: 10.1136/bmjqs-2021-013514 (PMC9887349; doi:10.1136/bmjqs-2021-013514)
Supplement: Supplementary data [file bmjqs-2021-013514supp005.pdf]

BMJ Publishing Group Limited (BMJ) disclaims all liability and responsibility arising from any reliance placed on this supplemental material which has been supplied by the author(s)

Supplementary 5 - Making Data Count Powerpoint (2)

NHS Improvement

Making data count

– the why, the how and the experience so far

6th July 2018

Samantha Riley, Head of Improvement Analytics, NHS Improvement

Mark Outhwaite, Non Exec Director, Avon & Wiltshire Mental Health Partnership NHS Trust

collaboration trust respect innovation courage compassion

NHS Improvement

Where are we now?

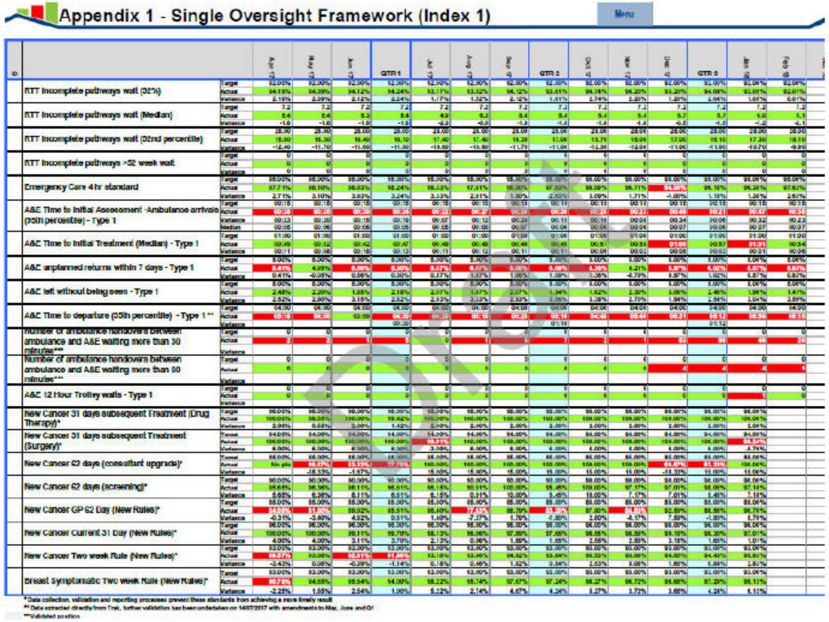

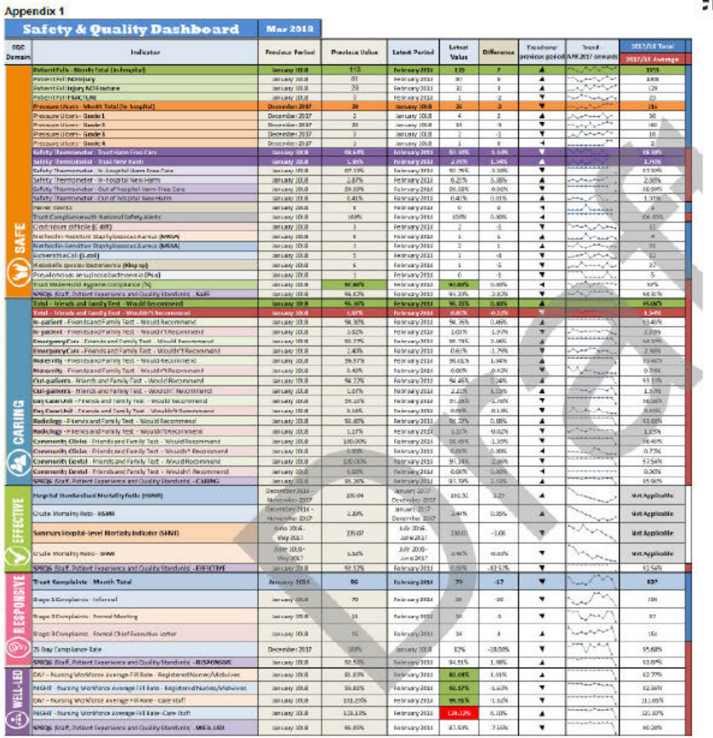

NHS Improvement

Where are we now?

Safety & Quality Dashboard		Mar 2018							
CQC Domain	Indicator	Previous Period	Previous Value	Latest Period	Latest Value	Difference	Trend over previous period	Trend - APR 2017 onwards	2017/18 Total / 2017/18 Average
	Emergency Care - Friends and Family Test - Would Recommend	January 2018	93.27%	February 2018	95.73%	2.46%	▲		94.32%

Trend over previous period

▲

One month trend.......

Is an increase from 95.36% to 95.76% important or distracting narrative?

Caring

7 Family and Friends Test (FFT) (data up to February 2018)

7.2 The Trusts 'Would Recommend' for Friends and Family returns *increased* to 95.76% for February 2018 from 95.36% in January 2018. The percentage of patients who stated they 'Wouldn't Recommend' *decreased* to 0.85% in February 2018 from 1.07% in January 2018.

NHS Improvement

Poll 1

What best describes your current integrated performance for the board :

- Mainly RAG charts
- A mixture of RAG and time series data/spark lines
- Presence of SPC charts

collaboration trust respect innovation courage compassion

NHS Improvement

Improving Access to Psychological Therapies – performance against target

Metric	Target	Jan-17	Feb-17	Mar-17	Apr-17	May-17	Jun-17	Jul-17	Aug-17
IAPT Treatment 18 weeks	95%	99.8%	99.5%	99.9%	99.8%	99.4%	99.7%	99.6%	99.7%
IAPT Treatment 6 weeks	75%	86.3%	84.1%	83.3%	80.9%	74.9%	79.5%	81.1%	81.2%
IAPT Recovery Rate	50%	59.3%	57.0%	54.0%	55.3%	53.6%	52.2%	55.3%	54.8%
EIS First Episode Psychosis	50%	100.0%	100.0%	83.0%	62.5%	100.0%	89.5%	100.0%	85.0%

NHS Improvement

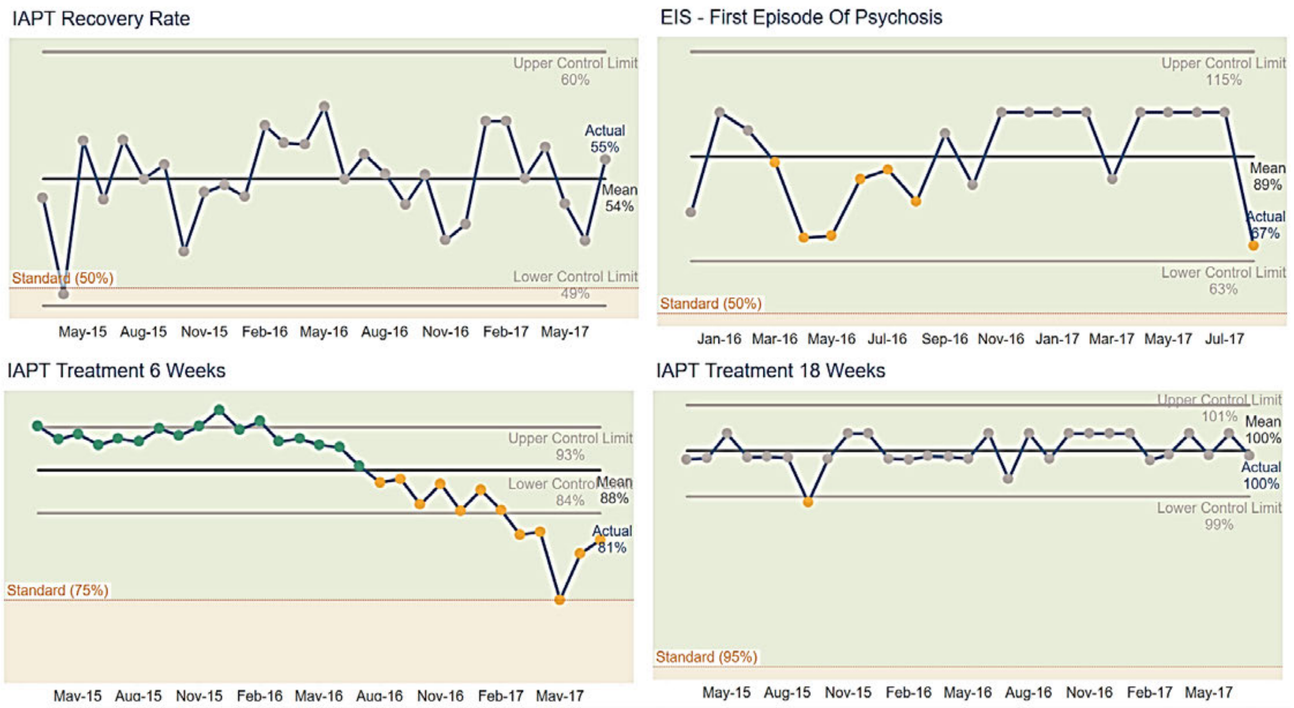

BMJ Publishing Group Limited (BMJ) disclaims all liability and responsibility arising from any reliance placed on this supplemental material which has been supplied by the author(s)

Did green provide true assurance?

NHS Improvement

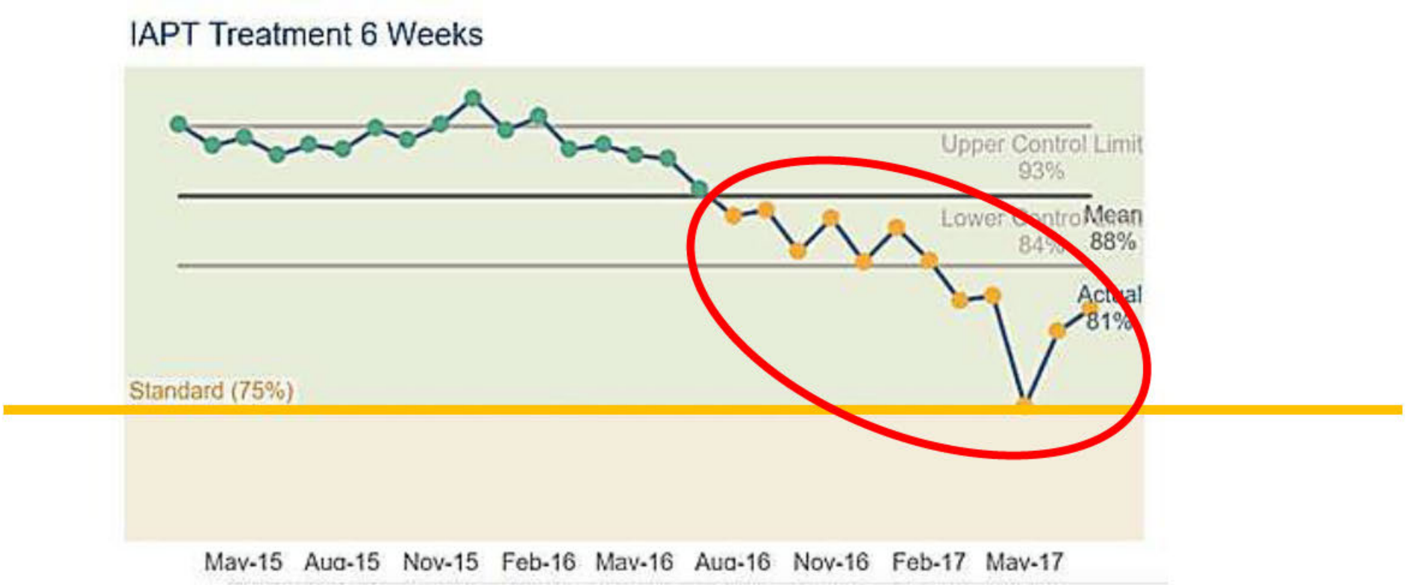

7 | Making data count

Scenario

Improvement

We’re going to simulate some **real data** in a healthcare setting

We’ll be thinking about **how people react to patterns and trends** in data.

Can you spot an **improvement or decline** when it occurs? We’ll begin plotting our data in a **run chart.**

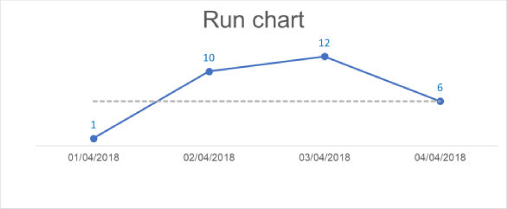

Kudrna L, *et al. BMJ Qual Saf* 2023; 32:100–108. doi: 10.1136/bmjqs-2021-013514

Reducing serious incidents

NHS Improvement

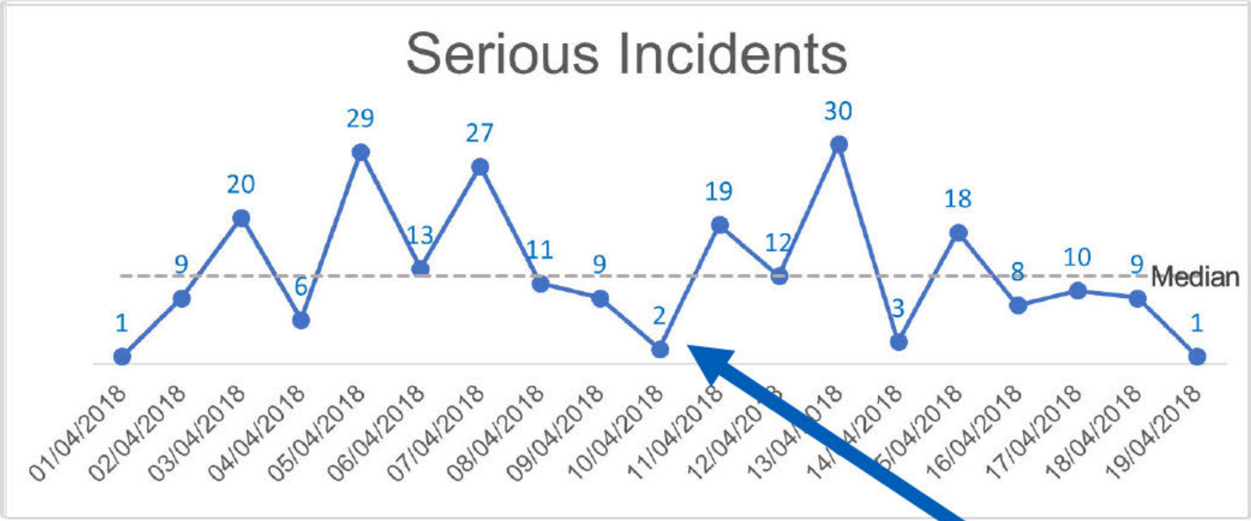

Has the improvement idea been successful?

Are you worried you might have seen this pattern before?

BMJ Publishing Group Limited (BMJ) disclaims all liability and responsibility arising from any reliance placed on this supplemental material which has been supplied by the author(s)

Improvement idea

NHS Improvement

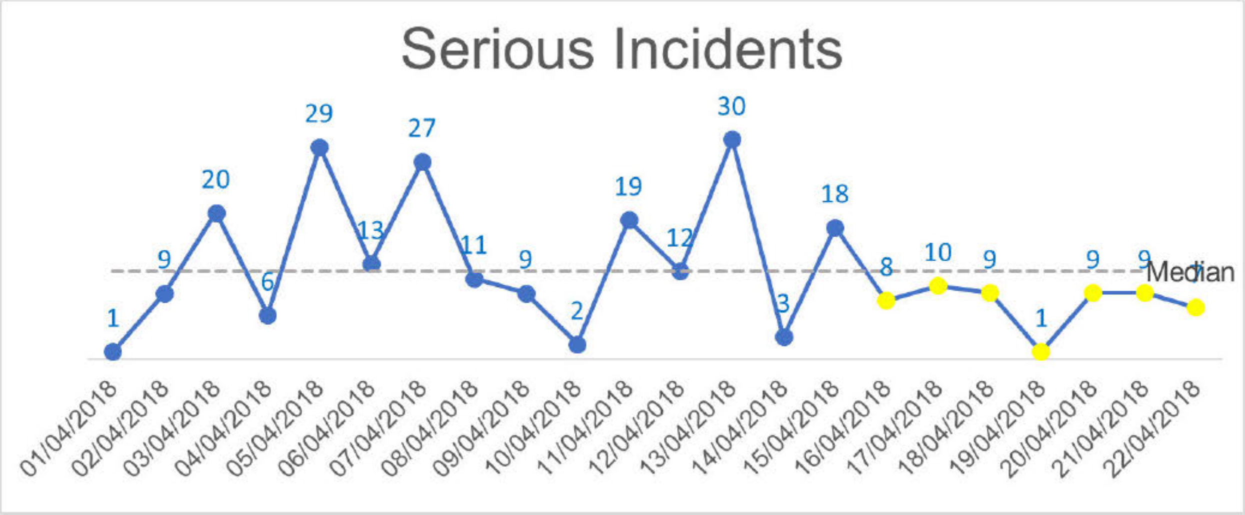

Now seven days below the baseline median…

We could go on… when should we recognise a trend?

Kudrna L, *et al. BMJ Qual Saf* 2023; 32:100–108. doi: 10.1136/bmjqs-2021-013514

The data that created this scenario

NHS Improvement

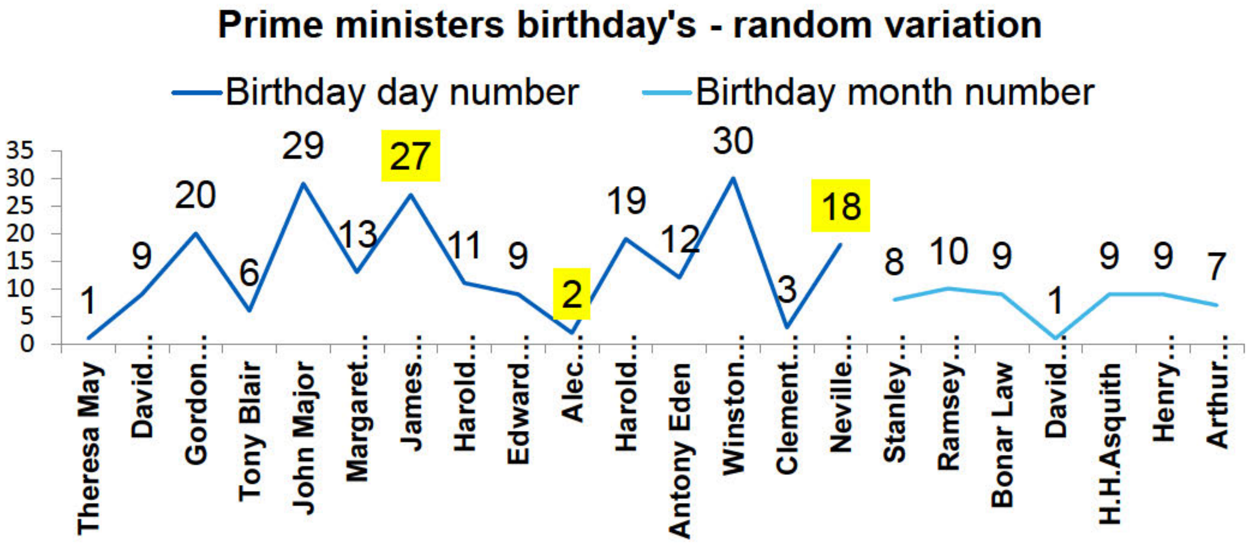

Any patterns at these points were randomly generated, then I changed the rules of the scenario….

NHS Improvement

Anatomy of a SPC chart

Time series line chart with 3 reference lines

20 plus data points for a robust analysis

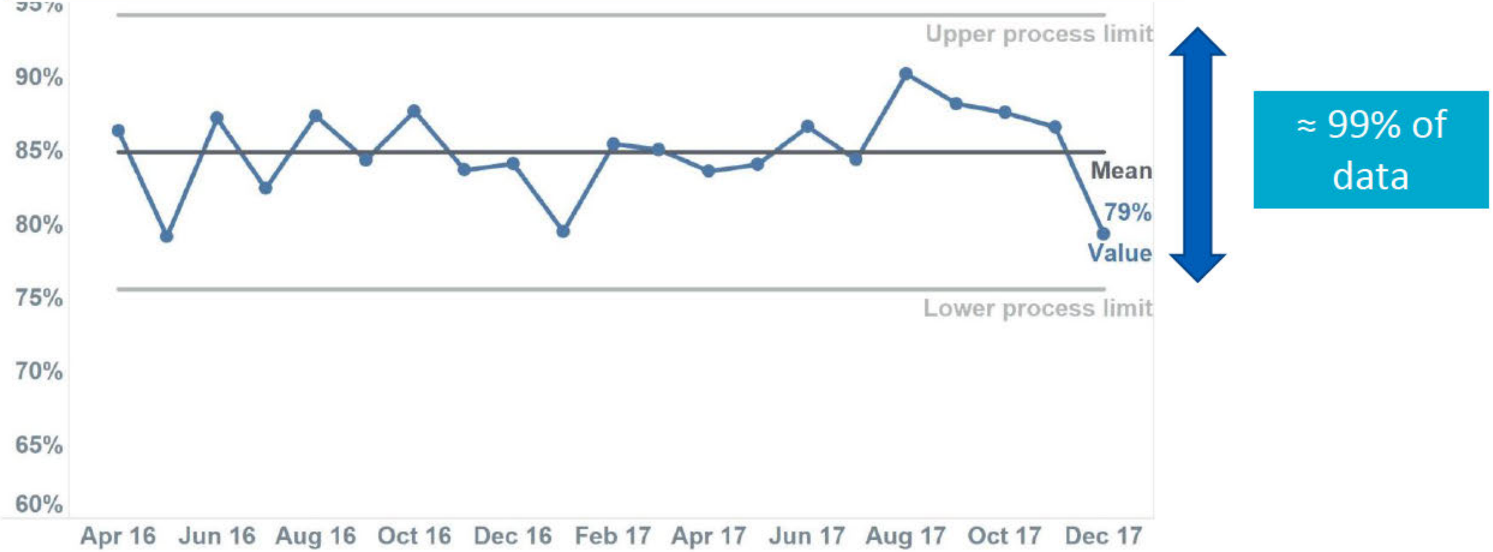

SPC rules

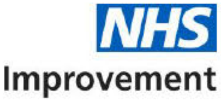
NHS
Improvement

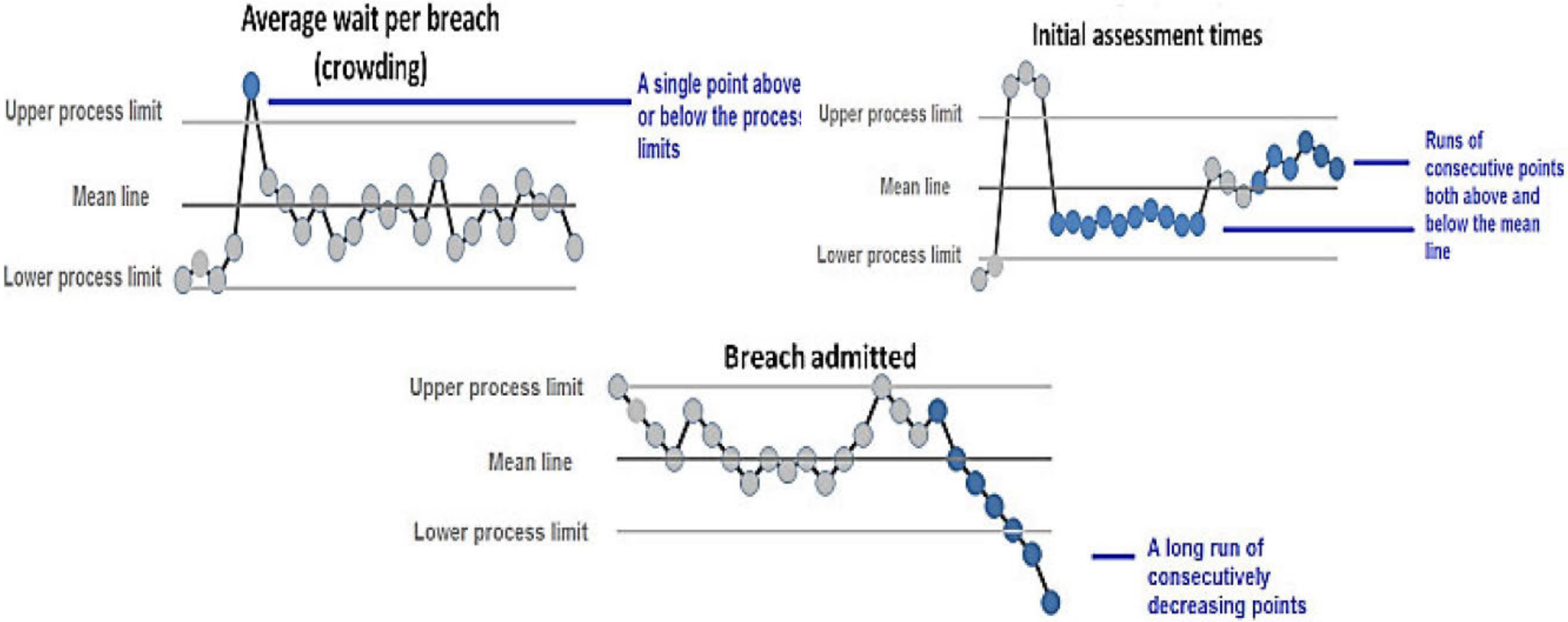
Average wait per breach
(crowding)
Upper process limit
Mean line
Lower process limit
A single point above
or below the process
limits
Initial assessment times
Upper process limit
Mean line
Lower process limit
Runs of
consecutive points
both above and
below the mean
line
Breach admitted
Upper process limit
Mean line
Lower process limit
A long run of
consecutively
decreasing points

BMJ Publishing Group Limited (BMJ) disclaims all liability and responsibility arising from any reliance placed on this supplemental material which has been supplied by the author(s)

Why is 7 significant?

[redacted]

A trend of 2 has the probability of 25% occurrence (**one in four**)

A trend of 4 has the probability of 6.25% occurrence (**one in sixteen**)

A trend of 7 has the probability of 0.8% occurrence (**one in one hundred and twenty-eight**)

Kudrna L, *et al. BMJ Qual Saf* 2023; 32:100–108. doi: 10.1136/bmjqs-2021-013514

NHS Improvement

Evidence base

Public health

Bristol, Shipman, and clinical governance: Shewhart's forgotten lessons

Mohammed A Mohammed, K K Cheng, Andrew Rouse, Tom Marshall

During the past century, manufacturing industry has achieved great success in improving the quality of its products. An essential factor in this success has been the use of Walter A Shewhart's pioneering work in the economic control of variation, which culminated in the development of a simple yet powerful graphical method known as the control chart. This chart classifies variation as having a common cause or special cause and thus guides the user to the most appropriate action to effect improvement. Using six case studies, including the excess deaths after paediatric cardiac surgery seen in Bristol, UK, and the activities of general practitioner turned murderer Harold Shipman, we show a central role for Shewhart's approach in turning the rhetoric of clinical governance into a reality.

During the past century, manufacturing industry has achieved great success in improving the quality of its products. In industry, the definition of quality is "on target with minimum variation".[1] Reduction of variation is also a core concern in clinical governance;[2] however, there are fundamental and profound differences between the ways in which health services and industry make sense of variation. We begin with an illustration of the industrial approach to understanding and controlling variation, followed by application of this approach to health care, using six clinical governance case studies: mortality rates after paediatric cardiac surgery in Bristol, UK; mortality rates in older women treated by the general practitioner and convicted serial killer Harold Shipman; success rates of in-vitro fertilisation (IVF) treatment; neonatal deaths; prevalence of coronary heart disease in primary care; and mortality after fractured neck of femur.

Common-cause and special-cause variation

Consider a process such as writing a signature. Five of MAM's signatures are shown in the left of figure 1. Although these signatures were produced under the same conditions and by the same process, they are not identical. However, although they show variation, the signatures on the left are identical. No signature is better or worse than the others. If we want to reduce the variation between signatures, we must change the way we write all signatures, not just the ones that fail an adequate test. Thus, conventional approaches to understanding variation from a stable system can misguide us to act on individual failures rather than acting on the underlying process.

Now consider the sixth signature, on the right. It is clearly different from the others. A casual look suggests that there must be a special reason why this is so. If we want to address this kind of variation, we need to identify this special cause and prevent it from interacting with an otherwise stable process. (In this case, the signature is a forgery, attempted by TM under the same essential conditions!)

This approach categorises variation according to the action needed to reduce it. Common-cause variation is intrinsic to the process. To decrease common-cause variation, we need to act on the process. Special-cause variation is the result of factors extrinsic to the process, and its reduction therefore requires identification of and action on the special causes. The originator of these fundamental concepts was a physicist and engineer—Walter A Shewhart.[3] His pioneering work at

Downloaded from [illegible]

THE PROBLEM WITH...

The problem with red, amber, green: the need to avoid distraction by random variation in organisational performance measures

Jacob Anhøj, Anne-Marie Blok Hellesøe

Centre for Diagnostic Investigatin, Rigshospitalet, University of Copenhagen, Copenhagen, Denmark

Correspondence to
Dr Jacob Anhøj, Centre for Diagnostic Investigatin, Rigshospitalet, University of Copenhagen, Blegdamsvej 9, Copenhagen 2100, Denmark; jacob@anhoej.net

Accepted 18 January 2016
Published Online First 31 March 2016

'The Problem with...' series covers controversial topics related to efforts to improve healthcare quality, including widely recommended but deceptively difficult strategies for improvement and pervasive problems that seem to resist solution.

INTRODUCTION

Many healthcare organisations now track a number of performance measures like infection and complication rates, waiting times, staff adherence to guidelines, etc. Our own organisation, The Capital Region of Denmark, provides healthcare for 1.7 million people and runs 6 hospitals and 11 mental health centres. Measures of clinical quality have been widely used in our region locally at hospitals and departments for many years. Recently, our region started to systematically define and track strategical key performance measures also at the top management level. Approximately 25 measures on a wide range of subjects from hospital infections to public transportation are being tracked by the top management and the Regional Council.

The measurement strategy for hospitals involves a bottom–up approach allowing each hospital and department to, if needed, define its own performance measures that feed into one or more of the overall measures. For example, bacteraemia is one of the overall measures, and some acute-care departments, who rarely see hospital-acquired bacteraemia, have started to work on reducing the use of bladder catheters in order to reduce the risk of bacteraemia from catheter-related urinary tract infections diagnosed after their patients have been transferred to other departments. To support their work, they have developed a handful of measures that track the use of catheters and staff compliance with standard procedures related to catheter use.

We welcome this development very much. The choice of relatively few overall measures combined with the bottom–up approach is a helpful strategy that focuses and aligns improvement work and stimulates the use of data at all levels of the organisation while leaving room for meaningful local adaptations of performance measures.

However, we do not at all welcome the widespread use of red, amber, green approaches to data analysis that is everywhere in our organisation.

By 'red, amber, green', we are referring to graphical data displays that use colour coding of individual data values based on whether this value is on the right (green) or wrong (red) side of a target value. Often amber or yellow is used to indicate data values that are somewhere between 'right' and 'wrong'.

The problem with red, amber, green management is that at best is it useless, at worst it is harmful.

THE PROBLEM WITH RED, AMBER, GREEN

Figure 1 was captured from the February 2015 report on regional performance measures. It shows the monthly count of a certain type of unwanted incident in mental healthcare. The horizontal line represents the target value of 10.5. That is, we do not want more than 10 incidents per month. Red bars show months above target. Green bars show months below target.

The data display in figure 1 is formally correct (green is better than red). However, it fails to convey a very

Linked

▸ http://dx.doi.org/10.1136/bmjqs-2015-004967
▸ http://dx.doi.org/10.1136/bmjqs-2016-005303

CrossMark

To cite: Anhøj J, Hellesøe A-MB. *BMJ Qual Saf* 2017;**26**:81–84.

CQC – signs of a mature QI approach

NHS Improvement

3. The Board looks at data as time series analysis, and makes decisions based on an understanding of variation.[1]

[1] data are presented as run or control charts, instead of bar graphs, pie charts or RAG rated. Narrative analysis describes system quality and performance using terminology of common cause and special cause variation.

https://www.cqc.org.uk/sites/default/files/20180404_9001395_briefguide-quality_improvement_healthcare_provider%20v1.pdf

16 | Making data count

If there is ‘special cause’

NHS Improvement

A single point outside the control limits

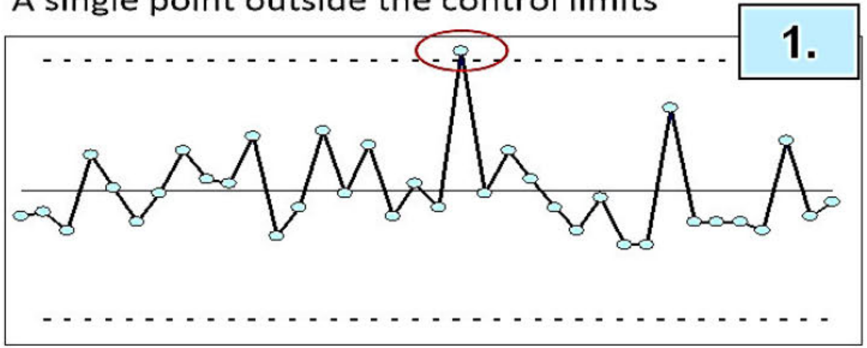

[redacted]

NHS Improvement

Unacceptable variation

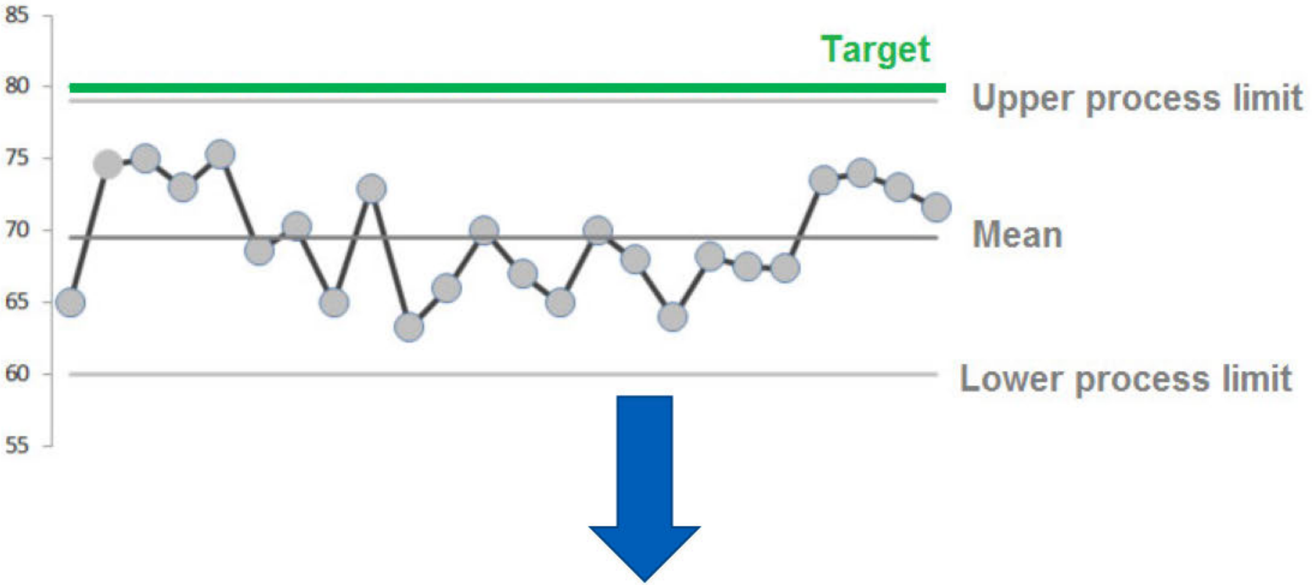

Redesign the system

18 | Making data count

NHS Improvement

Everything is failing?

										Combined Trust Performance					Trust data 13 months	
Domain	Indicator	Jul-17	Aug-17	Sep-17	2017-2018	Jul-17	Aug-17	Sep-17	2017-2018	Jul-17	Aug-17	Sep-17	2017-2018 Q2	2017-2018	Trendcharts	
Training	Mandatory training compliance (Target: >90%)	85.4%	86.1%	85.5%	84.6%	85.2%	86.5%	85.7%	85.1%	85.4%	86.2%	85.6%	85.7%	84.8%	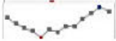	!

Presentation influences discussion

NHS Improvement

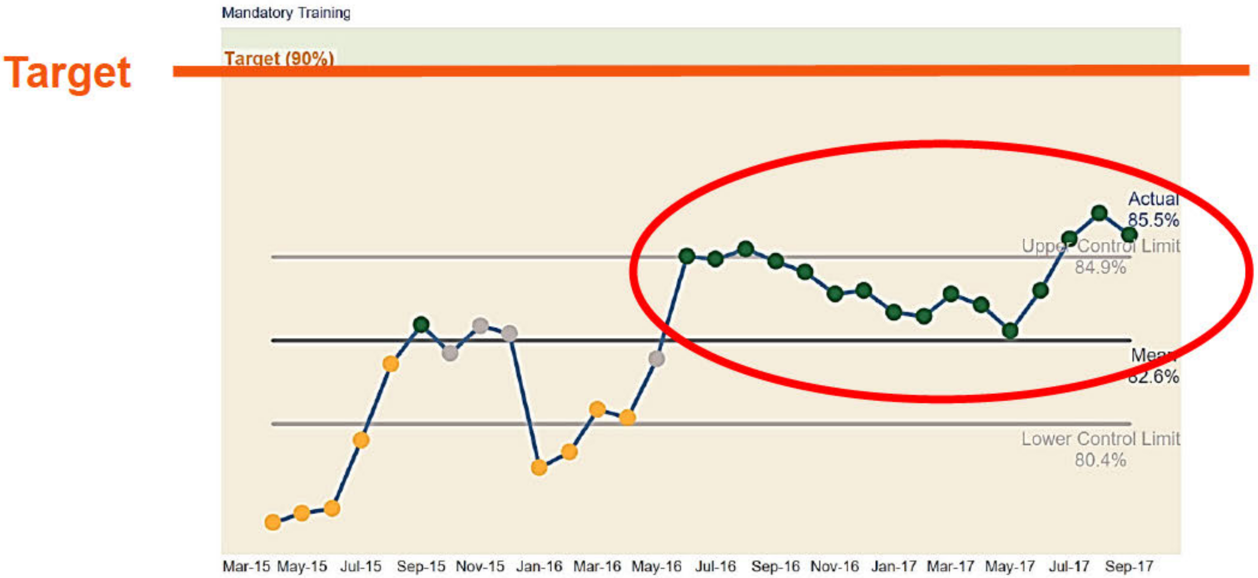

20 | Making data count

NHS Improvement

Are things improving?

Patient Experience Dashboard

Domain	Indicator	Combined Trust Performance					Trust data 13 months	
		Jul-17	Aug-17	Sep-17	2017-2018 Q2	2017-2018	Trend charts	
Friends and Family	FFT: A&E recommend % (Target: >90%)	84.7%	83.5%	84.4%	84.2%	84.6%	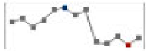	!

Friends and Family Test - A&E recommend %

The recommend rate improved from the previous month however remains below the 90%.

SPC changes the narrative

NHS Improvement

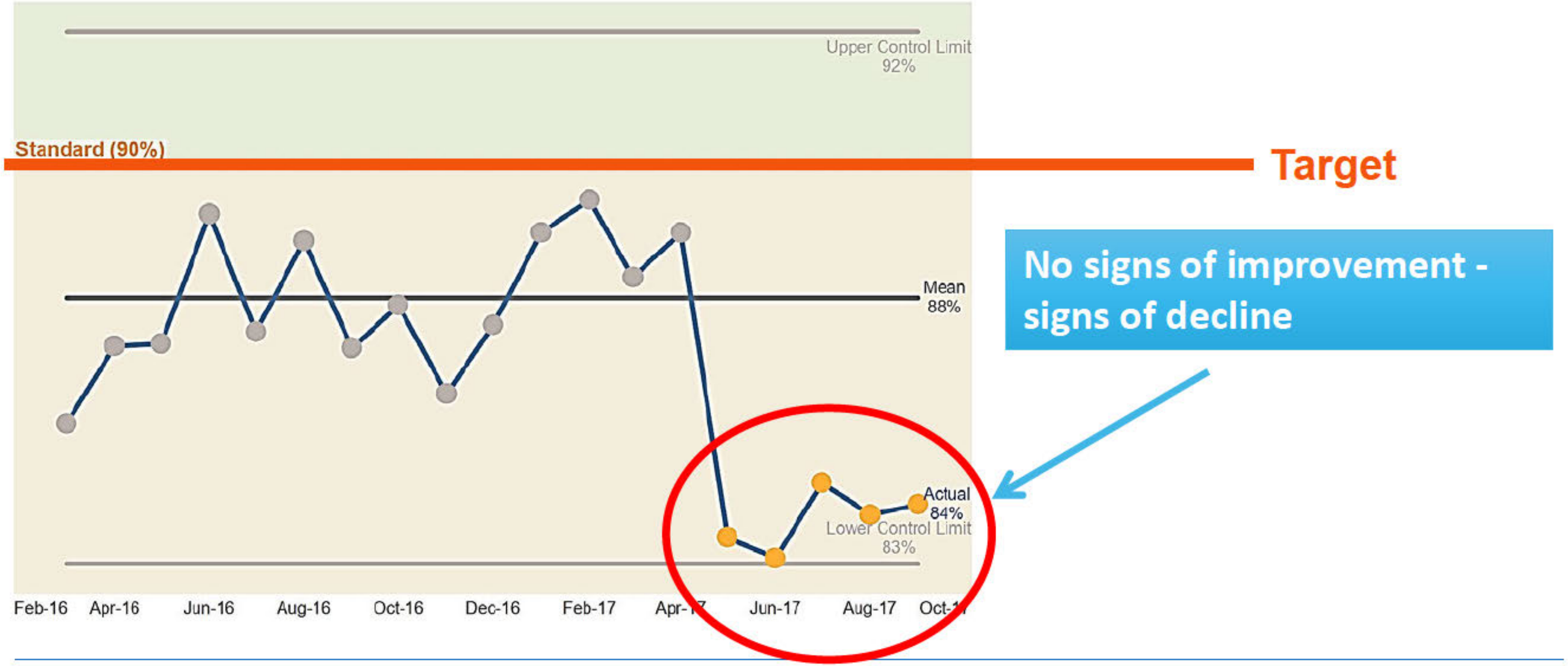

22 | Making data count

Serious incidents

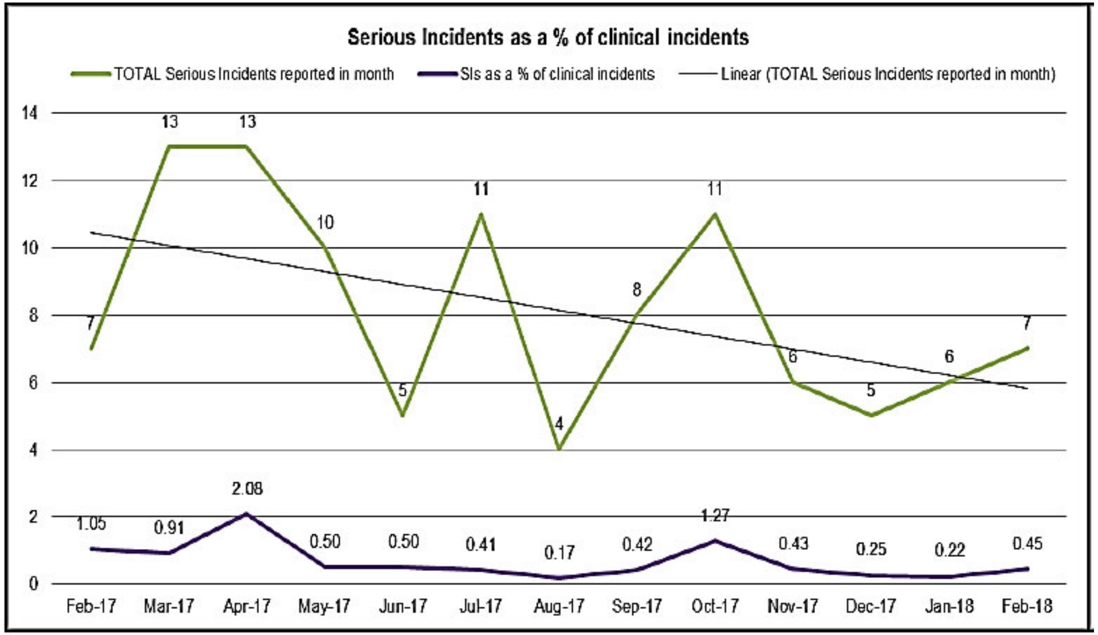

NHS Improvement

Poll 2

The number of serious incidents occurring is :

- Improving
- Declining
- Staying the same

collaboration trust respect innovation courage compassion

Level of variation acceptable?

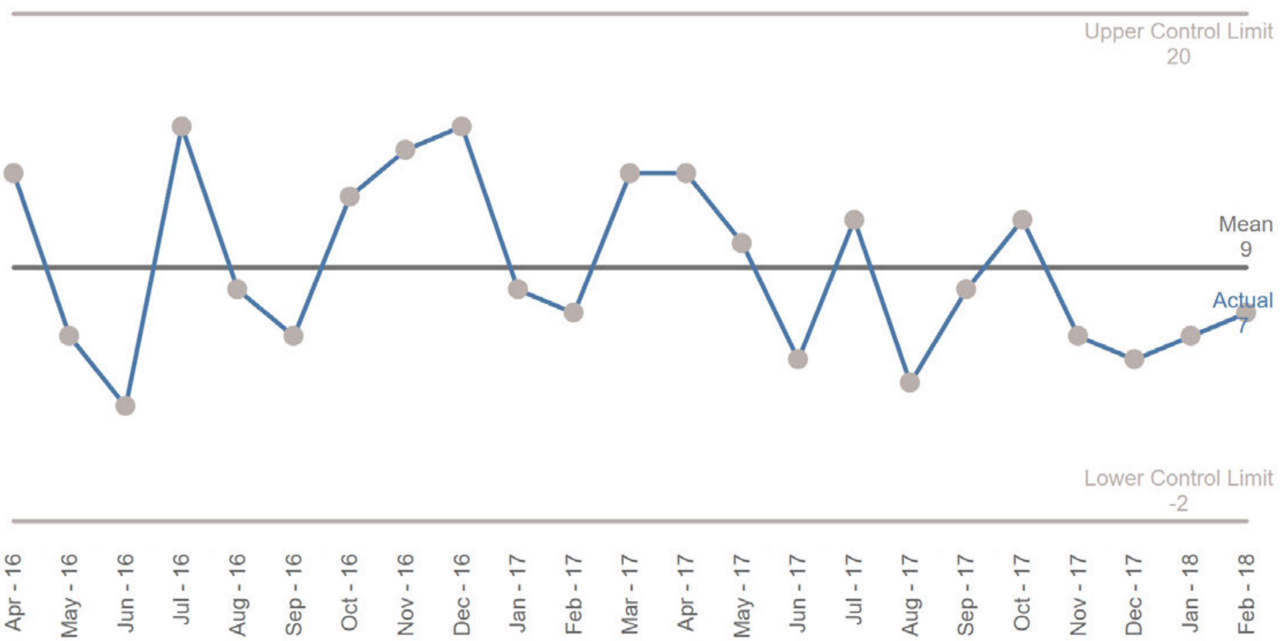

Will the target always be achieved?

NHS Improvement

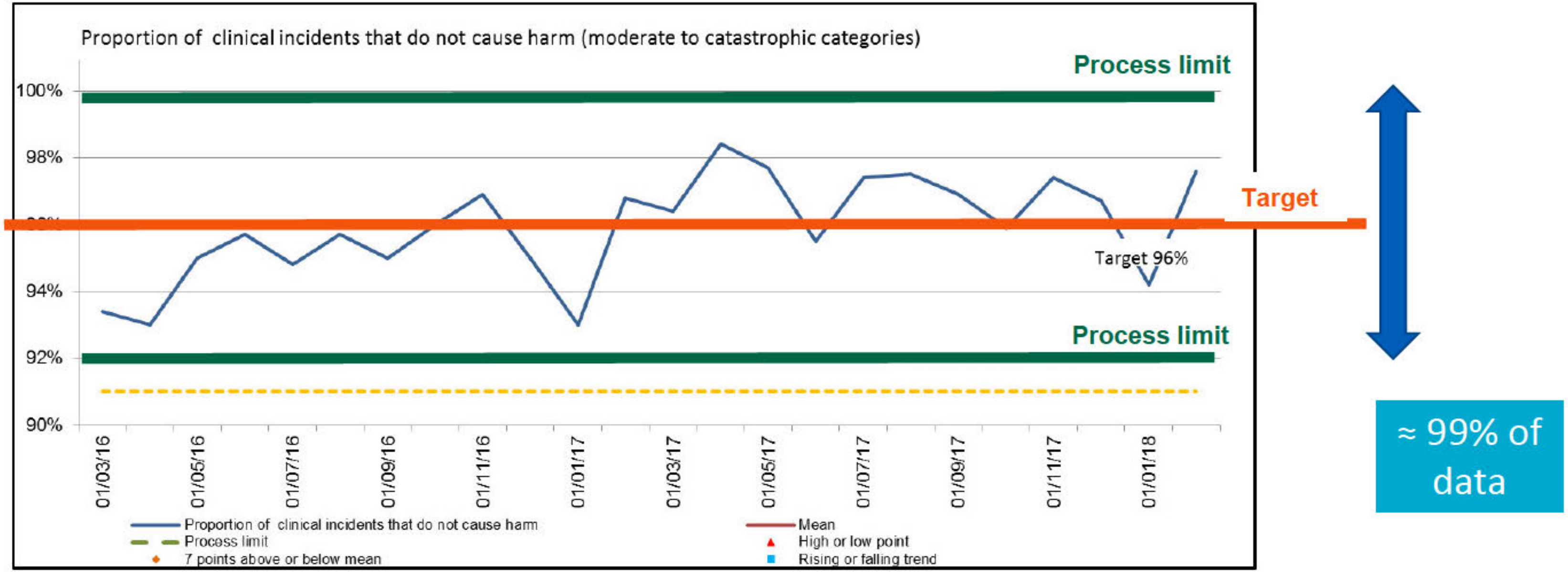

Thinking outside the box

NHS Improvement

University Hospital NH

A & E 4 hour performance

Variation Indica

Special Cause Concern High Low | Special Cause Note/Investiga High Low

Variation Indicators: Special Cause Concern High Low; Special Cause Note/investigate High Low; Common Cause

				Target	Mean	Variance	Comment
Arrivals	A & E 4 hour performance	Dec 17	73	95	83	Higher	[illegible]
Arrivals	Arrivals non ambulance	Nov 17	11,581		10,982	Similar	
Arrivals	Arrivals ambulance	Nov 17	3,191		3,090	Similar	
Arrivals	Ambulance handovers over 30mins	Nov 17	67		75	Similar	[illegible]
Assessment and Treatment	Average Duration (mins) to initial assessment - non Ambulance	Nov 17	23		25	Similar	[illegible]
Assessment and Treatment	Brought in by ambulance (including helicopter/'air ambulance') Average Duration (mins)	Nov 17	16		15	Similar	
Assessment and Treatment	Average Duration (mins) to treatment - non Ambulance	Nov 17	106		98	Similar	[illegible]
Assessment and Treatment	Brought in by ambulance (including helicopter/'air ambulance')Avg. Duration_To_Treat..	Nov 17	115		126	Similar	
Assessment and Treatment	Time from treat to departure	Nov 17	196		197	Similar	
Assessment and Treatment	Average wait per breach - crowding	Nov 17	367		404	Similar	[illegible]
Assessment and Treatment	% of re-attenders within 7 days	Nov 17	11		10	Similar	[illegible]
Assessment and Treatment	Breaches	Oct 17	1,447		1,827	Similar	
Assessment and Treatment	Breach admitted	Oct 17	51		42	Similar	
Assessment and Treatment	Breach not admitted	Oct 17	49		58	Similar	
Admissions and inpatient stay	GP admissions	Oct 17	32		59	Similar	[illegible]
Admissions and inpatient stay	AE admissions	Oct 17	1,814		1,676	Similar	[illegible]
Admissions and inpatient stay	Conversion rate	Nov 17	21		17	Similar	[illegible]
Admissions and inpatient stay	Admissions for avoidable conditions	Oct 17	1,846		1,736	Similar	
Admissions and inpatient stay	Delayed transfers of Care (patients overall)	Oct 17	182		312	Similar	
Admissions and inpatient stay	Stranded Patients (number of patients 18 years and over who have been in hospital ov..	Nov 17	51		53	Similar	
Admissions and inpatient stay	LOS 90th centile excluding zero LOS	Nov 17	9		16	Similar	[illegible]
Admissions and inpatient stay	Patients in bed at midnight	Oct 17	15,601		15,889	Similar	
Admissions and inpatient stay	Patients discharged to their Usual Place of Residency	Oct 17	87		89	Similar	[illegible]
Admissions and inpatient stay	% discharged over weekend	Nov 17	16		19	Similar	
Admissions and inpatient stay	Emergency re-admissions within 30 days following an elective or emergency spell at th..	Nov 17	16		21	Similar	[illegible]
Other	Staff sickness	Nov 17	214		228	Similar	
Other	A&E Scores from Friends and Family Test - % positive	Oct 17	58		58	Higher	
Other	Inpatient Scores from Friends and Family Test - % positive	Oct 17	94		94	Higher	

nt

gate to understand the cause..

reliably

BMJ Publishing Group Limited (BMJ) disclaims all liability and responsibility arising from any reliance placed on this supplemental material which has been supplied by the author(s)

SPC Appendix

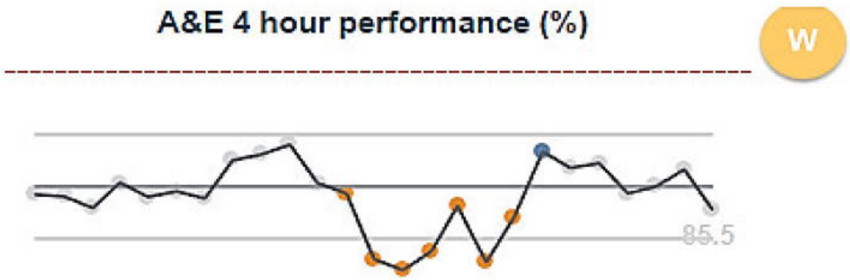

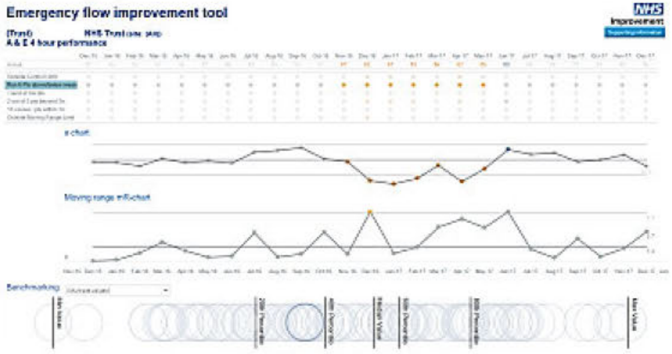

Supporting contextual commentary

Kudrna L, *et al. BMJ Qual Saf* 2023; 32:100–108. doi: 10.1136/bmjqs-2021-013514

What is changing?

NHS Improvement

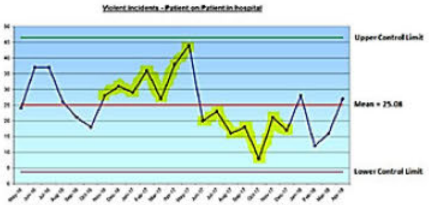

There were 74 patient on staff violent incidents reported trust wide. SPC analysis shows that this is a special cause variation as it is outside of the predicated range for the number of monthly incidents.

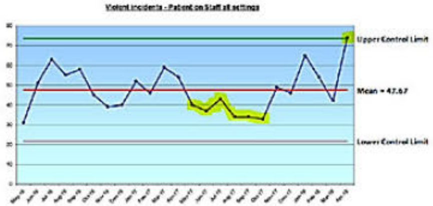

Further analysis shows that both the number of incidents on Haven Ward and Haven incidents as a proportion of total incidents is increasing. When Haven Ward is excluded from the total figures for the Trust the number of incidents is stable and predictable; it will range between 14.87 and 61.13 with a mean of 38.

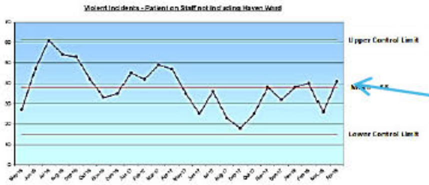

During April one violent patient on staff incident was a moderate incident on Haven Ward and will be subject to a Serious Incident Investigation. A staff member was assaulted by a patient following a Section 136 from Police custody. The staff member has since returned to work, well supported by wider team/management. The rest were low or no harm incidents. Haven and St Brelades were the top two reporters with Haven reporting 33 incidents and St Brelades 20. SPC analysis for St Brelades shows that April's figure is within predicted range of variation (0 to 21.95). On St Brelades Ward one patient was involved in seven incidents; the same patient was involved in three of the patient on patient incidents as well.

SPC analysis for patient on staff violent incidents on Haven shows that this is special cause variation (part run of 6 above mean).

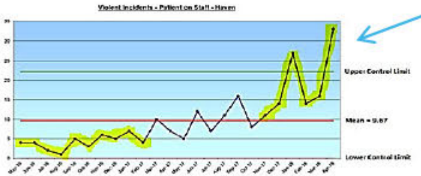

During April two patients on Haven were involved in 26 incidents. The majority of incidents were caused by three patients who were/are acutely unwell. The rest of the violence towards staff was one-off incidents by individual patients. Following the weekly incident reviews in place on Haven, the team identified the individuals concerned, conducted RCA's and organised risk huddles with all team members to create behaviour support plans in order to

SPC charts

Highlighting special cause

Supporting narrative

BMJ Publishing Group Limited (BMJ) disclaims all liability and responsibility arising from any reliance placed on this supplemental material which has been supplied by the author(s)

Dorset Healthcare's SPC Journey

Improvement

[redacted]

Kudrna L, *et al. BMJ Qual Saf* 2023; 32:100–108. doi: 10.1136/bmjqs-2021-013514

BMJ Publishing Group Limited (BMJ) disclaims all liability and responsibility arising from any reliance placed on this supplemental material which has been supplied by the author(s)

NHS Improvement

Advice to others

[redacted]

BMJ Publishing Group Limited (BMJ) disclaims all liability and responsibility arising from any reliance placed on this supplemental material which has been supplied by the author(s)

NHS Improvement

Don’t forget the PORC

In the excitement of introducing SPC and putting control limits on your charts don’t lose sight of the utility and accessibility of the ‘Plain Ole Run Chart’ (PORC)

[redacted]

Kudrna L, *et al. BMJ Qual Saf* 2023; 32:100–108. doi: 10.1136/bmjqs-2021-013514

BMJ Publishing Group Limited (BMJ) disclaims all liability and responsibility arising from any reliance placed on this supplemental material which has been supplied by the author(s)

Top table exclusive

The top table at the feast always used to get the best food. Are SPC and Run Charts seen as rich fare only for the nobs on the top table? Are they routinely used in the front-line?

[redacted]

Kudrna L, *et al. BMJ Qual Saf* 2023; 32:100–108. doi: 10.1136/bmjqs-2021-013514

BMJ Publishing Group Limited (BMJ) disclaims all liability and responsibility arising from any reliance placed on this supplemental material which has been supplied by the author(s)

New hammer syndrome

To someone with a new hammer everything looks like a nail! Not everything is appropriate for SPC or a Run Chart

[redacted]

Kudrna L, *et al. BMJ Qual Saf* 2023; 32:100–108. doi: 10.1136/bmjqs-2021-013514

BMJ Publishing Group Limited (BMJ) disclaims all liability and responsibility arising from any reliance placed on this supplemental material which has been supplied by the author(s)

NHS Improvement

Cargo Cults

Measure it and something will happen. More about Cargo Cults here: https://en.wikipedia.org/wiki/Cargo_cult

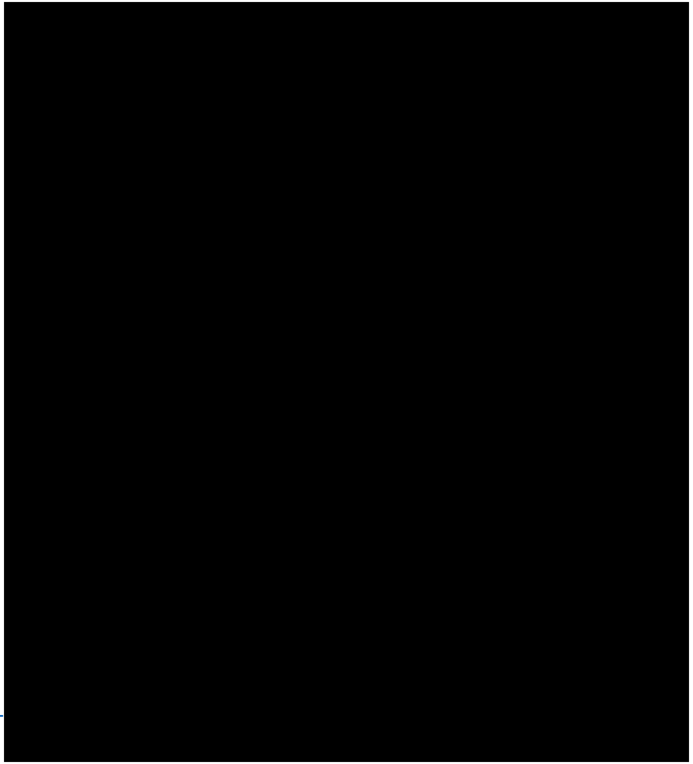

Kudrna L, *et al. BMJ Qual Saf* 2023; 32:100–108. doi: 10.1136/bmjqs-2021-013514

Cargo cults – an example

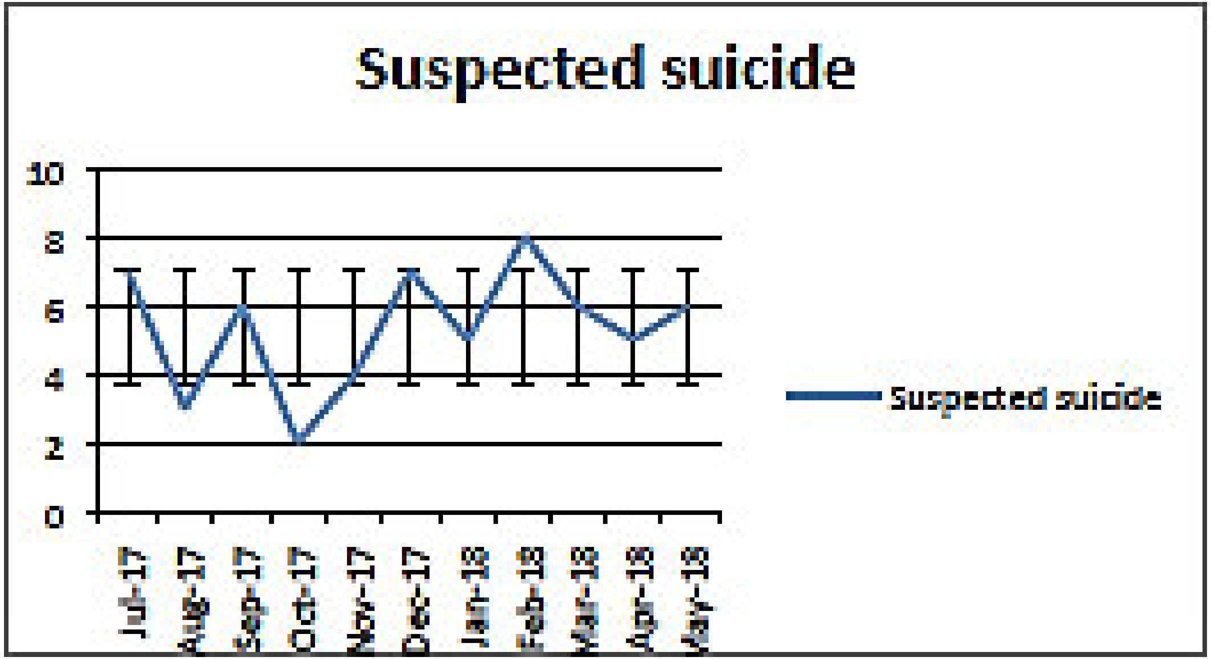

Kudrna L, *et al. BMJ Qual Saf* 2023; 32:100–108. doi: 10.1136/bmjqs-2021-013514

NHS Improvement

Where’s Wally?

Just how many charts can you cram onto an A4 page? If you cannot even read the legend without a magnifying glass then what is the point? How do you identify the chart(s) that indicate significant change in that crowd?

[redacted]

BMJ Publishing Group Limited (BMJ) disclaims all liability and responsibility arising from any reliance placed on this supplemental material which has been supplied by the author(s)

How many angels on the head of the SPC pin?

Watch the newly minted SPC experts start to argue about how many points constitute a shift, a trend, a run – how many points to calculate control limits, sampling etc

[redacted]

Kudrna L, *et al. BMJ Qual Saf* 2023; 32:100–108. doi: 10.1136/bmjqs-2021-013514

BMJ Publishing Group Limited (BMJ) disclaims all liability and responsibility arising from any reliance placed on this supplemental material which has been supplied by the author(s)

[redacted]

BMJ Publishing Group Limited (BMJ) disclaims all liability and responsibility arising from any reliance placed on this supplemental material which has been supplied by the author(s)

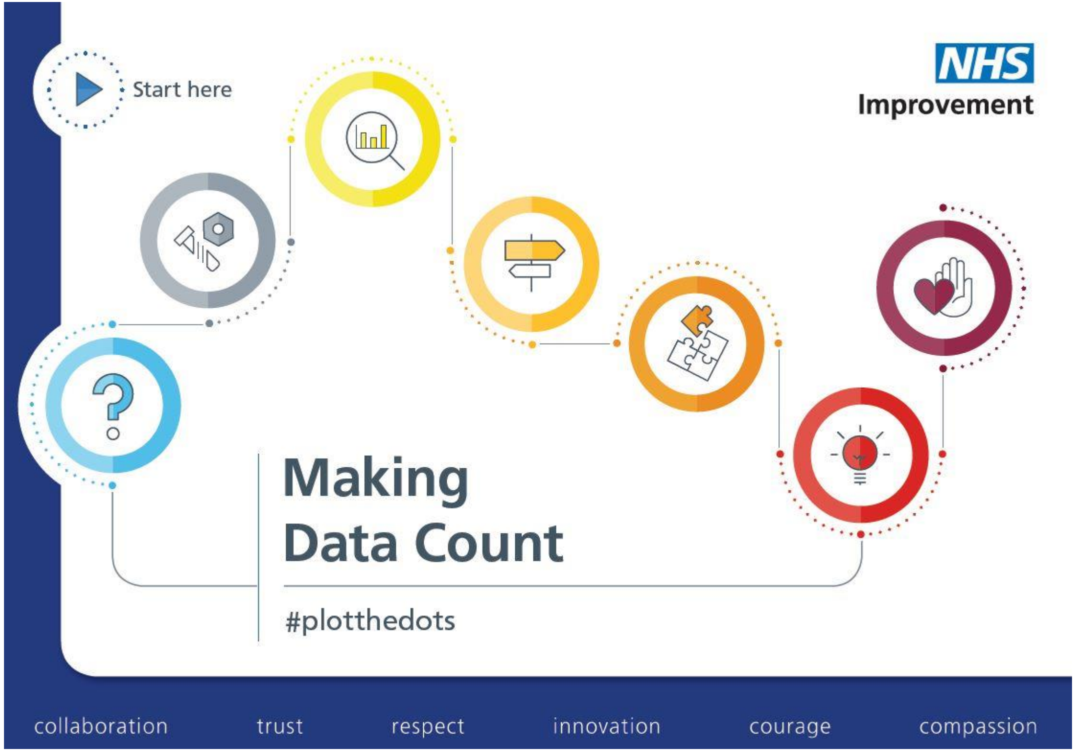

https://improvement.nhs.uk/documents/2748/NHS_MAKING_DATA_COUNT_FINAL.pdf

Kudrna L, *et al. BMJ Qual Saf* 2023; 32:100–108. doi: 10.1136/bmjqs-2021-013514

BMJ Publishing Group Limited (BMJ) disclaims all liability and responsibility arising from any reliance placed on this supplemental material which has been supplied by the author(s)

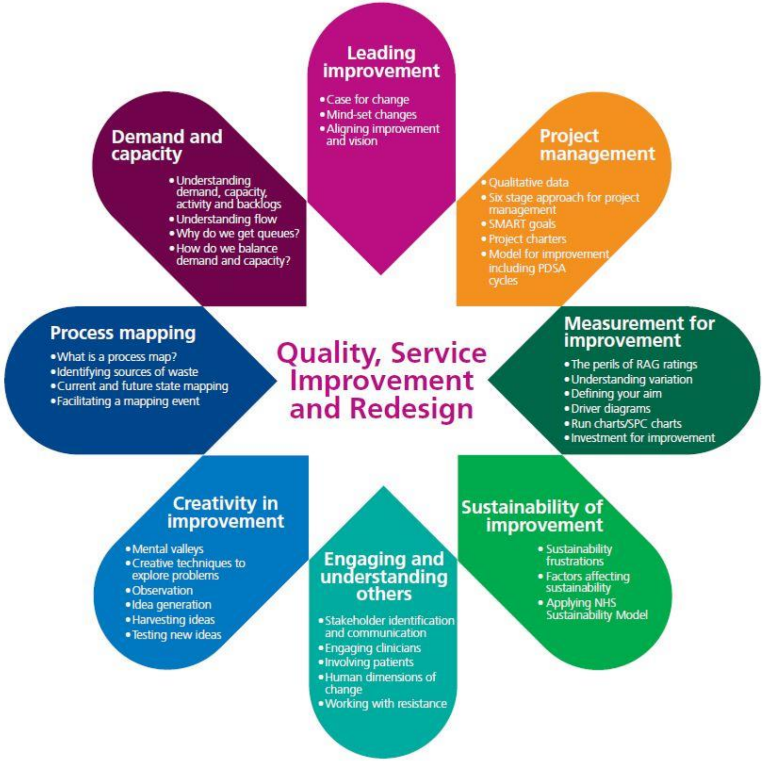

https://improvement.nhs.uk/documents/1241/QSIR-A5-4pp.pdf

Poll 3

Which statement best describes how you feel about your performance report:

- I am confident that my report supports effective decision making
- I am concerned that my report may not focus discussion on the most important issues
- I need time to reflect on today's session

collaboration trust respect innovation courage compassion

NHS Improvement

Poll 4

Please rank the following in order of priority – which of these will be most helpful?

- Test a different approach to regulation
- Implement a regional train the trainer programme
- Establish regional networks
- Facilitate mechanisms to share learning
- Providing analytical products to aid decision making

collaboration trust respect innovation courage compassion

BMJ Publishing Group Limited (BMJ) disclaims all liability and responsibility arising from any reliance placed on this supplemental material which has been supplied by the author(s)

NHS
Improvement

[redacted]

44

Kudrna L, *et al. BMJ Qual Saf* 2023; 32:100–108. doi: 10.1136/bmjqs-2021-013514
