# Supplementary material for: Retrospective evaluation of an intervention based on training sessions to increase the use of control charts in hospitals
Source: BMJ Qual Saf. 2022 Jun 24;32(2):100–8. doi: 10.1136/bmjqs-2021-013514 (PMC9887349; doi:10.1136/bmjqs-2021-013514)
Supplement: Supplementary data [file bmjqs-2021-013514supp006.pdf]

Supplementary 6 – Coding Frame and Selected Results

Coding Frame

The coding frame is shown in Table S6-1. We identified if the charts were time series, between groups, time series and between groups, pie charts, other charts, and/or statistical process control charts (SPCs).

We identified if aspects of the training were reflected in the charts and board papers. One aspect of the training was R-A-G colouring anywhere on the board paper (see slide 7, Supplemental File 4). R-A-G charts consist of tables of coloured boxes that show whether data fail to meet a specific target (red), are in danger of not meeting that target (amber), or are achieving that target (green).[1] The colour coding is not informed by statistical variation. In contrast, SPCs have set statistical limits, typically at two or three standard deviations above and below the mean value.[2–4] The training discusses the limitations of R-A-G charts.

The training encourages hospitals to use SPC icons (slide 47, Supplemental File 4) that summarise statistical variation visually using colours and letters that indicate special or common cause variation and performance relative to a target. We identified if any SPC icons were present on the SPCs.

For the SPCs, we also identified if other factors included in the training were present (see Powerpoint slides in Supplemental File 4):

- Labelling of limits (slides 32, 34), including sigma versus standard deviation control limits. Sigma and standard deviation limits are different because sigma calculations do not assume homogeneity in the underlying data (for example, that they are

derived from an unchanged process), whereas standard deviation calculations assume a constant mean value [2–4];

- recalculation of control limits (slides 44-46), which occurs when the formula used to calculate the control limits is altered due to a change in the underlying process, such as a new bed management system;
- highlighted runs or trends (slides 25-27);
- and comments about reasons for variation or suggestions for improvement (slide 47).

Table S6-1 – Coding Frame

**Chart Number**
1. Is RAG present?
2. Type of chart: time series?
3. Type of chart: between groups?
4. Type of chart: time series and between groups?
5. Type of chart: pie chart?
6. Type of chart: other chart?
7. Type of chart: notes on other
8. Is this a statistical process control chart summary icon?
9. Is this a statistical process control chart?
IF YES statistical process control chart:
10a. Are the control limits labelled?
10b. If labelled are the control limits labelled as sigma?
10c. One sigma?
10d. Two sigma?

Supplementary 6 – Coding Frame and Selected Results

10e. Three+ sigma?
10f. If labelled are the control limits labelled as standard deviation?
10g. One standard deviation?
10h. Two standard deviations?
10i. Three+ standard deviations?
11. Control limits recalculated?
12a. Run/trend highlighted?
12b. Run/trend 7+ points?
12c. Run/trend <7 points?
13. Does the chart have comments about reasons for variation?
14. Does the chart have suggestions for intervention?
15. Notes:

Response options to all except items 7 and 15 are either ‘yes’ or ‘no’. Items 1-9 are filled out for all charts. Items 10-14 are only filled out for SPCs. Items 10b and 10f are only answered if the response to 10a is ‘yes’. Items 10c-e are only answered if the response to 10b is ‘yes’. Items 10g-I are only answered if the response to 10f is ‘yes’. Items 12b-c are only answered if the response to 12a is ‘yes’. RAG is captured at the board paper rather than chart level an aspect of the training was focussed on reducing RAG colouring more generally.

Selected Results

The main results for time series, between group, and SPCs are reported in the main manuscript. Considering all of the charts identified, there were also 129/6,287 (2%) pie charts

and 46/6,287 (1%) 'other' charts. Of the 46 'other' charts, 27/46 were donuts (59%), 9/46 were heat maps (20%), 4/46 were population pyramids (9%), 2/46 were scatterplots (4%), 3/46 were spider diagrams (7%), and 1/46 was a people plot (2%). RAG colouring was used by 18 of the 20 hospitals (90% of hospitals) at least once in their board papers.

Around half of all charts were contained within dashboards (3,348/6,287, 53%). Dashboards are Tables of data, including Tables of charts (an example is in Supplemental File 4, slide 2).

Further information specific to the 449 SPCs identified is shown in Table S6-2. The control limits were labelled for 342/449 (76%) of the SPCs. Considering only those 342 SPCs with labelled limits, sigma limits (139/342, 41%) were more common than standard deviation limits (12/342, 4%). However, it was most common that the labelled limits were not labelled as either sigma or standard deviation (191/342, 43%), using text such as UCL ('upper confidence limit') or LCL ('lower confidence limit').

Considering all 449 SPCs, half of the SPCs highlighted if a run or trend was present (215/449, 48%). Around one quarter included comments about reasons for variation (123/449, 28%) or suggestions for improvement (109/449, 24%). A minority recalculated the control limits (59/449, 13.2%). Note that we do not inspect differences between intervention and control hospitals on the characteristics of SPCs due to the small overall number of SPCs identified.

Supplementary 6 – Coding Frame and Selected Results

Table S6-2

Chart characteristics (SPCs)

Characteristic	SPCs (n=449) n (%)
**Control limit labelled**	342 (76.2)
**Sigma limit**	139 (31.0)
One sigma	0
Two sigma	0
Three sigma	139 (40.6)
**Standard deviation limit**	12 (3.5)
One standard deviation	0
Two standard deviation	12 (3.5)
Three standard deviation	12 (3.5)
**Not sigma or standard deviation**	191 (42.5)
**Control limits recalculated**	59 (13.2)
**Run/trend highlighted**	215 (48.0)
**Comments about variation**	123 (27.5)
**Suggestions for improvement**	109 (24.4)

References

1 Anhøj J, Hellesøe AMB. The problem with red, amber, green: The need to avoid distraction by random variation in organisational performance measures. BMJ Qual. Saf. 2017;**26**:81–4. doi:10.1136/bmjqs-2015-004951

2 Wheeler DJ. The Right and Wrong Ways of Computing Limits. *Qual Dig* Published Online First: 2010.https://www.qualitydigest.com/inside/six-sigma-column/right-and-wrong-ways-computing-limits.html

3 Wheeler DJ. Contra Two Sigma. 2013. www.spcpress.com/pdf/DJW255.pdf1 (accessed 29 Jan 2021).

4 Banchs RJ, Pop MR. *The quality improvement challenge : a practical guide for physicians*. Oxford: Wiley Blackwell
