# Supplementary material for: Retrospective evaluation of an intervention based on training sessions to increase the use of control charts in hospitals
Source: BMJ Qual Saf. 2022 Jun 24;32(2):100–8. doi: 10.1136/bmjqs-2021-013514 (PMC9887349; doi:10.1136/bmjqs-2021-013514)
Supplement: Supplementary data [file bmjqs-2021-013514supp007.pdf]

Supplementary 7- Sensitivity Analyses

Sensitivity analyses

Firstly to allow for full information on the pre-intervention number of SPCs (as opposed to condensing this information into a proportion as in our primary analysis) a Poisson regression model was fitted with an offset for the total number of charts and the outcome as the number of charts presented as an SPC, and with adjustment for group (intervention or control group), for period (pre or post intervention exposure) and an interaction between group and period (treatment effect). To allow for the dependence between repeated measures on the same hospital a random cluster effect (without any small sample correction, which is not supported in Stata 16 for count or binary outcomes) is included. We additionally tried to allow for degree of clustering to depend on period of measurement, but models with a random interaction between cluster and period failed to converge. Results are reported on the rate ratio scale with 95% confidence intervals. This is Model 1 in supplementary tables. This model was our planned primary analysis, but to allow for the over dispersion evident in the counts and for the many zero counts, we additionally extended these models to zero-inflated Poisson (Model 2, without a random cluster effect as this is not supported in Stata 16) and negative Binomial (Model 3), with a random hospital effects – again models with random hospital by period effects failed to converge. Due to lack of convergence of the random hospital by period interaction, we additionally fitted these models using logistic regression (this model can have better convergence properties), but this model with the random interaction also failed to converge and so is included with a random hospital effect only (Model 4).

Finally, to avoid reliance on mixed models (which might not be stable with only 20 hospitals especially without a small sample corrections) we additionally modelled the data conditioning on the proportion of charts which were SPCs in the pre-intervention period (as a fixed categorical effect) using a Poisson distribution (Model 5), negative Binomial (Model 6),

zero-inflated Poisson (Model 7) and zero-inflated negative Binomial (Model 8, our primary model). All models suggest a positive and large impact of the intervention, with the exception of model 1 to 3 (these models are not expected to be very reliable, as they use a random effect with only 20 clusters and does not allow for the over dispersion and zero counts which are prominent features of the data).

Supplementary 7- Sensitivity Analyses

Table S7-1 Different model approaches for primary analysis

	**Model 1 – Mixed effects Poisson model**	**Model 2 – Zero inflated Poisson model**	**Model 3 – Mixed effects negative Binomial model**	**Model 4 – Mixed effects logistic model**
	**Rate ratio (95% CI)**	**Rate ratio (95% CI)**	**Rate ratio (95% CI)**	**Odd ratio (95% CI)**
**Group – intervention**	14.61 (2.03 to 104.86)	4.46 (2.07 to 9.60)	7.49 (1.19 to 47.18)	13.96 (1.62 to 120.50)
**Period – post intervention**	1.76 (0.81 to 3.85)	1.81 (0.77 to 4.21)	1.46 (0.22 to 9.50)	1.79 (0.81 to 3.96)
**Treatment effect - group # period**	1.84 (0.81 to 4.18)	1.19 (0.491 to 2.86)	2.81 (0.22 to 35.29)	3.19 (1.36 to 7.48)
**Changes**	Original analysis plan	Model used adjusts for zero cells in outcome	Model used adjusts for overdispersion in outcome	Model treats outcome as binomial not count data
**Outcome**	Number of SPCs	Number of SPCs	Number of SPCs	Number of SPCs
**Covariates**	Group (intervention or control), period (pre/post intervention) and treatment effect	Group (intervention or control), period (pre/post intervention) and treatment effect	Group (intervention or control), period (pre/post intervention) and treatment effect	Group (intervention or control), period (pre/post intervention) and treatment effect
**Random effect**	Yes, hospital	No	Yes, hospital	Yes, hospital
**Comments**	**Issue using random effects for small number of clusters (hospital) and doesn't adjust for zero cells in outcome**	**This model adjusts for zero cells in outcome but not clusters (hospital).**	**Issue using random effects for small number of clusters (hospital)**	**Issue using random effects for small number of clusters (hospital)**

Supplementary 7- Sensitivity Analyses

	**Model 5 – Poisson model**	**Model 6 – Negative Binomial model**	**Model 7 – Zero inflated Poisson model**	**Model 8 – Zero inflated negative Binomial model**
	**Rate ratio (95% CI)**	**Rate ratio (95% CI)**	**Rate ratio (95% CI)**	**Rate ratio (95% CI)**
**Group – intervention**	14.71 (9.58 to 22.58)	17.90 (3.63 to 88.3)	4.71 (3.03 to 7.31)	9.24 (2.68 to 31.87)
**Pre-measurement**	10.23 (4.42 to 23.65)	5.66 (0.01 to 6258.06)	4.96 (2.03 to 12.13)	3.24 (0.10 to 100.30)
**Changes**	Data is reshaped to account for repeated measurements and avoid reliance on mixed models	Data is reshaped to account for repeated measurements and avoid reliance on mixed models and model used adjusts for overdispersion	Data is reshaped to account for repeated measurements and avoid reliance on mixed models and model used adjusts for zero cells.	Data is reshaped to account for repeated measurements and avoid reliance on mixed models and model used adjusts for zero cells and overdispersion.
**Outcome**	Number of SPCs in post-intervention measurements	Number of SPCs in post-intervention measurements	Number of SPCs in post-intervention measurements	Number of SPCs in post-intervention measurements
**Covariates**	Group (intervention or control) and pre-intervention proportion of SPCs.	Group (intervention or control) and pre-intervention proportion of SPCs.	Group (intervention or control) and pre-intervention proportion of SPCs.	Group (intervention or control) and pre-intervention proportion of SPCs.
**Comments**	**No adjustment made for zero cells or overdispersion.**	**This model adjusts for overdispersion in outcome but not zero cells.**	**This model adjusts for zero cells in outcome but not overdispersion.**	**This model adjusts for zero cells and overdispersion in outcome, out primary model.**

Supplementary 7- Sensitivity Analyses

Table S7-2 SPC usage by group, hospital and period out of time series charts

Control group				Intervention group			
**Hospital**	**Pre-training** SPC/Chart (%)	**Post-training** SPC/Chart (%)	**Post– Pre** % difference	**Hospital**	**Pre-training** SPC/Chart (%)	**Post-training** SPC/Chart (%)	**Post– Pre** % difference
**1**	0/57 (0)	0/69 (0)	0	**11**	0/190 (0)	9/184 (5)	5
**2**	0/71 (0)	0/97 (0)	0	**12**	0/149 (0)	0/117 (0)	0
**3**	0/12 (0)	2/53 (4)	4	**13**	0/109 (0)	0/77 (0)	0
**4**	0/638 (0)	0/665 (0)	0	**14**	3/115 (3)	91/243 (37)	34
**5**	0/146 (0)	0/163 (0)	0	**15**	52/107 (49)	47/63 (75)	26
**6**	0/78 (0)	11/155 (7)	7	**16**	0/69 (0)	58/81 (72)	72
**7**	0/138 (0)	0/137 (0)	0	**17**	0/11 (0)	27/52 (52)	52
**8**	0/92 (0)	0/93 (0)	0	**18**	18/137 (13)	42/404 (10)	-3
**9**	2/148 (1)	6/178 (3)	2	**19**	0/80 (0)	25/80 (31)	31
**10**	0/99 (5)	0/85 (0)	0	**20**	8/112 (7)	20/93 (22)	15
**Average difference in control group (95% CI)**			0 (0 to 2)	**Average difference in intervention group (95% CI)**			19 (7 to 30)
				**Average difference between intervention and control group* (95% CI)**			18 (7 to 29)
				**Average rate change between intervention and control group ** (95% CI)**			9 (3 to 29)

For each hospital in pre and post intervention period, the number of SPCs, the number of all charts and percentage of SPCs out of time series charts are reported

* T-test comparing average difference in proportions between intervention and control group. Percentage difference and 95% confidence intervals are reported.

** Zero-inflated negative Binomial regression models. Outcome is number of SPCs in post-intervention period, adjusting for pre-intervention proportion of SPCs. Exposure is time series charts. Rate ratios and 95% confidence intervals are reported.

Supplementary 7- Sensitivity Analyses

Table S7-3 SPC usage by group, hospital and period out of time series and between group charts

Control group				Intervention group			
**Hospital**	**Pre-training** SPC/Chart (%)	**Post-training** SPC/Chart (%)	**Post–Pre** % difference	**Hospital**	**Pre-training** SPC/Chart (%)	**Post-training** SPC/Chart (%)	**Post– Pre** % difference
**1**	0/13 (0)	0/36 (0)	0	**11**	0/13 (0)	0/11 (0)	0
**2**	0/34 (0)	0/51 (0)	0	**12**	0/27 (0)	0/29 (0)	0
**3**	0/4 (0)	0/32 (0)	0	**13**	0/60 (0)	0/43 (0)	0
**4**	0/1 (0)	0/8 (0)	0	**14**	2/25 (8)	1/28 (4)	-4
**5**	0/6 (0)	0/16 (0)	0	**15**	20/29 (69)	17/26 (65)	-4
**6**	0/7 (0)	0/9 (0)	0	**16**	0/42 (0)	18/34 (53)	53
**7**	0/2 (0)	0/1 (0)	0	**17**	0/6 (0)	0/13 (0)	0
**8**	0/38 (0)	0/37 (0)	0	**18**	0/21 (0)	0/37 (0)	0
**9**	0/10 (0)	0/18 (0)	0	**19**	0/21 (0)	0/20 (0)	0
**10**	0/5 (0)	0/1 (0)	0	**20**	0/56 (0)	0/37 (0)	0
**Average difference in control group (95% CI)**			0 (0 to 0)	**Average difference in intervention group (95% CI)**			10 (0 to 21)
				**Average difference between intervention and control group* (95% CI)**			10 (0 to 20)
				**Average rate change between intervention and control group ** (95% CI)**			Non-convergence

For each hospital in pre and post intervention period, the number of SPCs, the number of all charts and percentage of SPCs out of time series and between group charts are reported

* T-test comparing average difference in proportions between intervention and control group. Percentage difference and 95% confidence intervals are reported.

** Zero-inflated negative Binomial regression models. Outcome is number of SPCs in post-intervention period, adjusting for pre-intervention proportion of SPCs. Exposure is time series and between group charts. Rate ratios and 95% confidence intervals are reported.
