# Supplementary material for: Retrospective evaluation of an intervention based on training sessions to increase the use of control charts in hospitals
Source: BMJ Qual Saf. 2022 Jun 24;32(2):100–8. doi: 10.1136/bmjqs-2021-013514 (PMC9887349; doi:10.1136/bmjqs-2021-013514)
Supplement: Supplementary data [file bmjqs-2021-013514supp008.pdf]

Supplementary 8 – Discussion of Proportional Changes

There are several mechanisms by which the proportional changes in this study could be brought about. Firstly, as intended, charts that were previously not produced using statistical process control methodology could be transformed into SPCs. This scenario is represented in Pathway A in Figure S8-1 below. However, we propose three additional ways that the proportion of SPCs could increase. First, non-SPCs are eliminated, and all SPCs maintained, with no new SPCs generated (Pathway B). Although there are no new SPCs in this scenario, the proportion of SPCs increases only because the denominator decreases. Second, SPCs and non-SPCs are eliminated in equal or similar proportions while some previously non-SPCs are now produced using statistical process control methodology (Pathway C). Increased SPC usage is more ambiguous in this scenario because SPCs have been both eliminated and created alongside a decrease in the denominator. Third, all SPCs are retained, new SPCs are produced, and some non-SPCs are eliminated (Pathway D). Here more SPCs have been produced, but the reduced total number of charts exaggerates the apparent effect of the intervention on converting one type of chart to another. Note that these mechanisms are all possible, at least in theory, even in a randomised controlled trial, since the intervention could have prompted changes in the number of charts presented to boards as well as causing non-SPCs to be converted to SPCs.

We can investigate some of these possibilities by visual inspection of the data in Table 3 (in the main manuscript). There is one case where a drop in the denominator was accompanied an improving SPC ratio with a no substantive increase in the numerator (Trust 15). Here, the proportion could have increased purely because of a decline in the denominator. But we would have to assume that the type of chart that was eliminated was selectively the type that was not already captured in statistical process control form (Figure S8-1, Pathway B). If the SPCs and the non-SPCs both declined, then the only way an overall increase in SPCs could

be observed is through increased adoption of statistical process control methodology (Figure S8-1, Pathway C). There are also two cases in Table 3 where the number of SPCs in the numerator increased, and the denominator also increased (Trust 14, Trust 17). In this case, at least part of the increase could have resulted from adopting the type of chart that lends itself to statistical process control methods.

In theory, it would be possible to evaluate these other mechanisms more precisely with a more time intensive approach tracking longitudinal changes at the level of individual charts rather than aggregating information about charts at the hospital level. However, the same individual charts do not always appear across board papers over time.

<u>Figure S8-1.</u> Four examples of mechanisms by which the proportional changes in this study could occur.

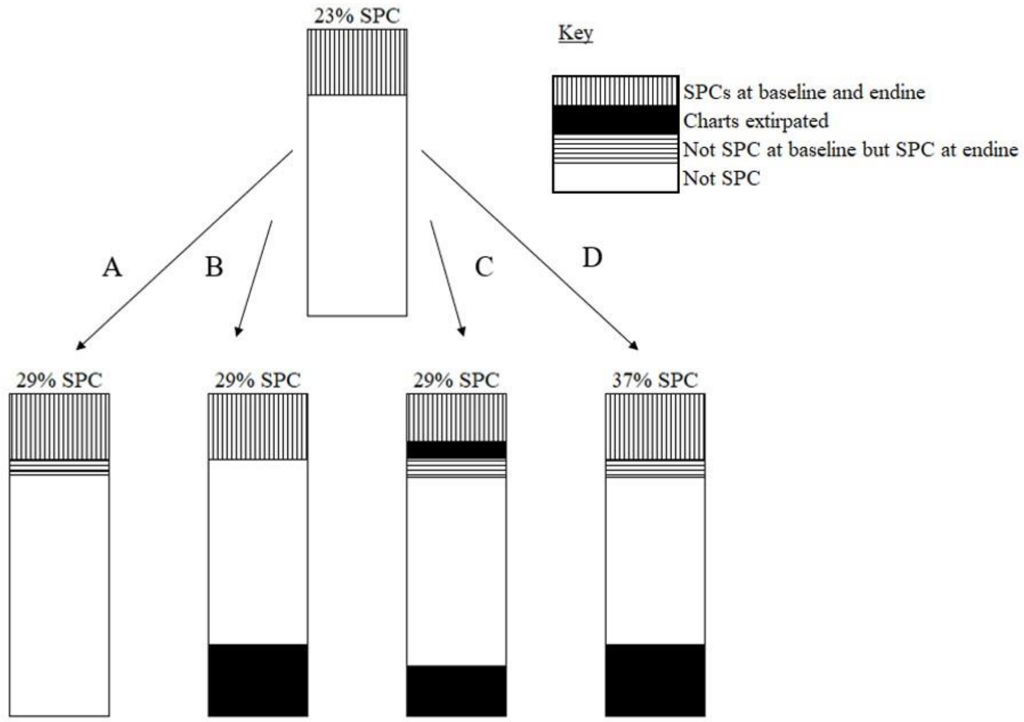

Supplementary 8 – Discussion of Proportional Changes
